# Genome‐wide transcriptomic and proteomic analyses of bollworm‐infested developing cotton bolls revealed the genes and pathways involved in the insect pest defence mechanism

**DOI:** 10.1111/pbi.12508

**Published:** 2016-01-22

**Authors:** Saravanan Kumar, Mogilicherla Kanakachari, Dhandapani Gurusamy, Krishan Kumar, Prabhakaran Narayanasamy, Padmalatha Kethireddy Venkata, Amolkumar Solanke, Savita Gamanagatti, Vamadevaiah Hiremath, Ishwarappa S. Katageri, Sadhu Leelavathi, Polumetla Ananda Kumar, Vanga Siva Reddy

**Affiliations:** ^1^ International Centre for Genetic Engineering and BiotechnologyNew DelhiIndia; ^2^ National Research Centre on Plant BiotechnologyIndian Agricultural Research Institute (IARI)New DelhiIndia; ^3^ University of Agricultural SciencesDharwadIndia; ^4^ Present address: College of AgricultureDepartment of EntomologyS225 Agriculture Science Centre‐NUniversity of KentuckyLexingtonKY40546‐0091USA; ^5^ Present address: Division of Plant PathologyIndian Agricultural Research Institute (IARI)New Delhi110012India; ^6^ Present address: Department of Crop PhysiologyUniversity of Agricultural Sciences (UAS)G.K.V.KBangalore560065KarnatakaIndia; ^7^ Present address: Division of BiotechnologyIndian Institute of Rice Research (IIRR)Hyderabad500030India

**Keywords:** *Gossypium hirsutum*, *Helicoverpa armigera*, biotic stress response, transcriptome, proteome, defence mechanism

## Abstract

Cotton bollworm, *Helicoverpa armigera*, is a major insect pest that feeds on cotton bolls causing extensive damage leading to crop and productivity loss. In spite of such a major impact, cotton plant response to bollworm infection is yet to be witnessed. In this context, we have studied the genome‐wide response of cotton bolls infested with bollworm using transcriptomic and proteomic approaches. Further, we have validated this data using semi‐quantitative real‐time PCR. Comparative analyses have revealed that 39% of the transcriptome and 35% of the proteome were differentially regulated during bollworm infestation. Around 36% of significantly regulated transcripts and 45% of differentially expressed proteins were found to be involved in signalling followed by redox regulation. Further analysis showed that defence‐related stress hormones and their lipid precursors, transcription factors, signalling molecules, etc. were stimulated, whereas the growth‐related counterparts were suppressed during bollworm infestation. Around 26% of the significantly up‐regulated proteins were defence molecules, while >50% of the significantly down‐regulated were related to photosynthesis and growth. Interestingly, the biosynthesis genes for synergistically regulated jasmonate, ethylene and suppressors of the antagonistic factor salicylate were found to be up‐regulated, suggesting a choice among stress‐responsive phytohormone regulation. Manual curation of the enzymes and TFs highlighted the components of retrograde signalling pathways. Our data suggest that a selective regulatory mechanism directs the reallocation of metabolic resources favouring defence over growth under bollworm infestation and these insights could be exploited to develop bollworm‐resistant cotton varieties.

## Introduction

Plants and insects have coexisted for about 350 million years leading to the evolution of both positive and negative interactions (Gatehouse, 2002). Positive interactions include insect‐mediated pollination, seed dispersion, etc. that offer mutual benefit to the insect and the host, while negative interactions include insect predation (Gatehouse, 2002) that often causes detrimental effects to the host. In view of the long standing relationship, it is quite obvious that plants have evolved a diverse set of stress‐specific constitutive and/or inducible defence mechanisms to resist and coexist with the insect pests (Gatehouse, 2002). The response pattern in both the mechanisms might either be activated locally at the infected site, systemically in the uninfected regions or by both the aforementioned through signalling molecules (Gatehouse, 2002). Signal perception and activation results in a vast cascade of events at cellular and molecular levels ultimately contributing to the defence mechanism. Specialized defence mechanisms that protect plants from insects include physical barriers such as cell wall and cuticle (Kempema *et al*., 2007), cellular processes including lignifications, cross‐linking of cell wall components, release of volatile and nonvolatile metabolites (Kempema *et al*., 2007) and molecular processes like activation of defence‐related genes and pathways (Ramirez *et al*., 2009; Ryan, 1990). In addition, hormones, transcription factors (TFs) and redox regulators play major role in stress response and defence signalling. Hormones are secondary signals that amplify primary elicitor signals during biotic stress (Yang *et al*., 1997). Salicylic acid (SA), ethylene (ET), jasmonic acid (JA) and systemin are the major phytohormones that are often quoted as stress‐specific signalling molecules (Arimura *et al*., 2005; Loake and Grant, 2007; Sun *et al*., 2011; Yang *et al*., 1997). Moran and Thompson (2001) suggested that there is a complex crosstalk among hormonal pathways that control the plant responses to wounds, insect pest and pathogen attacks. In addition to hormones, pathogen elicitors also activate TFs that interacts with the pathogen‐responsive cis elements present in the promoters of the defence‐related genes. Even a single pathogen elicitor is capable of activating multiple TFs that can interact with the cis elements present within the same or different promoter regions ultimately leading to stimulation of vast set of defence‐related genes and gene products (Yang *et al*., 1997). Oxidative burst is one of the major processes that occur during biotic stress condition (Lamb and Dixon, 1997). Maintenance of the redox balance within the plant cell plays a crucial role in modulating redox sensitive genes and proteins including many TFs (Torres, 2010). Release of reactive oxygen species (ROS) such as $O_{2}^{-}$ and H_2_O_2_ induces many defence‐related events including cell wall reinforcement through lignifications and cross‐linking of glycoproteins in the extracellular matrix, activation of defence‐related genes, molecules, etc. (Jabs *et al*., 1997). The above‐mentioned cascade of events demand and consume considerable amount of energy. During stress conditions, plant systems manage their biological energy and resources by either partitioning or favouring the molecular machineries towards defence and/or growth. As a result, most plants do survive insect predation; however, it is often accompanied by reduced growth and yield penalty depending on the site of insect predation.

Cotton (*Gossypium* spp.) is the leading contributor of natural fibre and is an important source of textile commodity, oil and protein meal (Han *et al*., 2004; Mei *et al*., 2004). Cotton bolls are crucial tissues that harbour lint (textile fibre) which is of huge economic value. The effect of insect pest infestation followed by secondary infection could lead up to 80% loss in cotton fibre production (Oerke, 2006). Around 1326 species of insects have been reported worldwide as cotton pests, and among them, bollworm (*Helicoverpa armigera*) is the major pest that directly feed and destroy the developing fibre tissue within the cotton bolls (Dua *et al*., 2006; Matthews, 1994). Biotic stress induced through insect pest attack regulates cellular events majorly driven by expression changes of genes and their associated pathways. Earlier efforts to understand plant diseases caused by insect, pathogen infestations have identified certain genes and pathways involved in the biotic stress tolerance in cotton (Artico *et al*., 2014; Dubey *et al*., 2013; Gao *et al*., 2013). Systems level analysis at the transcript and protein levels more often reveals the near‐complete status of an organism subjected to stress or disease conditions (Komatsu *et al*., 2009; Srivastava *et al*., 2013). Such analyses are yet to be employed to understand molecular and cellular mechanisms operational during cotton plant and bollworm interactions. Further understanding of these interactions using high‐throughput approaches might reveal stress‐induced responses and endogenous resistance mechanisms operational in the host. In this context, we have made an attempt to understand the mechanisms adapted by cotton plant during bollworm attack using both transcriptomic and proteomic tools. As bolls are the target site for fibre synthesis as well as bollworm feeding, we have performed comparative analyses of developing cotton bolls subjected to bollworm infestation. Our comprehensive genome‐wide analyses have revealed several new and interesting insights about cotton plant and bollworm interactions. Knowledge gained through this study could be further exploited to develop bollworm‐resistant cotton varieties.

## Results

### Differentially regulated transcripts and proteins in bollworm‐infested cotton bolls

**Figure 1:**
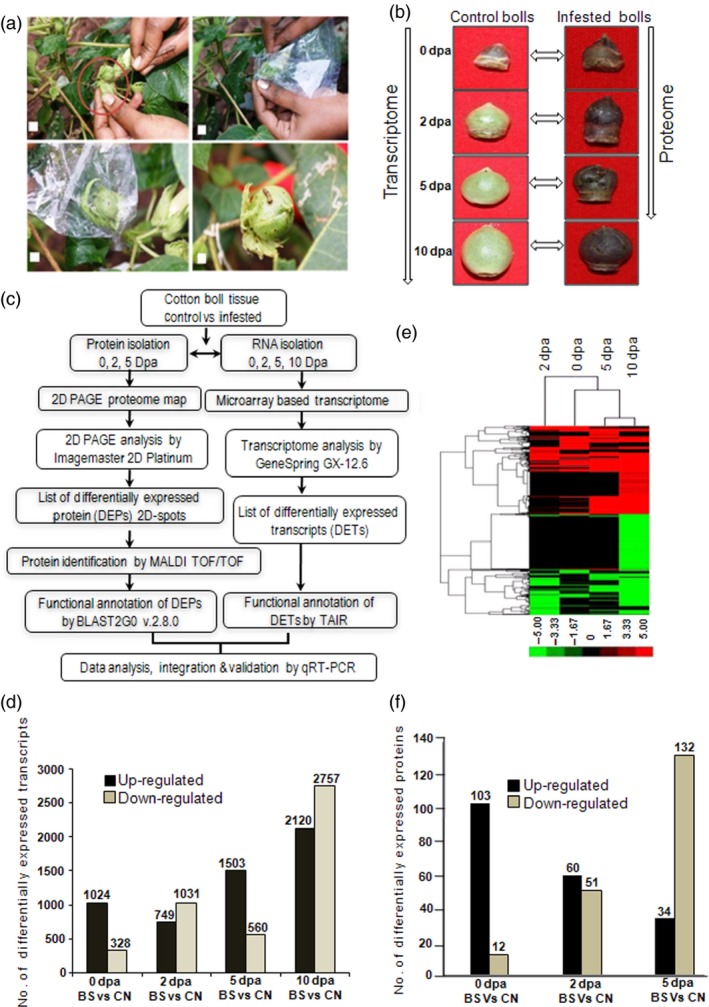
Bollworm infested biotic stress induction in cotton bolls, G. hirsutum L. cv. Bikaneri Narma. (a) Method of biotic stress induction in cotton bolls under field conditions. (b) Boll developmental stages of control (CN) and bollworm infected tissues (BS) used in the current study (0, 2, 5 and 10 dpa/days post anthesis). (c) Schematic overview of proteome and transcriptome data generation and analyses workflow. (d) Number of differentially expressed transcripts (DETs) during boll development stages under BS as compared to their respective stages of CN. (e) Cluster analysis showing the differentially expressed transcripts related to biotic stress. (f) Number of differentially expressed proteins (DEPs) during boll development stages under BS as compared to their respective stages of CN.

**Figure 2:**
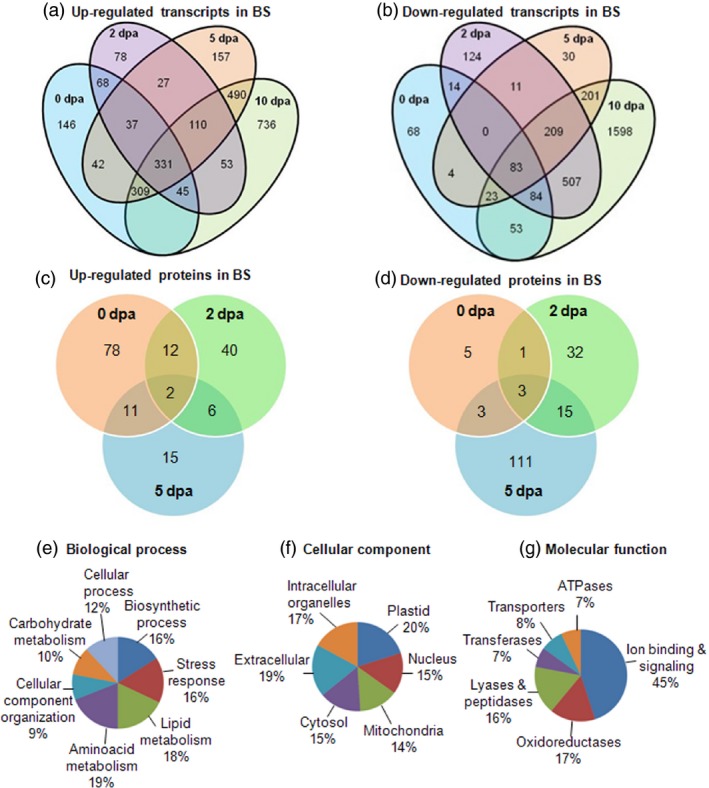
Diagrammatic view of the up‐ and down‐regulated transcripts (a, b) and proteins (c, d) among the boll developmental stages under bollworm infestation in comparison with their respective controls. Gene Ontology‐based annotation and classification of the differentially expressed proteins into the (e) biological process, (f) cellular component and (g) molecular function categories.

Cotton plants were grown in the field conditions following common agronomic practices. Only bolls that were infected by cotton bollworm (*H. armigera*) insect larvae were used for further analysis (Figure 1a,b). To study the effect of insect stress in developing boll tissue, microarray‐based transcriptome profiling and two‐dimensional gel electrophoresis (2D PAGE) followed by MALDI TOF/TOF‐based proteome analyses were carried out at different developmental stages (Figure 1b,c). Labelled mRNA was hybridized to Affymetrix cotton GeneChip Genome array. Identified transcripts with a false discovery rate (FDR) adjusted *P* value ≤0.01 and fold change ≥3 were considered as differentially expressed transcripts (DETs) (Figure 1d; Table S1). In total, 8694 transcripts comprising 39% of the total transcripts present on the cotton GeneChip showed differential expression under bollworm infestation. Transcripts were annotated using the Arabidopsis TAIR protein database version 10 through BLASTX with *E* value cut‐off ≤e‐10. Identification of transcripts related to TFs, phytohormones and signal transduction were attained using Arabidopsis transcription factor and Arabidopsis hormone databases, respectively, as mentioned in Materials and methods. Classification of DETs under different functional categories was attained using MIPS functional catalogue. Gene expression patterns in response to bollworm infestation were classified using hierarchical clustering (Figure 1e). Differentially expressed transcripts showing consistent up‐ and down‐regulation among boll developmental stages are tabulated (Figure 2a,b; Tables S4 and S5).

**Figure 3:**
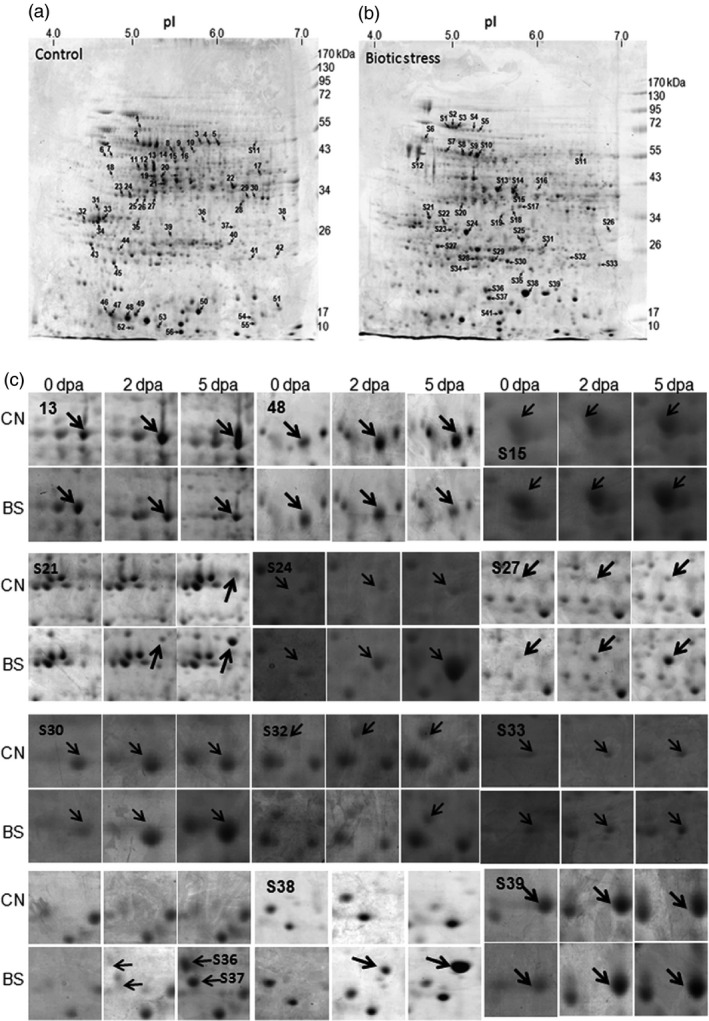
Representative Coomassie stained 2D PAGE proteome profile of Control, CN (a) and Boll worm infested, BS (b) cotton bolls. Annotated 2D spots corresponding to the differentially expressed proteins identified using MALDI TOF/TOF. Detailed list of identified proteins are tabulated in Table S14. (c) 2D Spot profile of representative proteins showing differential expression under bollworm infestation in comparison with their respective control bolls during boll developmental stages (0, 2, 5 dpa). 2D PAGE proteome profile of infested and control bolls during developmental stages are presented in Figure S1.

Two‐dimensional gel electrophoresis (2D PAGE)‐based proteome analysis of bollworm‐infested (biotic stress‐induced BS) and noninfested (control, CN) cotton bolls showed an average of 393 reproducibly detected protein spots across developmental stages (0, 2 and 5 dpa) (Figures 3a–c and S1a–c). At least two independent replicate gels were generated per sample (Control, CN vs bollworm infested, BS). Only those spots that were reproducibly detected were further considered for comparative analysis. Quantification of the protein spots was attained using the per cent volume criterion that corresponds to the expression level of the detected spot regions. Protein spots showing fold change of ±1.5 with a *P* value <0.5 as mentioned in Materials and methods were considered as differentially expressed spots. Comparative analysis revealed that around 35% of the detected spots (137 spots) were differentially expressed (±1.5‐fold), and among them, 98 spots were identified using MALDI TOF/TOF (Table S14). Further Gene Ontology (GO)‐based annotation and functional classification of the DEPs under various categories were attained using BLAST2GO platform version 2.7 (Figure 2e,f).

Comparative analysis of the transcriptome and proteome data sets highlighted 37 overlapping unique accessions that were quantified at both the transcript and protein levels (Table S15). Among them, only 10 genes (accessions) were found to have similar expression pattern in at least one of the developmental stages that were analysed in this study. Such poor corelation among the transcriptome and proteome data sets could either be attributed to the post‐transcriptional regulation of the identified genes or be the experimental limitations associated with the transcript and protein turnover measurements.

### Bollworm infestation induces early and consistent response in developing cotton bolls

**Figure 4:**
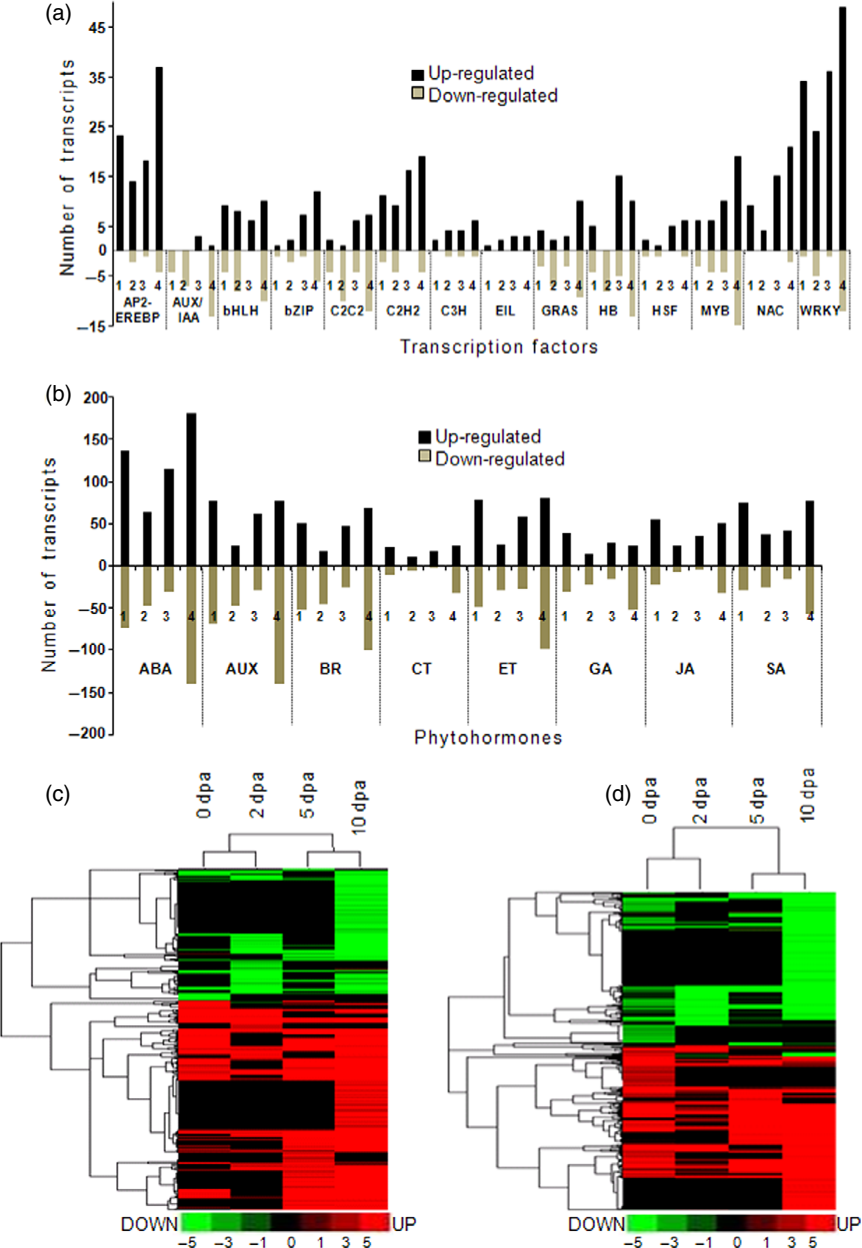
Differentially expressed transcription factors (a) and phytohormone (b) under bollworm infestation as compared to their respective control. Numbers 1–4 represents different stages; (1)—0 dpa, (2)—2 dpa, (3)—5 dpa and (4)—10 dpa. Cluster analysis performed using log_2_‐transformed fold change values showing the differentially expressed transcripts related to transcription factors (c) and phytohormones (d) at boll developmental stages. Putative transcription factors and phytohormones at each stage are presented in Tables S2 and S3.

**Figure 5:**
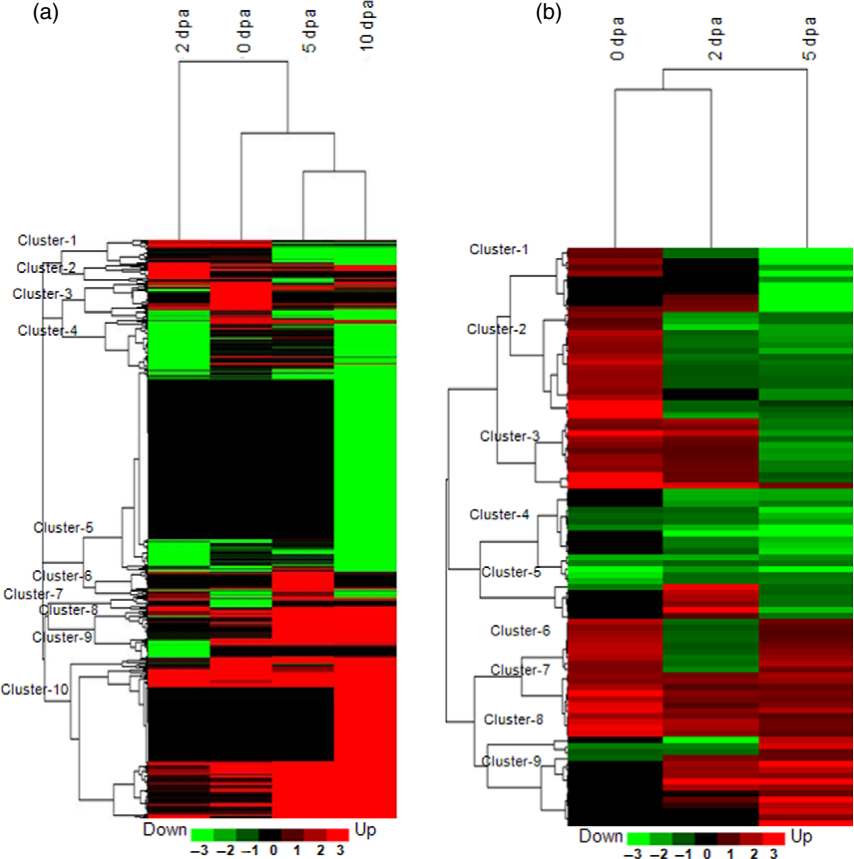
Heat map view of the cluster analysis depicting the expression pattern of differentially expressed transcripts (DETs) (a) and differentially expressed proteins (DEPs) (b). Hierarchical cluster analyses of DETs (fold change ±3) and DEPs (fold change ±1.5) under biotic stress as compared to their respective control samples during fibre development stages (0, 2, 5 and 10 dpa). List of Affymetrix cotton probe set IDs, and fold change for transcripts present in each cluster are presented in Table S16. List of Spot IDs, protein accessions and fold change for proteins are presented in Table S17. The hierarchical clustering was performed using complete linkage method with Euclidean distance based on fold change data compared to control samples using Cluster 3.0.

Analysis of the transcriptome and proteome profile of the bolls that were subjected for only 8 h of bollworm infestation (0 dpa) showed 1352 (15.55%) DETs and 115 (36.37%) DEPs (Figure 1d,f). Majority of those transcripts (75.73%) and proteins (89.5%) were exclusively up‐regulated in biotic stress‐induced bolls (BS) at 0 dpa (Figure 1d,f and 4). Cluster analysis of the DETs and DEPs revealed that the gene expression pattern gradually changed upon boll development (Figure 5a,b). Briefly, 42.07% of 2‐dpa, 72.85% of 5‐dpa, 43.46% of 10‐dpa transcripts were up‐regulated, while 54.05% of 2 dpa and only 25.75% of 5 dpa proteins were up‐regulated in BS‐induced bolls (Figure 1d,f). Analysis of the transcriptome data sets revealed an increase in the number of up‐regulated transcripts at 5 and 10 dpa (Figure 1d). However, such pattern was not observed at protein levels as the proteome profile showed a steady decline towards development (0–5 dpa, Figure 1f). In support of such drastic decline in transcript and protein populations, infested bolls showed compromised growth in terms of size accompanied by infection‐related symptoms such as browning and rotting (Figure 1b). The discrepancies among transcriptome and proteome pattern on the one hand and growth of infested boll on the other suggest that the plant system's continuous effort to encounter pest attack at transcript level is somehow not been translated to the protein level. Nevertheless, the above‐mentioned discrepancies can also be attributed to the limitations associated with transcript and protein quantitation procedures.

### Defence signalling involves major reprogramming of metabolic and biosynthetic pathways

Gene Ontology‐based functional annotation revealed that 62% of the DEPs were enzymes involved in regulating metabolic processes such as carbohydrate (10%), amino acid (19%) and lipid (18%) metabolisms (Figure 2e). Cellular component‐based classification showed that DEPs were distributed among plastid (20%), mitochondria (14%), nucleus (15%), extracellular (19%) and cytosolic (15%) localizations (Figure 2f). Molecular function‐based classification showed that majority of the DEPs were involved in signalling (45%) and redox regulation (17%) (Figure 2g). Significantly enriched pathways corresponding to up‐ and down‐regulated transcripts are presented in Table 1. Hierarchical clustering clearly showed that there were certain population of transcripts and proteins that were down‐regulated upon development and certain others that were consistently up‐regulated across the developmental stages (Figure 5a,b). To explain the reprogramming pattern, we have focussed on the key metabolic pathways that showed major differences in terms of their expression exclusively during biotic stress conditions. Differentially expressed transcripts and DEPs related to trehalose, raffinose, malate, starch and cell wall metabolism majorly accounted for the carbohydrate metabolism (Table S9). Briefly, up‐regulated transcripts related to trehalose phosphate synthase (TPS) and trehalose phosphatase (TPP) that are involved in trehalose biosynthesis (Tables 2 and S9) were identified in this study (Wingler, 2002). Two unique isoforms of galactinol synthases (GolS) involved in galactinol synthesis were found to be consistently up‐regulated in our data set (Tables 2, S4, and S9). Further, our study also showed the up‐regulated transcripts of malate synthase (MS) and down‐regulated malate dehydrogenase (MDH) transcripts and proteins (Tables S9 and S14). Malate synthase is involved in the synthesis of malate, whereas MDH catalyses the oxidation of malate to pyruvate and CO_2_. The above‐mentioned pattern in turn correlates with stress‐responsive accumulation of trehalose, raffinose and malate in the infested bolls. In plants, synthesis and degradation of nonstructural carbohydrates (NSCs) such as starch plays a major role in the regulation of carbon source availability during growth and unfavourable conditions (Sulpice *et al*., 2009). Data curation revealed the down‐regulated transcripts involved in starch biosynthesis such as starch synthase, ADP‐glucose pyrophosphorylase (AGPase) and up‐regulated enzymes of starch degradation pathway such as starch excess 1 and starch binding domain‐containing glycoside hydrolase (Yano *et al*., 2005) (Table 2, S1). Further analysis showed the down‐regulated group of carbohydrate active enzymes (CAZymes) that catalyse cell wall metabolism‐related processes such as loosening, elongation, grafting and maturation (Table S7). Analysis of nitrogen, amino acid and protein metabolism genes revealed that glutamine metabolism was up‐regulated and ubiquitin cascade‐related genes were differentially regulated. Pathway curation revealed that glutamine synthase isoform 1 (GS1), glutamate dehydrogenase (GDH) and nitrate reductase (NiR) involved in nitrogen metabolism were found to be up‐regulated (Tables S4 and S14). In case of ubiquitin cascade, we observed that ubiquitin ligase (E3) was up‐regulated, whereas ubiquitin conjugating enzyme (E2) was down‐regulated in BS condition at transcript and protein levels (Tables S4, S11, and S14). This in turn suggests the onset of rate limiting pattern or controlled proteolysis through ubiquitin‐mediated protein degradation during stress. In addition to the metabolic enzymes, we also observed up‐regulated members of both sugar and amino acid transporters throughout the developmental stages suggesting active transportation processes (Table S12). Further, our study also revealed that fatty acid and lipid metabolism‐related genes were differentially regulated (Table S10). Pathway mapping and curation suggested stimulation as well as repression of different lipid precursor pathways. Briefly, majority of the up‐regulated genes were involved in glycolipid and phospholipid metabolism including enzymes related to α‐linolenic acid metabolism that ultimately lead to JA biosynthesis (Tables S10 and S14). On the other hand, we observed that down‐regulated genes such as 3‐oxo‐5‐alpha‐steroid 4‐dehydrogenase (DET 2) and 24‐sterol C‐methyltransferase (SMT2‐2) were involved in sterol biosynthesis. Sterols are membrane lipids that serve as precursor molecules for brassinosteroid (BR) biosynthesis (Table S10).

**Table 1:** Significantly regulated metabolic pathways under biotic stress

Bin no.	Probe set ID	Cotton gene accessions	Arabidopsis ortholog ID	Gene function	Boll developmental stages (log 2‐transformed fold change values)			
					0 dpa	2 dpa	5 dpa	10 dpa
Cell wall								
10.1.2	ghiaffx.19354.1.s1_s_at	DW488498.1	AT4G10960	UGE5 (UDP‐d‐glucose/UDP‐d‐galactose 4‐epimerase 5)	35.22	3.95	5.46	10.6
10.1.30.3	ghiaffx.59913.1.a1_s_at	DW501774.1	AT3G01640	GHMP kinase family protein	6.05	3.93	3.41	10.81
10.2	ghi.3263.1.a1_at	DT467895		CSLE6—Cellulose synthase‐like family E	67.36	14.35	3.28	23.75
10.5.1.1	ghi.2198.1.s1_s_at	DT550268	AT5G55730	FLA1 (Fasciclin‐like arabinogalactan 1)	−46.39	−4.13	−15.9	−3.85
10.6.3	gra.2038.1.a1_s_at	CO124831	AT1G49320	BURP domain‐containing protein	−13.63	−3.18	−5.12	−3.59
10.6.3	ghi.7933.1.s1_at	AF410458.1	AT3G07970	QRT2 (QUARTET 2); polygalacturonase	8.16	33.95	3.78	198.2
10.6.3	ghi.4648.2.a1_s_at	AY464057.1	AT1G49320	BURP domain‐containing protein	−11.98	−3.17	−4.78	−3.57
10.7	ghi.10493.1.s1_s_at	DT466412	AT5G57560	TCH4 (Touch 4)	12.36	7.98	4.52	7.42
10.7	ghi.6188.1.a1_at	CO493635	AT2G39700	ATEXPA4 (Arabidopsis thaliana Expansin A4)	−76.94	−3.49	−4.22	−3.12
10.7	graaffx.27319.1.s1_s_at	CO089724	AT2G36870	Xyloglucan: xyloglucosyl transferase	−20.46	−7.63	−9.77	−3.68
10.8.1	ghi.3458.1.a1_at	DT465599	AT1G02810	Pectinesterase family protein	15.41	12.31	14.09	11.89
Secondary metabolism								
16.1.5	ghi.3337.1.a1_at	DT467161		DT467161; DB_XREF = GH_CHX19P12.r	20.89	12.27	10.09	20.18
16.2	ghi.5889.2.s1_s_at	Ghi.5889	AT3G26040	Transferase family protein	−32.25	−4.95	−9.9	−7.21
16.2	graaffx.15820.1.s1_s_at	CO123702	AT5G23940	EMB3009 (embryo defective 3009)	−15.72	−3.68	−5.99	−7.07
16.7	ghi.9734.1.s1_at	AW186914	AT5G55340	Long‐chain alcohol O‐fatty‐acyltransferase family protein	−20.5	−4.1	−4.88	−11.25
16.1	ghiaffx.2668.1.a1_at	DT463855	AT5G05390	LAC12 (laccase 12)	117.15	28.42	6.33	23.95
16.1	ghi.3427.1.a1_s_at	DT465993	AT5G09360	LAC14 (laccase 14)	13.57	21.96	8.45	7.89
16.1	ghi.9266.1.a1_at	DT468530	AT4G39830	l‐ascorbate oxidase	17.25	4.82	5.36	5.68
Hormone signalling								
17.2.3	gra.275.1.s1_s_at	CO090219	AT1G60710	ATB2; oxidoreductase	25.46	5.4	4.28	13.3
17.1.3	ghi.2169.1.a1_at	DT468931	AT5G13200	GRAM domain‐containing protein/ABA‐responsive protein‐related	130.07	11.99	10.05	19.8
17.5.1	graaffx.20159.1.a1_s_at	CO110779	AT1G05010	ACO4, 1‐aminocyclopropane‐1‐carboxylate oxidase	29.6	4.69	18.42	13.57
17.5.1	ghiaffx.4691.1.a1_at	DW517948.1	AT1G78440	ATGA2OX1 (gibberellin 2‐oxidase 1)	14.1	26.98	14.1	18.17
17.5.1	ghi.6898.1.s1_at	CA992714	AT5G24530	DMR6 (Downy mildew‐resistant 6)	20.92	3.44	3.17	6
17.5.1.1	ghi.5451.1.s1_at	DQ122174.1	AT4G11280	ACS6 (1‐Aminocyclopropane‐1‐carboxylic acid (ACC) synthase 6)	17.73	29.71	5.56	44.56
17.5.2	ghiaffx.39399.1.s1_at	DW499761.1	AT5G47220	ERF2 (ethylene‐responsive element binding factor 2)	18.53	9.83	6.81	12.43
17.8.1	ghiaffx.43038.1.s1_at	DW497938.1	AT1G19640	JMT (Jasmonic acid carboxyl methyltransferase)	10.54	36.36	18.85	14.13
17.7.1.2	ghi.1739.4.s1_x_at	DT464580	AT1G17420	LOX3; lipoxygenase	22.56	7.96	14.44	7.55
17.7.1.2	gra.38.1.s1_s_at	CO123081	AT3G45140	LOX2 (Lipoxygenase 2)	11.39	5.32	15.23	5.91
17.7.2	ghi.10327.1.s1_s_at	CO123081	AT1G19180	JAZ1 (Jasmonate zim‐domain protein 1)	65.2	4.87	5.13	14.28
Respiratory function								
20.1.1	ghi.9151.2.s1_at	DT049218	AT5G51060	RHD2 (Root hair defective 2)	11.00	10.03	8.79	4.52
Defence genes								
20.1.7	ghiaffx.8053.1.a1_at	DW518810.1	AT1G64160	Disease resistance‐responsive family protein/Dirigent family protein	10.77	77.58	5.85	7.53
20.1.7.6.1	ghiaffx.61299.1.s1_at	DW508705.1	AT1G17860	Trypsin and protease inhibitor family protein/Kunitz family protein	45.7	24.03	14.33	153.28
Heat‐shock proteins								
20.2.1	graaffx.28942.1.s1_s_at	CO085044	AT4G36990	HSF4 (Heat‐shock factor 4)	13.22	3.58	4.07	5.71
20.2.1	ghiaffx.11624.1.s1_s_at	DW505128.1	AT4G27670	HSP21 (Heat‐shock protein 21)	3.18	3.84	3.79	10.39
20.2.1	ghi.10640.1.s1_s_at	DN780720	AT3G12580	HSP70 (Heat‐shock protein 70)	5.7	3.17	6.44	14.67
20.2.1	ghi.9308.1.s1_at	DT527234	AT5G59720	HSP18.2 (Heat‐shock protein 18.2)	4.47	5.41	7.22	15.12
Redox regulators								
21.3	ghi.8085.1.s1_at	AF329368.1	AT2G16060	AHB1 (Arabidopsis haemoglobin 1)	26.19	12.11	20.59	4.35
21.3	garaffx.24390.1.s1_s_at	BF273948	AT3G10520	AHB2 (Arabidopsis haemoglobin 2)	−8.71	−10.68	−4.49	−3.05
21.4	ghiaffx.62078.1.s1_at	DW517172.1	AT1G28480	GRX480; Electron carrier/Protein disulphide oxidoreductase	4.03	4.43	3	7.31
26.12	ghi.7950.1.s1_at	AY366083.1	AT5G06720	Peroxidase	61.08	400.1	3.34	16.95
26.9	ghiaffx.2752.1.s1_at	CA993163	AT2G29420	ATGSTU7 (Arabidopsis thaliana glutathione s‐transferase tau 7)	91.46	8.58	12.83	9.66
Transcription factors								
27.3.3	ghi.10443.1.s1_at	DT049130	AT1G19210	AP2 domain‐containing transcription factor	8.38	14.35	19.22	3.42
27.3.3	gbaaffx.196.1.a1_s_at	AY572462.1	AT3G16770	ATEBP (Ethylene‐responsive element binding protein)	45.61	58.16	10.37	21.92
27.3.3	ghiaffx.25508.1.s1_at	DW496030.1	AT5G50080	ERF110, DNA binding/Transcription factor	183.63	28.72	49.8	15.07
27.3.35	ghiaffx.4349.1.a1_s_at	DW509077.1	AT3G62420	ATBZIP53 (Basic region/Leucine zipper motif 53)	6.93	4.37	3.72	6.81
27.3.35	ghi.5267.1.a1_at	DT046626	AT4G34590	GBF6 (G‐Box binding factor 6)	−144	−7.71	−23	−5.66
27.3.25	ghiaffx.8694.1.s1_a_at	DW499451.1	AT4G05100	AtMYB74 (Myb domain protein 74)	233.09	28.85	14.35	77.79
27.3.25	ghi.10620.1.s1_at	DT465545	AT4G37260	MYB73 (Myb domain protein 73)	12.84	10.67	3.75	8.2
27.3.32	gra.1530.2.s1_s_at	CO125258	AT2G03340	WRKY3; Transcription factor	12.89	3.59	3.8	9.18
27.3.32	ghi.9240.1.s1_s_at	DT468893	AT2G38470	WRKY33; Transcription factor	14.81	3.33	5.33	5.47
27.3.32	ghi.9192.1.s1_s_at	DT468825	AT1G80840	WRKY40; Transcription factor	8.88	11.55	4.95	4.86
27.3.32	ghiaffx.30199.1.s1_at	DW506814.1	AT2G47260	WRKY23; Transcription factor	43.52	30.64	3.93	22.82
27.3.32	ghiaffx.6177.1.s1_at	DW505740.1	AT5G13080	WRKY75; Transcription factor	3.91	4.33	3.28	4.62
27.3.32	ghi.9182.3.s1_at	DT461494	AT1G62300	WRKY6; Transcription factor	59.11	8.2	5.34	14.9
Protein degradation								
29.5.1	gra.1032.1.s1_s_at	CO123218	AT5G67360	ARA12; Serine‐type endopeptidase	−7.45	−3.52	−6.69	−3.79
29.5.1	ghiaffx.53444.1.a1_s_at	DW229324.1	AT2G05920	Subtilase family protein	−47.35	−3.35	−17.5	−3.71
29.5.4	ghi.7891.1.s1_s_at	DT462224	AT1G03220	Extracellular dermal glycoprotein	322.82	34.58	9.5	54.08
29.5.4	ghi.10117.2.a1_s_at	DT462947	AT1G44130	Nucellin protein	7.56	7.06	4.6	3.93
29.5.7	ghi.6869.1.a1_at	CA992801	AT1G70170	MMP (Matrix metalloproteinase)	37.2	3.78	3.15	13.9
29.5.7	ghi.6849.1.a1_at	CA992877	AT5G15250	FTSH6 (FTSH protease 6)	42.13	4.35	4.19	31.63
29.5.9	ghiaffx.12228.1.s1_at	DW512183.1	AT2G46620	AAA type ATPase family protein	18.34	3.48	3.78	12.66
29.5.11.4.2	ghi.6875.2.a1_at	DT466966	AT3G02840	Immediate–early fungal elicitor family protein	43.34	7.19	21.82	14.16
29.5.11.4.2	ghiaffx.13045.1.s1_at	DW226240.1	AT1G29340	PUB17 (Plant U‐box 17)	16.02	3.63	5.23	7.17
29.5.11.4.2	ghi.6875.1.s1_at	CA992786	AT1G66160	U‐box domain‐containing protein	26.45	3.69	9.54	9.73
29.5.11.4.2	ghiaffx.15780.1.a1_s_at	DW224570.1	AT4G03510	RMA1; protein binding/Ubiquitin‐protein ligase/Zinc ion binding	13.96	3.93	6.46	9.12
29.5.11.4.2	ghi.2766.2.s1_s_at	DT550860	AT3G05200	ATL6; protein binding/Zinc ion binding	6	5.23	3.45	4.26
29.5.11.4.2	ghi.4550.1.a1_at	DT051102	AT5G42200	Zinc finger (C3HC4‐type RING finger) family protein	15.82	7.93	3.08	21.12
29.5.11.4.2	ghi.965.2.s1_at	DT467116	AT2G37150	Zinc finger (C3HC4‐type RING finger) family protein	4.48	3.46	3.25	5.48
29.5.11.4.3.2	ghi.8366.1.s1_s_at	DT462696	AT1G30200	F‐box family protein	37.35	15.69	15.9	16.16
Signalling								
30.1.1	ghi.10316.1.s1_s_at	DT051688	AT2G35150	EXL1 (Exordium‐like 1)	−83.47	−3.57	−8.79	−6.26
30.1.1	gra.1243.1.s1_s_at	CO123292	AT2G17230	EXL5 (Exordium‐like 5)	−66.14	−9.46	−11.3	−3.47
30.1.1	ghiaffx.45244.1.s1_x_at	DT051989	AT5G51550	EXL3 (Exordium‐like 3)	−29.46	−5.73	−15.9	−5.58
30.2.3	ghi.8869.1.s1_s_at	DT457129	AT2G26730	Leucine‐rich repeat transmembrane protein kinase	−18.69	−3.85	−13.9	−6.78
30.2.11	ghi.5385.1.s1_s_at	DT047980	AT5G66330	Leucine‐rich repeat family protein	−24.57	−7.2	−26.1	−3.48
30.2.17	ghiaffx.15325.1.s1_at	DT466359	AT2G38090	MYB family transcription factor	4.07	12.7	11.78	4.74
30.2.17	graaffx.26883.1.a1_s_at	CO090919	AT1G56130	Leucine‐rich repeat family protein/Protein kinase family protein	14.95	9.38	4.26	4.78
30.2.20	ghiaffx.24594.1.s1_at	DT466937	AT1G18390	ATP binding/protein tyrosine kinase	7.52	7.5	4.9	4.09
30.2.23	ghi.9233.1.a1_at	DT465825	AT1G11050	Protein kinase family protein	43.61	9.59	6.17	8.26
30.3	ghiaffx.25338.1.a1_at	DW515811.1	AT5G37780	CAM1 (Calmodulin 1)	15.32	8.21	10.95	4.95
30.3	ghiaffx.12687.1.a1_at	DW489633.1	AT2G41860	CPK14; ATP binding/Protein tyrosine kinase	4.24	4.07	7.55	3.22
30.3	ghiaffx.40480.2.s1_at	DW497946.1	AT5G24270	SOS3 (Salt overly sensitive 3)	8.35	4.03	4.02	6.62
30.3	ghi.4855.1.a1_at	CA993640	AT5G49480	ATCP1 (Ca^2+^‐binding protein 1)	13.48	3.85	3.21	7.31
30.3	ghi.1114.1.s1_s_at	DN817396	AT5G39670	Calcium‐binding EF‐hand family protein	49	14.56	4.36	9.21
30.3	ghi.3763.1.a1_s_at	DT461952	AT3G63380	Calcium‐transporting ATPase, plasma membrane‐type	7.63	8.69	3.3	4.96
30.3	ghiaffx.42473.1.s1_at	DW508297.1	AT5G13460	IQD11 (IQ domain 11); Calmodulin–binding	−17.58	−3.82	−12.7	−7.35
30.3	ghi.2692.1.s1_at	DT465311	AT2G15760	Calmodulin‐binding protein	13.32	6.84	3.88	4.16
30.3	ghiaffx.6766.1.s1_at	DW504268.1	AT5G54490	PBP1 (Pinoid‐binding protein 1)	13.38	34.38	19.25	11.08
30.6	ghi.6088.2.s1_at	DT467539	AT3G45640	ATMPK3 (Arabidopsis thaliana mitogen‐activated protein kinase 3)	54.54	9.53	3.27	21.63
30.8	ghiaffx.23436.1.s1_x_at	DR455118	AT5G67070	RALFL34 (Ralf‐like 34)	−18.79	−3.06	−6.19	−7.91

**Table 2:** Expression pattern and biological role of crucial genes identified in the bollworm‐infested cotton bolls through transcriptome and proteome approaches

S no.	Accession	Transcript ID/Protein ID	Expression pattern (fold change values)				Biological significance
			0 dpa	2 dpa	5 dpa	10 dpa	
Carbohydrate metabolism—Stabilizers & osmoprotectants							
01	AY628139.1	Trehalose 6‐phosphate synthase (TPS)	4.79	–	10.26	9.43	Trehalose biosynthesis. Stabilization of macromolecular structures
02	DT462221		3.57	4.32	10.51	13.89	
03	DT465672		18.25	–	58.87	82.78	
04	DT464172	Trehalose‐6‐phosphate phosphatase (TPP)	–	–	3.12	25.58	
05	DW238688.1	Galactinol synthase 1 (GolS)	23.33	4.21	8.38	5.78	Raffinose biosynthesis. Raffinose–osmoprotectants
06	DW227458.1	Galactinol synthase 2 (GolS)	3.98	29.09	7.78	84.01	
07	X52305.1	Malate synthase	5.77	–	134.42	355.63	Malate biosynthesis
08	DW232960.1	Malate dehydrogenase	−3.54	−3.22	–	25.44	
	gi¦11133373^a^	Malate dehydrogenase	−7.356	−1.868	−5.188	–	
09	DT455780	Starch synthase III	−4.55	–	–	−5.64	Starch synthesis. Starch‐carbon source during unfavourable conditions
10	DN760794	Putative starch synthase	–	–	–	−14.69	
11	CO121872	Starch Excess 1 (SEX‐1)	–	–	−3.27	–	
12	DW501538.1	Starch binding Glycoside hydrolase	–	–	4.24	6.70	
Amino acid metabolism—Stress‐specific protein isoforms, nitrogen mobilization							
13	gi¦211906462^a^ CA993194	Glutamine synthase	–	1.76	1.61	−5.280	Biotic stress‐specific protein isoform GS1 (Figure 3c, spot 15)
14	gi¦121334^a^	Glutamine synthetase PR‐1 (GS1)	1.53	1.54	1.72	–	
15	gi¦99698^a^	Glutamate‐ammonia ligase	2.1002	1.3649	1.2754	–	
16	DT462524	Glutamate dehydrogenase (GDH)	3.72	3.22	9.32	8.51	
17	CO085887	Nitrate reductase (NiR)	6.91	10.53	18.72	70.65	
Lipid metabolism–Phytohormone biosynthesis							
18	DT047194	Allene Oxide synthase (AOS)	3.57	5.11	4.13	–	α‐Linolenic acid metabolism. Jasmonic acid biosynthesis
20	gi¦40643247^a^	Allene oxide cyclase (AOC)	8.10	2.59	1.36	–	
21	DQ116446.1	3‐oxo‐5‐alpha‐steroid 4‐dehydrogenase (DET2)	–	–	−3.83	−9.09	Brassinosteroid biosynthesis
22	AJ513325		–	–	−5.14	−5.23	
23	DT564348	Sterol methyl transferase (SMT‐2)	−6.84	−14.19	−3.02	−14.51	
24	CO122079	Zeaxanthin epoxidase	−3.21	–	–	–	ABA biosynthesis
25	DQ122174.1	Aminocyclopropane‐1‐carboxylate synthase (ACC)	29.71	5.56	44.57	17.73	Ethylene biosynthesis
Retrograde signalling components and salicylic acid suppressors							
26	DT462103	WRKY 40	124.03	61.51	1.29	29.29	Re Retrograde signalling (Mitochondria)
27	DT466107	Alternative Oxidase (AOX)	33.53	3.66	19.82	94.72	
28	CA993875	Pentatricopeptide repeat containing protein (GUN1)	14.25	63.25	21.79	59.99	Retrograde signalling (Chloroplast)
29	DT468893	WRKY 33	3.33	5.33	5.48	14.81	Suppressors of Salicylic Acid
30	DW506256.1	Ethylene insensitive 3 (EIN 3)	3.15	4.24	7.72	13.69	
31	DW241764.1	Enhanced Disease Resistance 1(EDR1)	–	–	–	3.49	
32	CO085044	Heat‐shock transcription factor (HSf‐1)	3.59	4.07	5.71	13.23	Regulator of GolS
Defence molecules							
33	DT554033	Chitinase	40.76	21.29	8.05	49.51	Defence, (Figure 3c, spot 27)
34	gi¦1729760^a^	Chitinase	–	1.73	2.28	–	
35	DN780414	Osmotin	75.69	–	32.98	382.36	
36	gi¦595836886^a^	Osmotin	4.47	−1.1568	1.58	–	
37	CF932178	Pathogenesis‐related protein 4 (PR 4)	11.19	3.87	5.12	29.86	
38	gi¦10505374^a^	Pathogenesis‐related protein 10 (PR 10)	Protein spot detected only under biotic stress condition				Figure 3c, spots 36, 37

Fold change values with −ve sign indicate down‐regulation.

–: No fold change value was determined.

a Protein IDs and expression values obtained from proteome analysis.

### Repression of chloroplast, mitochondrial metabolism and cellular growth is evident during stress

**Figure 6:**
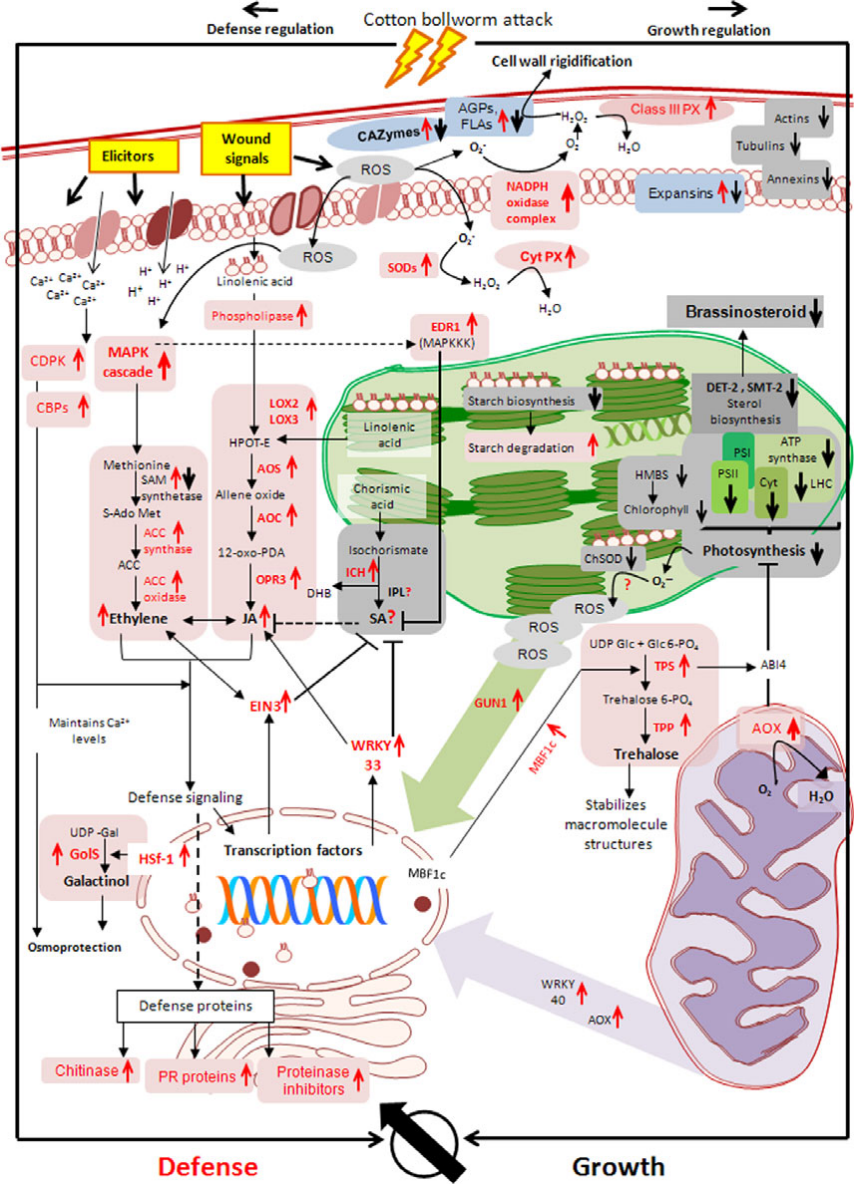
Putative model depicting the regulation of molecular events in cotton bolls subjected to bollworm infestation. Genes related to the redox regulation—peroxidase (PX), super oxide dismutase (SODs), metabolic process—trehalose phosphate synthase (TPS), trehalose phosphate phosphatase (TPP), galactinol synthase (GolS), signalling cascades—calcium‐dependent protein kinase (CDPK), calcium‐binding proteins (CBPs), mitogen‐activated protein kinase (MAPK), enhanced disease resistance 1 (EDR1), phytohormone synthesis—lipoxygenase (LOX2, LOX3), allene oxide synthase (AOS), allene oxide cyclase (AOC), 12‐oxo‐phytodienoic acid reductase (OPR3), S‐adenosylmethionine synthetase (SAM), 1‐aminocyclopropane‐1‐carboxylate (ACC) synthase/oxidase, isochorismatase hydralase (ICH), isochorismate pyruvate lyase (IPL), 3‐oxo‐5‐alpha‐steroid 4‐dehydrogenase (DET 2), 24‐sterol C‐methyltransferase (SMT2‐2), transcription factors—ethylene insensitive 3(EIN3), heat‐shock transcription factor (HSf‐1), multiprotein bridging factor 1c (MBF1c), retrograde signalling—alternative oxidase (AOX), pentatricopeptide repeat containing protein (GUN1), defence—pathogenesis‐related (PR) protein, photosynthesis—photosystem (PS I, PS II), cytochrome complex (Cyt), light harvesting complex (LHC), hydroxy methyl bilane synthase (HMBS) and growth—carbohydrate active enzymes (CAZymes) are annotated along with their expression pattern. Upward pointing arrow indicates up‐regulation and downward pointing arrow indicates down‐regulation of respective genes. The overall pattern suggests the selective regulation of signalling cascades favouring defence over growth in bollworm‐infested cotton bolls.

In this study, the redundant expression pattern observed in majority of the metabolic pathways included both up‐ and down‐regulated genes. However, in case of photosynthesis, most of the DETs and proteins were found to be majorly down‐regulated throughout the developmental stages. Briefly, genes related to photosystem I, II, ATP synthase, light harvesting complex, chlorophyll binding proteins, etc. were consistently down‐regulated under BS condition (Tables S8 and S14). Also genes encoding enzymes involved in carbon fixation including ribulose‐1, 5‐bisphosphosphate carboxylase/oxygenase (RuBisCO) and members of tricarboxylic acid cycle were found to be down‐regulated (Table S8). However, we observed transcripts related to pentatricopeptide repeat containing protein (GUN1) were up‐regulated (Tables S2 and S11; Figure 6). Further, transcripts related to mitochondria such as ATP synthase (mitochondrial), phosphate transporter, mitochondria‐associated membrane glycoprotein (MAM33), etc. were found to be down‐regulated (Tables S9 and S12), while transcripts related to mitochondrial alternative oxidase (AOX) involved in alternative respiratory pathway were found to be consistently up‐regulated (Tables 2 and S1) (Vanlerberghe and McIntosh, 1997). In addition, cellular growth‐related genes such as cytoskeleton proteins, annexins, profilins and expansins were found to be down‐regulated upon development (Tables S7 and S14). Analysis of cell cycle and DNA replication‐related genes revealed that members of cyclin‐dependent protein kinase family, cell division‐related proteins, histones, DNA replication factors, etc. were down‐regulated in BS‐induced bolls (Table S7).

### Calcium and redox signalling pathways are stimulated to regulate host response

Calcium (Ca^2+^) is often quoted as the second important messenger that activates signalling cascades in response to various stimuli including biotic stress (Sanders *et al*., 2002). Genes encoding Ca^2+^/calmodulin‐binding proteins, calcineurin B‐like proteins, Ca^2+^ binding EF‐hand family proteins, Ca^2+^‐dependent ATPases were found to be up‐regulated under BS conditions (Tables 1 and S14). Protein kinase cascades account for the major contributors of induced immunity in plants. In this study, we observed the up‐regulation of two major stress‐activated protein kinases such as calcium‐dependent protein kinases and mitogen‐activated protein kinases. Also, other kinases such as SNF1(sucrose nonfermenting)‐related protein kinase, calcineurin B‐like interacting protein kinase‐6 (CIPK6), ankyrin protein kinases were found to be significantly up‐regulated in BS condition. The above‐mentioned Ca^2+^ signalling molecules can be broadly classified into nonenzymatic sensor proteins and enzymatic proteins. These proteins together constitute a network that not only maintains the intracellular calcium levels but are also involved in other signalling events including protein activation. Among the Ca^2+^‐dependent enzymes, majority were observed to be kinases involved in phosphorylation reaction that determines the activity of substrate protein molecules. So we further analysed the data sets for kinase substrates and the pathways regulated by these kinases during stress. Interestingly, we observed CDPK‐activated protein substrates such as NADPH oxidases and oxidoreductases that were up‐regulated during later stages of development (10dpa) (Dubiella *et al*., 2013). Manual curation of data sets revealed that members of peroxidases, superoxide dismutases (SODs), glutathione S‐transferases (GSTs), etc. were found to be differentially regulated at transcript and protein levels (Tables S13 and S14). These enzymes are actively involved in the ROS metabolism leading to detoxification reactions and activation of downstream signalling cascades.

### Transcription factor analysis revealed components of stress, retrograde signalling and suppressors of SA

Analysis of the transcriptome data revealed 1048 differentially expressed TFs accounting for 12% of the DETs under BS conditions (Table S2). Further, around 8.1% (705) of the up‐regulated and 3.94% (343) of the down‐regulated transcripts correspond to transcription factor families (TFs). Stress‐responsive TFs belonging to WRKY, AP2‐EREBP, NAC, bHLH, MYB, C2H2 and ethylene insensitive three families were found to be up‐regulated (Figure 4a). Further, a number of development‐related TFs belonging to AUX/IAA, C2C2, GRAS and HB families were down‐regulated during boll developmental stages (Figure 4a). Among the TFs, WRKY family accounted for about 13% of differentially expressed TFs in all of the analysed developmental stages (0–10 dpa). Following WRKY, AP2‐EREBP, NAC and MYB TFs were found to be relatively more in the transcriptome data set. Among the stress‐related TFs, AP2‐EREBP plays a central role in the abscisic acid (ABA)‐dependent stress signalling pathways. Literature survey and manual curation of the TFs composition and regulation pattern revealed that the TFs such as WRKY 40, NAC along with AOXs as mentioned elsewhere constitutively account for the components of mitochondrial retrograde signalling pathways (Sophia *et al*., 2013; Van Aken *et al*., 2013). Further analysis revealed the up‐regulated members of WRKY33 and EIN3 that act as suppressors of SA.

### Bollworm attack induces synthesis of synergistically regulated phytohormones

Phytohormone‐related DETs constituted for about 64.5% of 0 dpa and <25% of 2, 5 and 10 dpa. Classification and annotation of phytohormone‐related transcripts showed that around 24% of them were related to ABA followed by auxin (AUX/IAA), ethylene (ET), BR, SA, gibberellic acid (GA), JA and cytokinin. Manual curation revealed that transcripts corresponding to zeaxanthin epoxidase that catalyses the first step of ABA biosynthesis were found to be down‐regulated (Table S3) (Marin *et al*., 1996). Transcripts corresponding to tryptophan aminotransferase (TAA1), aldehyde dehydrogenase of indole 3‐pyruvic acid pathway (IPA) and cytochrome P450 enzyme‐CYP79B2 of the indole‐3‐acetaldoxime pathway (IAOX) were found to be down‐regulated. In addition, flavin monooxygenase (YUC), tryptophan decarboxylase of the indole‐3‐acetamide (IAM) pathway, transcripts related to nitrilases were found to be up‐regulated (Table S3). The above‐mentioned pathways (IPA, IAOX and IAM) ultimately lead to auxin synthesis in plants. The aforementioned expression pattern suggests that auxin biosynthesis is partially inhibited during bollworm attack. Further, transcripts related to 1‐aminocyclopropane‐1‐carboxylic acid (ACC) synthase and ACC oxidase involved in ethylene biosynthesis were found to be consistently up‐regulated (Table S4). Transcripts related to sterol biosynthesis were found to be down‐regulated (Tables S3 and S10). Sterols serve as precursor for BR synthesis. In addition, transcripts related to 3‐oxo‐5‐alpha‐steroid 4‐dehydrogenase and BR biosynthetic protein DWARF1 involved in BR biosynthesis also were found to be down‐regulated. Isochorismatase hydrolase (ISH) was the only SA biosynthetic pathway‐related enzyme that was found to be up‐regulated in our data set (Table S1). However, pathway annotation revealed that ISH catalyses the conversion of isochorismic acid to 2, 3‐dihydroxybenzoic acid (DHB) and this reaction does not necessarily lead to SA biosynthesis in plants (Figure 6). Interestingly, most of the crucial enzymes involved in JA biosynthesis including phospholipase A, lipoxygenase, allene oxide synthase (AOS), allene oxide cyclase (AOC) and acyl‐coA oxidase were found to be up‐regulated throughout the developmental stages in BS‐induced bolls at transcript and protein levels (Tables S10 and S14). Expression pattern of the hormonal biosynthesis‐related genes suggests that biotic stress has induced JA and ET that are reported to act in a cooperative fashion (Figure 6). Likewise, ABA being the major contributor for DETs showed down‐regulated pattern leading to its suppression during biotic stress. Down‐regulation of BR and partial stimulation of auxin provides clues about the rate limiting pattern for allowing growth during stress.

### Bona fide defence molecules and pathways are stimulated in response to biotic stress

The ultimate output of metabolic reprogramming, transcription factor regulation, ROS, Ca^2+^ signalling, hormonal biosynthesis, etc. leads to stimulation and synthesis of defence molecules and processes. In our data set, defence‐related proteins such as the members of pathogenesis‐related protein family (PR4, PR10, osmotin and thaumatin), chitinase, beta‐glucanase and proteinase inhibitors were found to be up‐regulated at transcript and protein levels (Figure 3c; Tables 2, S4, and S14). In addition, transcripts related to polyamine biosynthesis pathway such as SAM decarboxylase, spermidine synthase, arginine decarboxylase also were found to be up‐regulated. Polyamines are cited as phytohormone like molecules that accumulate in response to stress and they also play major role in defence (Gill and Tuteja, 2010; Hussain *et al*., 2011; Waie and Rajam, 2003). In addition, ROS regulating enzymes such as peroxidases (cytosolic and extracellular), SODs, NADPH oxidase and oxidative stress‐specific GSTs were found to be up‐regulated (Table S13).

### Comparative analysis highlights concordant and discordant members of transcript‐protein pairs

Comparative analysis revealed 37 unique accessions that were commonly identified in both the transcriptome and proteome approaches (Table S15). Gene Ontology‐based annotation and classification of these genes revealed that majority of them were involved in metabolic, cellular and biosynthetic processes including amino acid, nucleotide and osmolyte metabolism, stress and defence response and phytohormone biosynthesis (Figure S3a–c). Manual curation of the data sets showed two distinct groups of transcript and protein pairs such as the genes with concordant (similar) expression patterns and the genes with discordant (dissimilar) expression patterns at transcript and protein levels (Table S15). Concordant members included genes such as chaperonin, actins, phosphoglycerate kinase, gibberellin oxidase, MDH and SODs that were found to be down‐regulated, while defence and stress response‐specific genes such as chitinase, PR protein, protease inhibitor and carbonic anhydrase were found to be up‐regulated across developmental stages. Discordant members included ATP synthases, RuBisCO, glutamine synthase, proteasome subunits, etc. that showed poor corelation in their transcript and protein expression patterns.

### Validation of microarray and proteome data by qRT‐PCR analysis

To validate the data, semi‐quantitative real‐time PCR (qRT‐PCR) analysis was performed on 42 selected differentially expressed genes (32 up‐regulated and 10 down‐regulated) during boll developmental stages under cotton bollworm infestation (Figure S2). The results showed that the expression patterns of transcripts and proteins observed through microarray and proteome analyses were in parallel with those obtained by qRT‐PCR (Figure S2).

## Discussion

In the current study, transcriptomic approach has resulted in the identification of relatively more number of differentially expressed genes as compared to the proteomic approach. Nevertheless, the expression pattern of crucial phytohormone biosynthesis genes including S‐adenosylmethionine synthase and AOC, cytoskeleton proteins such as annexin isoforms and actins, cytosolic ascorbate peroxidase involved in redox regulation, signalling and stress‐specific response genes such as calcium‐binding protein and GS1 were exclusively identified at the protein levels. Transcriptomic approach revealed a vast set of TFs which are otherwise difficult to be identified at protein levels. The concordant pattern observed among the transcript‐protein pairs such as chitinase, PR proteins, carbonic anhydrase and protease inhibitors adds significance and direct evidence for active defence signalling during bollworm infestation in developing cotton bolls. Also, discordance observed among transcript‐protein pairs might reflect true biological discordance that could be attributed to post‐transcriptional regulations, protein/transcript stability, miRNAs, etc. and this needs to be investigated further. On the whole, our data suggest that employing two complementary approaches have increased the overall coverage of the differentially expressed genes that in turn has aided in filling crucial gaps in the above‐mentioned processes.

## Host–pest interactions induce synthesis of additional metabolic resources ensuring survival

Transcriptomic and proteomic data obtained in this study revealed major changes in the carbohydrate metabolisms such as the significantly up‐regulated genes encoding TPS, TPP and GolS involved in trehalose and raffinose biosynthesis (Table 2) and down‐regulated genes involved in cell wall metabolisms (Table S7; Figure 6). Trehalose is a nonreducing disaccharide that plays a major role in the stabilization of proteins and molecular structures during stress (Garg *et al*., 2002). Likewise, raffinose belongs to a family of oligosaccharides that accumulates during stress and acts as osmoprotectants (Unda *et al*., 2012). Both trehalose and raffinose family of saccharides are referred to as compatible solutes responding to stress conditions in plants (Zhou *et al*., 2014). In addition to that, carbohydrate active and associated proteins like CAZymes, AGPs and FLAs were found to be differentially regulated in our data set (Table S7). AGPs are a heterogeneous class of abundant proteoglycans localized in both cell wall and cytosolic regions (Kumar *et al*., 2013). Reactive oxygen species molecules (H_2_O_2_) released during stress conditions, cross‐link the AGPs to the cell wall leading to rigidity and thereby render protection against pest and pathogen invasion. The rigidity caused in the cell wall matrix also acts as a negative regulator of cell growth (Cleland and Karlsnes, 1967; Gille *et al*., 2009; Sadava and Chrispeels, 1973).

Analysis of the nitrogen and amino acid metabolism highlighted additional clues on BS regulation. Briefly, GS1 and glutamate‐ammonia ligase were found to be consistently up‐regulated throughout the developmental stages (Table S14). In plants, glutamine (Gln) serves as the primary source for inorganic nitrogen, N (${\text{NO}}_{3}^{-}$ and ${\text{NH}}_{4}^{+}$) that gets subsequently utilized for biosynthesis of major amino acids like Glu, Asp and Asn. Among the genes involved in nitrogen/amino acid metabolism, glutamine synthetase (GS), glutamate synthase, GDH and NiR play primary roles in the assimilation of ${\text{NH}}_{4}^{+}$. Two isoforms of GS are reported in plants among which GS1 is induced and the other isoform, GS2 is suppressed during pathogen attack (Pageau *et al*., 2006). Interestingly, our study revealed the consistent up‐regulation of GS1, GDH and NiR under BS condition. Among the above mentioned, GS2 and NiR are involved in primary nitrogen assimilation, whereas GS1 and GDH are involved in organic nitrogen remobilization (Pageau *et al*., 2006). In addition, GS1 and GDH are also cited as senescence‐related markers in plants (Pageau *et al*., 2006). Expression pattern observed in the current study in turn suggests that nitrogen remobilization and senescence leading to stress regulation is active, whereas signals related to primary nitrogen assimilation leading to growth are not evidenced.

Lipid metabolism serves as one of the major contributor for energy, membrane biogenesis, signalling molecules, etc. Our study revealed a bias in the stimulation of certain lipid metabolic pathways. Briefly, linolenic acid metabolic enzymes were up‐regulated, whereas sterol biosynthesis genes were down‐regulated. Linolenic acid and sterols are lipid molecules colocalized within the plastid and thylakoid membranes (Schwertner and Biale, 1973). Our data showed that the factors related to linolenic acid metabolism leading to biosynthesis of stress‐responsive JA were induced, while the genes related to sterol biosynthesis that leads to growth‐related Br synthesis were down‐regulated. Interestingly, cellular component‐based annotation of the above‐mentioned pathways and processes revealed that growth and defence‐related molecules are colocalized within the same compartment such as chloroplast. However, under biotic stress, only defence‐related factors are positively regulated leaving behind the growth‐related factors (Figure 6). Such a switch over in the regulation of lipid metabolism at subcellular level further ensures resistance during bollworm attack.

### Diverse pathway regulation and association delineates bollworm infestation‐specific signalling pattern

In addition to above‐mentioned metabolic pathways, signalling molecules such as calcium, redox regulators, phytohormones, TFs and protein kinases that play independent role through diverse pathways were found to be differentially regulated in our data set (Figures S3 and S4). Briefly, up‐regulated members of Calcium (Ca^2+^) binding proteins and Ca^2+^ transporting ATPases are involved in maintaining cytosolic calcium levels during stress conditions. Redox regulators and oxidative stress regulators such as SODs, GSTs, peroxidases, NADPH oxidases, respiratory burst oxidase homologs (Rbohs), DHARs, etc. were found to be temporally regulated in our data set (Figures 6 and S4; Table S13). Among them, NADPH oxidases, Rbohs and extracellular peroxidases are the major regulators of the primary apoplastic oxidative burst during insect attack (Torres, 2010). These enzymes catalyse reactions leading to ROS release which further stimulates downstream enzymes like SODs and GSTs localized at other cellular components. Superoxide dismutases and GSTs catalyse detoxification reactions, while PCBRs are involved in antioxidant synthesis ultimately protecting cells from oxidative stress (Niculaes *et al*., 2014). Temporal expression of the ROS scavengers indicates a compromised pattern executed by cotton bolls in response to bollworm attack. Phytohormones are secondary signals that often regulate development and stress conditions in plants. Our study showed the positive regulation of stress‐related hormones such JA and ethylene accompanied by repression or down‐regulation of growth‐related Auxin, BR (Figure 5). Interestingly, we did not find genes related to SA biosynthesis which is also a biotic stress‐specific hormone; however, positively regulated suppressors for SA synthesis such as EIN3 and WRKY 33 have been evidenced in the current study (Table 2; Figure 6). Such observations in turn suggest that bollworm attack favours synergistic JA–ethylene synthesis over the antagonistic SA. Further, our study also revealed the up‐regulation of nuclear encoded plastid and mitochondrial components including TFs and enzymes. Among them, WRKY TF family is often linked with biotic stress response and pathogen‐associated molecular pattern (PAMP) (Eulgem and Somssich, 2007; Rushton *et al*., 1996). In addition, WRKY TFs also regulate the expression of nuclear encoded mitochondrial proteins (Van Aken *et al*., 2013). For example; up‐regulated members of WRKY40 identified in the current study are known to regulate the expression of AOX enzyme during stress conditions (Ivanova *et al*., 2014). These factors along with ROS molecules together constitute the plastid and mitochondrial signalling pathway components that regulates nuclear gene expression related to cell cycle and growth. Such pattern further reveals the retrograde trend operational during biotic stress conditions (Figure 6).

### Bollworm infestation induces major reallocation of metabolic resources favouring defence over growth

In response to insect pest attack, the host plant initiates several layers of defence including PAMP‐triggered immunity (PTIs), effector‐triggered immunity (Dangl and Jones, 2001). Following stress perception, a series of signal transduction events that include metabolic pathway regulation, defence molecule synthesis, etc. are stimulated to encounter the external attack (Figure S3). The above‐mentioned coordinated events suggest an energy intensive mechanism that needs to be executed in order to exert the defence response. Knowledge gained through the current study highlights that the source for such additional energy could be attained by suppressing growth locally (boll tissue growth). In short, we observed a hierarchy of factors and processes that specifically suppresses growth‐related events and stimulates defence‐related processes. Expression trend of carbohydrate, amino acid and lipid metabolism also reveals the synthesis of stress response‐related molecules such as trehalose, raffinose, linolenic acid and suppression of growth‐related factors such as cell wall elongation enzymes, and sterols. Suppression of fundamental processes such as photosynthesis and cell cycle not only retards further growth but also regulates the amount of ROS released by them and also preserves considerable amount of energy that could be channelized for defence signalling. Likewise, defence response such as lignifications, cross‐linking of proteoglycans to cell wall matrix offers cell wall rigidification on the one hand and negatively regulates cellular expansion on the other. All these factors together suggest a major reallocation of metabolic resources favouring defence over growth through selective regulation of specific pathways and processes during bollworm attack.

## Conclusion

The present study delineates boll‐specific endogenous defence mechanisms adapted by cotton plants under bollworm attack. The vast number of coordinated events including stimulations and repressions of major biological processes ultimately suggest major reallocation of metabolic resources that favours defence over growth in developing cotton bolls. Taking such insights into account, strategies targeting stimulation of multiple phytohormones and better sustainment of defence as well as growth signals could aid in developing resistant varieties against insect pest.

## Materials and methods

### Plant material and biotic stress treatment

Cotton (*Gossypium hirsutum* cv. Bikaneri Narma) plants were grown at Agricultural Research Station, Dharwad farm, Dharwad, during 2012–2013 Kharif seasons following recommended agronomic practices. Two separate plots of the same genotype were maintained with a space of 90 cm between rows and 20 cm between plants. The plots were covered with nylon nets to protect from any external pest incidence. Plot designated as control (no infection from any class of insects including bollworms) and plot designated as infested (infested with *H. armigera*) were protected in early stage (45 days after sowing) from incidence of sucking pests by spraying recommended insecticides. During peak flowering stage (65–85 days after sowing), 2nd–3rd instar larvae of *H. armigera*, raised on bendi (*Abelmoschus esculentus* L. Syn. *Hibiscus esculentus*) fruits in the Entomology laboratory maintained at 25 °C in 65%–70% relative humidity on a 14/10‐h light/dark cycle, were released on buds of cotton on the day of pollination. The buds with larva were covered using paper bag with proper aeration to prevent larvae movement from the bud. Such a set‐up ensured maximum damage of bolls by larva. The cotton bolls used as control(s) were also covered with paper bags with pores in order to prevent predation by insect pests and to ensure similar microenvironment as that of biotic stress‐induced bolls. Samples were collected after 8 h of infection and labelled as 0 dpa, likewise samples collected after 2 and 5 days of insect infestation were labelled as 2 and 5 dpa, respectively. After 5 days of infestation, the insect was removed; the bolls were collected after 10 days of further growth and were labelled as 10 dpa. Harvested cotton boll samples were frozen immediately in liquid nitrogen and stored at −70 °C until further use.

### Total RNA isolation and Microarray experiments

Infected bolls (complete) from 0 and 2 dpa and only infested portion of 5 and 10 dpa boll samples along with their respective controls were used for RNA extraction. In order to minimize plant to plant and mode of infection variations, boll samples were collected and pooled from five independent plants and considered as one biological replicate. Total RNA isolation, analysis and quality check were performed as previously described. Affymetrix Cotton GeneChip Genome array (Affymetrix, Santa Clara, California) having 23 977 probe sets representing 21 854 cotton transcripts was used for transcriptome analysis (http://www.affymetrix.com/catalog/131430/AFFY/Cotton-Genome-Array#1_1). Three biological replicates were maintained to test the reproducibility and quality of the chip hybridization. Microarray hybridization, staining and washing procedures were carried out as described in the Affymetrix protocols with minor modifications (Padmalatha *et al*., 2012). The arrays were scanned with a GeneChip scanner 3000.

### GeneChip data processing and analysis

After scanning of each array, DAT, CEL, CHP, XML and JPEG image files were generated using GeneChip Operating Software platform. The CEL files having estimated probe intensity values were analysed with GeneSpring GX‐12.6 software (Agilent Technologies, Santa Clara, California) to get DETs. The robust multiarray average algorithm was used for the back ground correction; quantile normalization and median polished probe set summarization to generate single expression value for each probe set. Normalized expression values were log_2_‐transformed, and differential expression analysis was performed using unpaired *t*‐test. The *P* values were corrected by applying the FDR correction (Benjamini and Hochberg, 2000). Differentially expressed transcripts with FDR corrected *P* value ≤0.01 and fold change ≥3 were included for further data analysis. The hierarchical clustering was performed using complete linkage method with Euclidean distance based on log fold change data compared to control samples using Cluster 3.0 (Eisen *et al*., 1998) to display the expression pattern and tree diagram of DETs. The DETs were annotated using NetAffx annotation data for Cotton GeneChip (http://www.affymetrix.com, release 26).

### Functional annotation of probe sets and pathways

To obtain functional annotation of transcripts, the consensus sequences of probe sets present in the Cotton GeneChip were mapped to the Arabidopsis TAIR protein database version 10 (http://www.arabidopsis.org) by BLASTX with *E* value cut‐off ≤e‐10. To identify the putative TFs and transcripts related to phytohormone biosynthesis and signal transduction pathways, the consensus sequences of all probe sets presented in cotton GeneChip were searched against the Arabidopsis transcription factor database (http://plntfdb.bio.uni-potsdam.de, version 3.0) and Arabidopsis hormone database (http://ahd.cbi.pku.edu.cn, version 2.0), respectively, by BLASTX with *E* value cut‐off ≤e‐10. Differentially expressed transcripts were grouped into functional categories based on MIPS functional catalogue (http://mips.gsf.de/projects/funcat). Further, MapMan software version 3.5.0 (http://gabi.rzpd.de/projects/MapMan/) was used to visualize the expression of differentially regulated cotton transcripts onto metabolic pathways (Usadel *et al*., 2005). The microarray data are deposited in the Gene Expression Omnibus (GEO) database (http://www.ncbi.nlm.nih.gov/geo) at the NCBI under the series accession numbers GSE55511.

### Two‐dimensional gel electrophoresis (2D SDS‐PAGE)‐based proteome analysis

The total protein from cotton bolls was isolated using phenol extraction method. The protein pellets were dissolved in 2D SDS‐PAGE rehydration buffer (7 m urea, 2 m thiourea, 2% CHAPS, 0.5% ampholytes, 40 mm DTT), and an aliquot of protein sample from two independent replicates was subjected to two‐dimensional gel electrophoresis (2D SDS–PAGE) as described previously (Kumar *et al*., 2013), briefly for the first‐dimensional separation, the sample was loaded onto a 13‐cm immobilized pH gradient (IPG) linear (pI 4–7) strips (GE Healthcare Life Sciences, U.S.A), and isoelectric focusing was performed according to manufacturer's instructions. Strips were then equilibrated, and second‐dimensional separation was carried out on 12% SDS–polyacrylamide gel (13 cm, 1.5 mm). Gels were stained with Coomassie blue staining to visualize the protein spots and were stored in 1% acetic acid at 4 °C until further use. Gels were scanned using GE Image scanner III (GE Healthcare Life Sciences, U.S.A) through Labscan software version 6.0.1 and analysed using Imagemaster 2D Platinum software version 6.0.1 (GE Healthcare Life Sciences, U.S.A). Protein spot detection parameters were set as: Smooth: 3, Minimum area: 11 and Saliency: 200. Detected protein spots were manually re‐evaluated to remove artefacts such as dust particles and streaks. Reproducibly detected protein spots were quantified using the per cent volume criterion. The relative volume corresponding to the detected spot region was considered to represent the expression level. Protein spots that showed normalized expression values of ±0.6‐, 1.5‐fold (biotic stress/control) were considered for statistical evaluation. To define the significant difference, *P* value <0.05 was set through Student's *t*‐test and one‐way anova. Protein expression values within the above‐mentioned thresholds were considered as differentially expressed.

### Protein identification, annotation and classification from 2D gel spots

Differentially expressed protein spots were subjected to in‐gel tryptic digestion followed by MALDIT TOF‐based identification procedure as previously described (Kumar *et al*., 2013). Peptide MS/MS spectrum processing was achieved through Flexanalysis software version 3 and database search using Biotools software version 3.2. The database search parameters were set as described: fragment masses were searched in three independent databases: they were (i) NCBInr database (06/03/2010) containing 10 551 781 sequences (total) including 290 173 sequences from green plants (Viridiplantae), (ii) *Gossypium raimondii* protein database containing 40 976 sequences downloaded from CottonGen website (ftp://ftp.bioinfo.wsu.edu/species/Gossypium_raimondii/CGP-BGI_G.raimondii_Dgenome/genes/), (iii) *Gossypium arboreum* protein database containing 40 134 sequences downloaded from Cotton Genome Project website (ftp://cotton:cotton321$@public.genomics.org.cn/Ca_all_Version2.GENE.pep.gz) through mascot search engine, taxonomy was set as Viridiplantae, enzyme was set as trypsin, fixed modifications included carbamidomethylation of cysteine, variable modifications included oxidation of methionine, protein mass was unrestricted, missed cleavage was set to 1, MS tolerance of ±100 ppm and MS/MS tolerance of ±/−0.75 da. Only peptides with an individual ion score of >40 (*P *< 0.05) were considered for protein identification. Identified proteins were sequences that were exported into BLAST2GO platform version 2.7 (www.blast2go.com/b2ghome) to attain GO‐based annotation, classification and pathway mapping (Conesa *et al*., 2005).

### Transcriptome and proteome data set integration

Unique protein sequences corresponding to the differentially expressed proteins were subjected to GEO Nucleotide Translated BLAST: tblastn analysis to obtain accessions corresponding to *Gossypium hirsutum* (taxid: 3635) transcripts. The search parameters for tblastn were set as follows: database—GEO; organism: *G. hirsutum* (taxid: 3635). Transcript accessions with E value cut‐off ≤e‐10 and >70% sequence identity were considered as matched sequence.

### The quantitative real‐time PCR (qRT‐PCR) analysis

The qRT‐PCR analysis was performed on selected differentially expressed genes to validate the microarray and proteome expression data. RNA isolation followed by cDNA synthesis, qRT‐PCR analysis and fold change calculations were performed as previously described (Padmalatha *et al*., 2012). The list of primers used in the current study is presented in Table S6. The *GhPP2A1* gene (accession no: DT545658) from *G. hirsutum* was used as reference gene to normalize the expression values (Artico *et al*., 2010).

## Conflict of interest

The authors declare that they have no conflict of interest.

## Supporting information


**Figure S1** 2D‐PAGE profile of control (uninfected) and bollworm infested cotton boll proteome during developmental stages. 2D‐PAGE profiles of total proteins obtained from control and bollworm infested (Biotic stress) cotton bolls: (a) 0 dpa, (b) 2 dpa cotton bolls, (c) 5 dpa cotton bolls. Equal amount of total proteins (500 μg) were loaded onto 13 cm IPG strips, pI 4‐7 and protein samples were resolved using 12% SDS–PAGE gels.
**Figure S2** Validation of microarray and proteome data using qRT‐PCR during boll development stages (0, 2, 5 and 10 dpa) of cotton under biotic stress. *Y*‐axis represents the log 2 fold change values at various stages in the biotic stress as compared to their respective stages in control.
**Figure S3** Gene ontology based classification of commonly identified genes in transcriptome and proteome datasets under biological process (a), cellular component (b) and molecular function (c) categories. Key events in the signal transduction pathway activated in response to biotic stress (d).
**Figure S4** Overview of gene expression changes in developing cotton bolls infested with bollworm.

**Table S1** Differentially expressed transcripts during boll development stages (0, 2, 5 and 10 dpa) under biotic stress as compared to their respective control samples.

**Table S2** List of differentially expressed transcription factors (TFs) during boll developmental stages under bollworm infestation biotic stress.

**Table S3** List of differentially expressed transcripts related to phytohormone biosynthesis and signaling pathways during boll developmental stages response to bollworm infested biotic stress.

**Table S4** Consistently up‐regulated genes in different developmental stages under biotic stress.

**Table S5** Consistently down‐regulated genes in different developmental stages under biotic stress.

**Table S6** List of qRT‐PCR genes and primers used in this study.

**Table S7** Expression pattern of transcripts related to cell wall, cell division and cell growth.

**Table S8** Expression pattern of transcripts related to photosynthesis.

**Table S9** Expression pattern of transcripts related to carbohydrate metabolism.

**Table S10** Expression pattern of transcripts related to fatty acid metabolism.

**Table S11** Expression pattern of transcripts related to protein metabolism.

**Table S12** Expression pattern of transcripts related to transport mechanism.

**Table S13** Expression pattern of transcripts related to oxidative stress.

**Table S14** List of Differentially expressed proteins identified by MALDI TOF/TOF. Fold change values (BS/CN) are tabulated along with the protein identification details.

**Table S15** List of commonly identified genes in the transcriptome and proteome data sets.

**Table S16** Cluster wise list of differentially expressed transcripts included in the Hierarchical Cluster Analysis depicted in Figure 5a.

**Table S17** Cluster wise list of differentially expressed proteins included in the Hierarchical Cluster Analysis depicted in Figure 4b.
