# Supplementary material for: Genome‐wide transcriptomic and proteomic analyses of bollworm‐infested developing cotton bolls revealed the genes and pathways involved in the insect pest defence mechanism
Source: Plant Biotechnol J. 2016 Jan 22;14(6):1438–55. doi: 10.1111/pbi.12508 (PMC5066800; doi:10.1111/pbi.12508)
Supplement: Supplementary file 1 — Figure S1 2D‐PAGE profile of control (uninfected) and bollworm infested cotton boll proteome during developmental stages. 2D‐PAGE profiles of total proteins obtained from control and bollworm infested (Biotic stress) cotton bolls: (a) 0 dpa, (b) 2 dpa cotton bolls, (c) 5 dpa cotton bolls. Equal amount of total proteins (500 μg) were loaded onto 13 cm IPG strips, pI 4‐7 and protein samples were resolved using 12% SDS–PAGE gels. Figure S2 Validation of microarray and proteome data using qRT‐PCR during boll development stages (0, 2, 5 and 10 dpa) of cotton under biotic stress. Y‐axis represents the log 2 fold change values at various stages in the biotic stress as compared to their respective stages in control. Figure S3 Gene ontology based classification of commonly identified genes in transcriptome and proteome datasets under biological process (a), cellular component (b) and molecular function (c) categories. Key events in the signal transduction pathway activated in response to biotic stress (d). Figure S4 Overview of gene expression changes in developing cotton bolls infested with bollworm. [file PBI-14-1438-s001.ppt]

## Slide 1
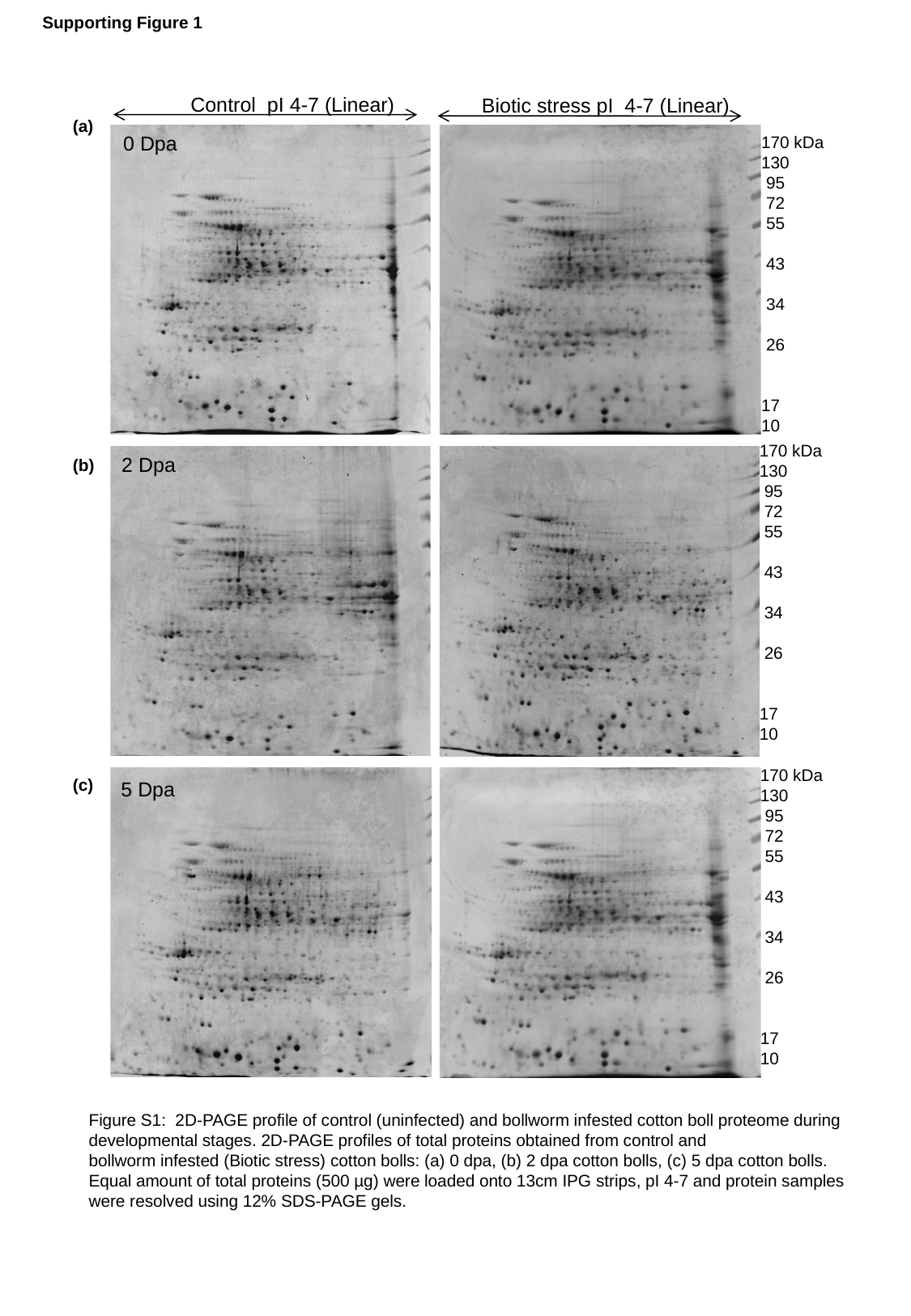

Supporting Figure 1
Control pI 4-7 (Linear)
Biotic stress pI 4-7 (Linear)
0 Dpa
170 kDa
130
 95
 72
 55
 43
 34
 26
17
10
170 kDa
130
 95
 72
 55
 43
 34
 26
17
10
2 Dpa
170 kDa
130
 95
 72
 55
 43
 34
 26
17
10
5 Dpa
(a)
(b)
(c)
Figure S1: 2D-PAGE profile of control (uninfected) and bollworm infested cotton boll proteome during
developmental stages. 2D-PAGE profiles of total proteins obtained from control and
bollworm infested (Biotic stress) cotton bolls: (a) 0 dpa, (b) 2 dpa cotton bolls, (c) 5 dpa cotton bolls.
Equal amount of total proteins (500 µg) were loaded onto 13cm IPG strips, pI 4-7 and protein samples
were resolved using 12% SDS-PAGE gels.

## Slide 2
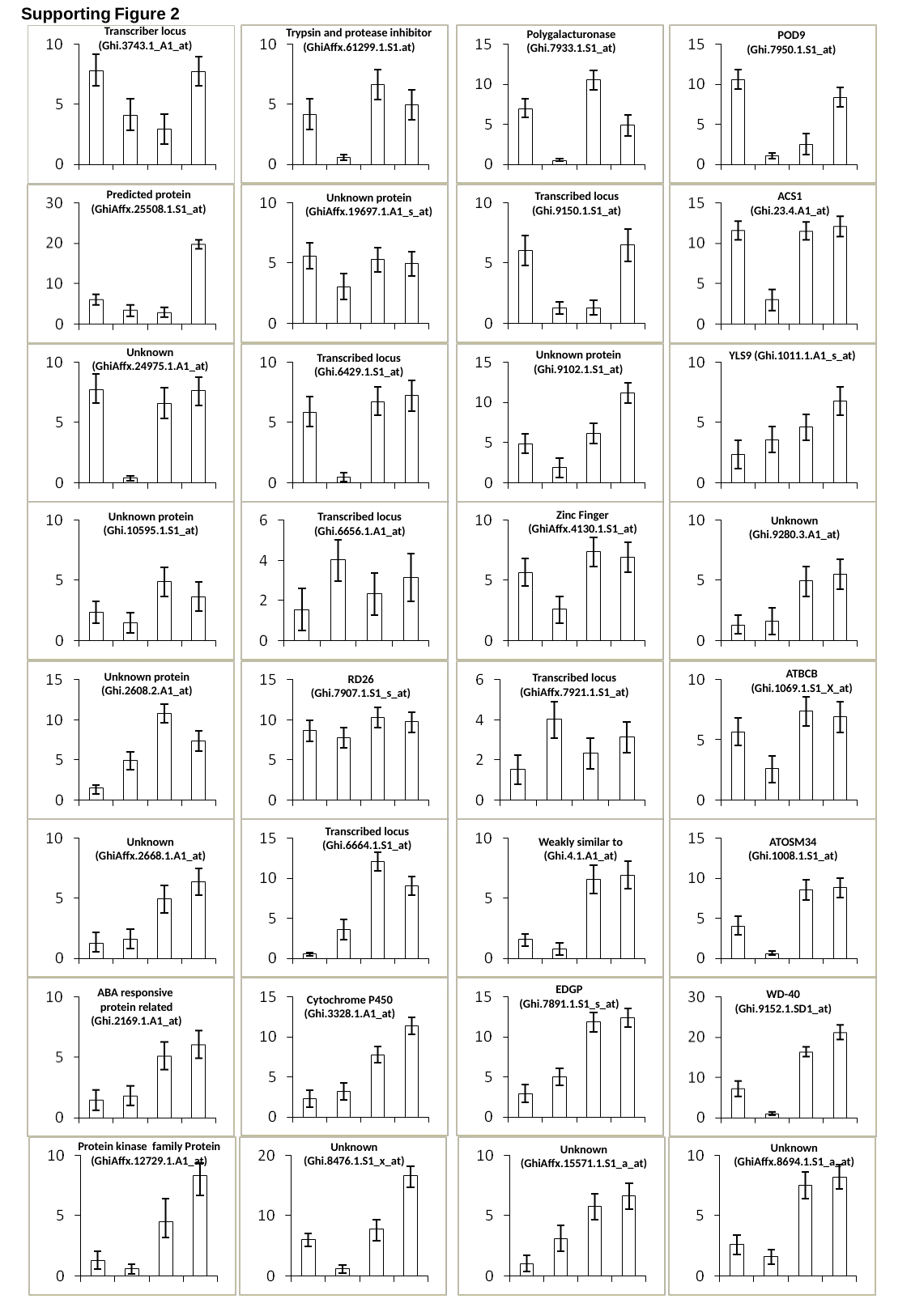

Supporting Figure 2
Transcriber locus (Ghi.3743.1_A1_at)
Trypsin and protease inhibitor (GhiAffx.61299.1.S1.at)
Polygalacturonase (Ghi.7933.1.S1_at)
POD9 (Ghi.7950.1.S1_at)
Predicted protein (GhiAffx.25508.1.S1_at)
Transcribed locus (Ghi.9150.1.S1_at)
ACS1 (Ghi.23.4.A1_at)
Unknown protein (GhiAffx.19697.1.A1_s_at)
Unknown (GhiAffx.24975.1.A1_at)
Unknown protein
(Ghi.9102.1.S1_at)
YLS9 (Ghi.1011.1.A1_s_at)
Transcribed locus (Ghi.6429.1.S1_at)
Zinc Finger (GhiAffx.4130.1.S1_at)
Unknown protein (Ghi.10595.1.S1_at)
Transcribed locus (Ghi.6656.1.A1_at)
Unknown (Ghi.9280.3.A1_at)
ATBCB (Ghi.1069.1.S1_X_at)
Unknown protein (Ghi.2608.2.A1_at)
Transcribed locus (GhiAffx.7921.1.S1_at)
RD26 (Ghi.7907.1.S1_s_at)
Transcribed locus (Ghi.6664.1.S1_at)
Unknown (GhiAffx.2668.1.A1_at)
Weakly similar to (Ghi.4.1.A1_at)
ATOSM34 (Ghi.1008.1.S1_at)
EDGP (Ghi.7891.1.S1_s_at)
ABA responsive
protein related (Ghi.2169.1.A1_at)
WD-40 (Ghi.9152.1.SD1_at)
Cytochrome P450 (Ghi.3328.1.A1_at)
Protein kinase family Protein (GhiAffx.12729.1.A1_at)
Unknown (Ghi.8476.1.S1_x_at)
Unknown (GhiAffx.8694.1.S1_a_at)
Unknown (GhiAffx.15571.1.S1_a_at)

## Slide 3
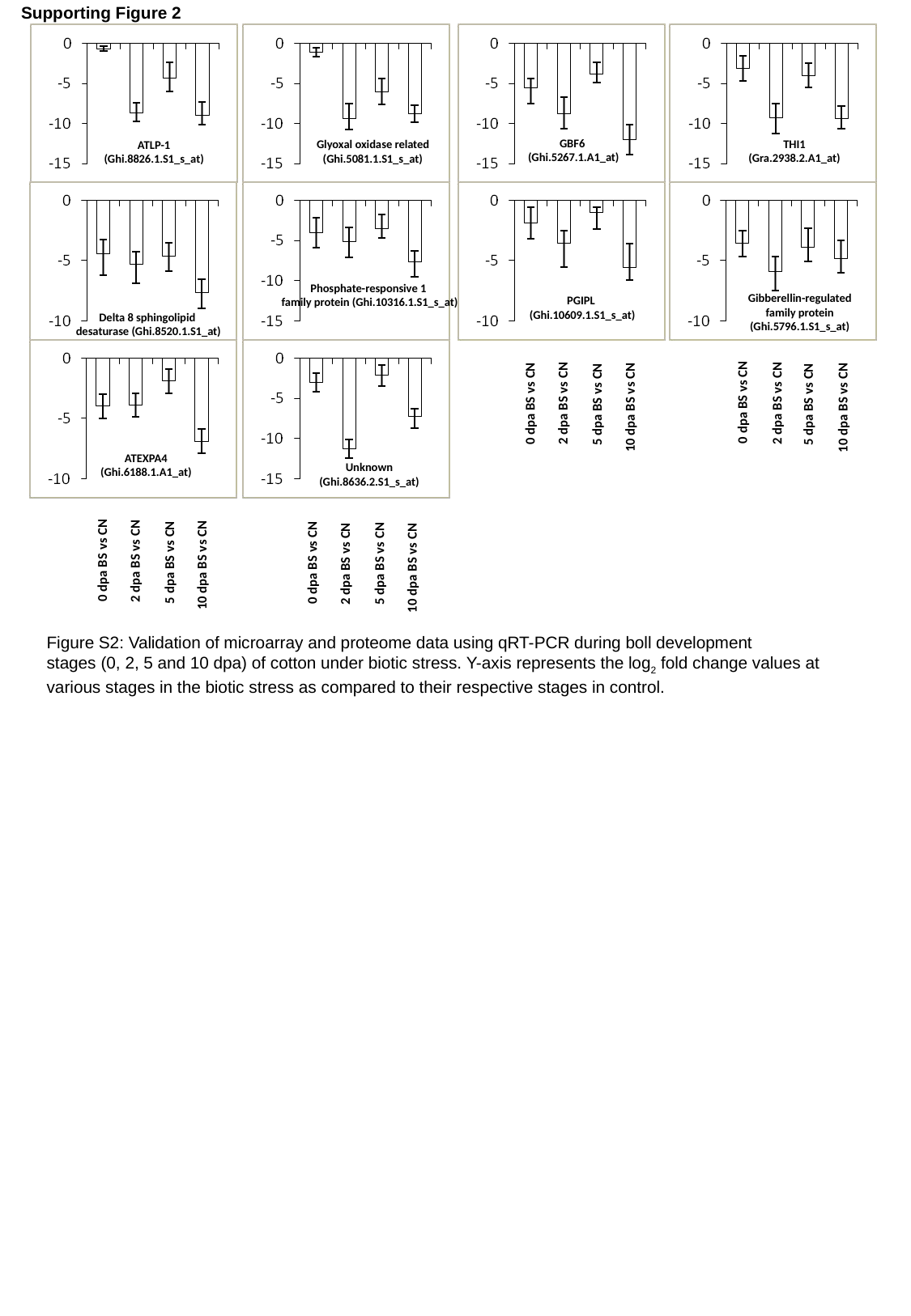

Supporting Figure 2
GBF6
(Ghi.5267.1.A1_at)
THI1 (Gra.2938.2.A1_at)
Glyoxal oxidase related (Ghi.5081.1.S1_s_at)
ATLP-1 (Ghi.8826.1.S1_s_at)
Phosphate-responsive 1
family protein (Ghi.10316.1.S1_s_at)
Gibberellin-regulated family protein (Ghi.5796.1.S1_s_at)
PGIPL
(Ghi.10609.1.S1_s_at)
Delta 8 sphingolipid
desaturase (Ghi.8520.1.S1_at)
0 dpa BS vs CN
2 dpa BS vs CN
2 dpa BS vs CN
0 dpa BS vs CN
5 dpa BS vs CN
5 dpa BS vs CN
10 dpa BS vs CN
10 dpa BS vs CN
ATEXPA4 (Ghi.6188.1.A1_at)
Unknown (Ghi.8636.2.S1_s_at)
0 dpa BS vs CN
2 dpa BS vs CN
5 dpa BS vs CN
0 dpa BS vs CN
5 dpa BS vs CN
2 dpa BS vs CN
10 dpa BS vs CN
10 dpa BS vs CN
Figure S2: Validation of microarray and proteome data using qRT-PCR during boll development
stages (0, 2, 5 and 10 dpa) of cotton under biotic stress. Y-axis represents the log2 fold change values at
various stages in the biotic stress as compared to their respective stages in control.

## Slide 4
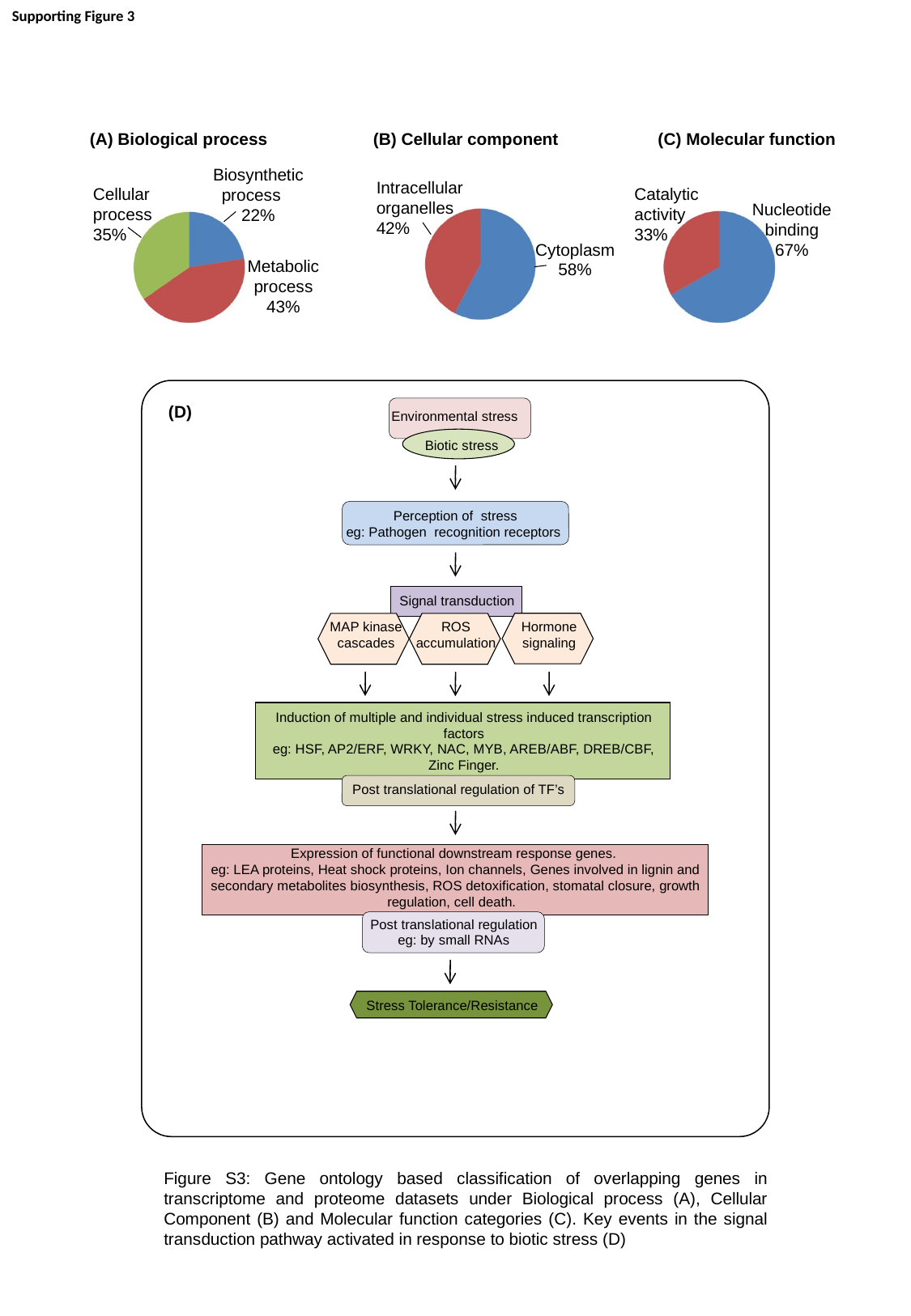

Supporting Figure 3
 (A) Biological process
 (B) Cellular component
 (C) Molecular function
Biosynthetic process 22%
Cellular process
35%
Metabolic process
43%
Intracellular organelles
42%
Cytoplasm
58%
Catalytic activity
33%
Nucleotide binding
67%
 (D)
Environmental stress
Biotic stress
Perception of stress
eg: Pathogen recognition receptors
Signal transduction
MAP kinase cascades
ROS accumulation
Hormone signaling
Induction of multiple and individual stress induced transcription factors
eg: HSF, AP2/ERF, WRKY, NAC, MYB, AREB/ABF, DREB/CBF, Zinc Finger.
Post translational regulation of TF’s
Expression of functional downstream response genes.
eg: LEA proteins, Heat shock proteins, Ion channels, Genes involved in lignin and secondary metabolites biosynthesis, ROS detoxification, stomatal closure, growth regulation, cell death.
Post translational regulation
eg: by small RNAs
Stress Tolerance/Resistance
Figure S3: Gene ontology based classification of overlapping genes in transcriptome and proteome datasets under Biological process (A), Cellular Component (B) and Molecular function categories (C). Key events in the signal transduction pathway activated in response to biotic stress (D)

## Slide 5
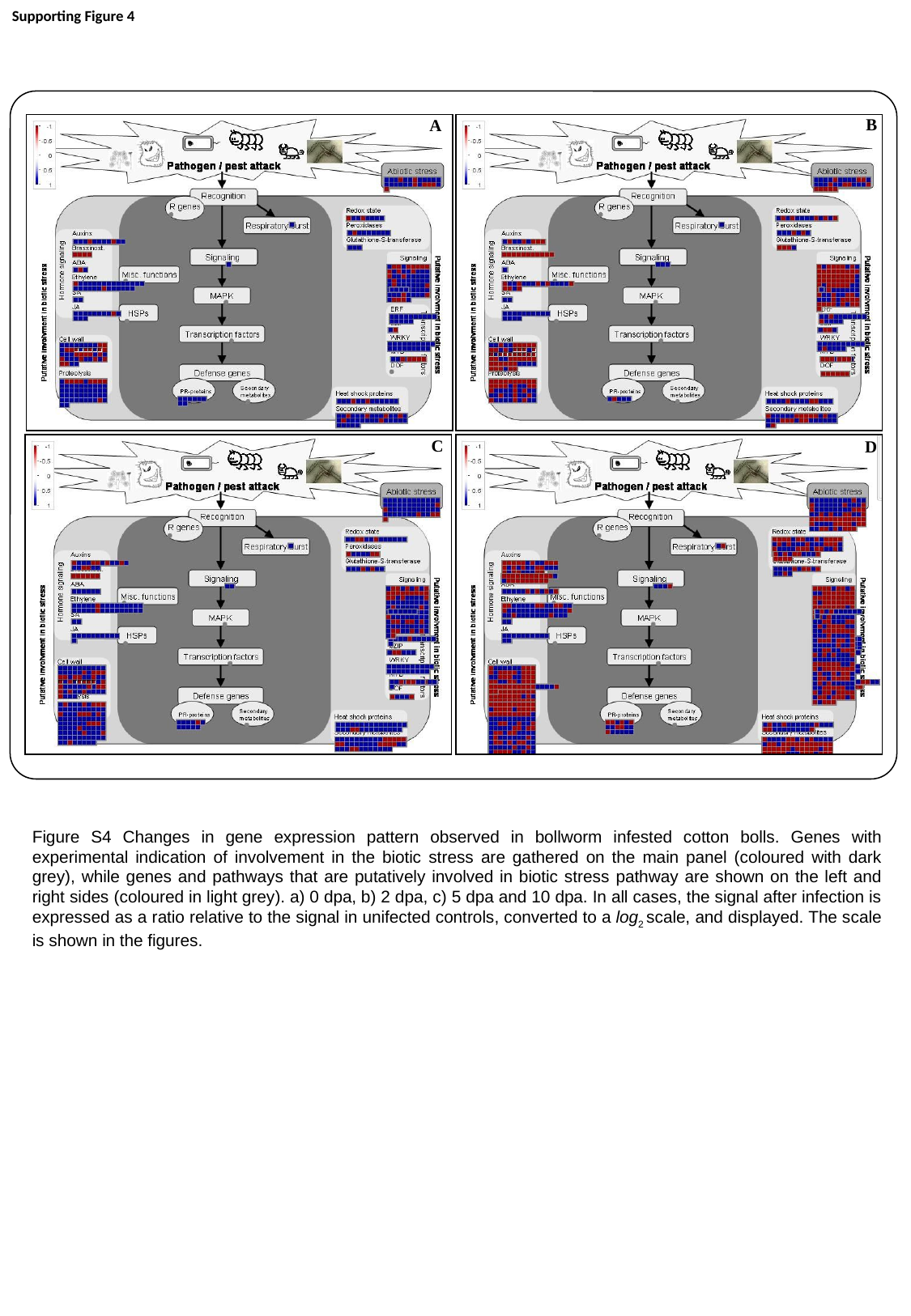

Supporting Figure 4
B
A
C
D
Figure S4 Changes in gene expression pattern observed in bollworm infested cotton bolls. Genes with experimental indication of involvement in the biotic stress are gathered on the main panel (coloured with dark grey), while genes and pathways that are putatively involved in biotic stress pathway are shown on the left and right sides (coloured in light grey). a) 0 dpa, b) 2 dpa, c) 5 dpa and 10 dpa. In all cases, the signal after infection is expressed as a ratio relative to the signal in unifected controls, converted to a log2 scale, and displayed. The scale is shown in the figures.
