# Supplementary material for: Genome‐wide transcriptomic and proteomic analyses of bollworm‐infested developing cotton bolls revealed the genes and pathways involved in the insect pest defence mechanism
Source: Plant Biotechnol J. 2016 Jan 22;14(6):1438–55. doi: 10.1111/pbi.12508 (PMC5066800; doi:10.1111/pbi.12508)
Supplement: Supplementary file 5 — Table S4 Consistently up‐regulated genes in different developmental stages under biotic stress. [file PBI-14-1438-s018.doc]

| **Supporting Table S4:** Consistently up-regulated genes in different developmental stages under biotic stress. | | | | | | | |
| --- | --- | --- | --- | --- | --- | --- | --- |
| **S.No** | **Probeset ID** | **Accession No.** | **Developmental stages**  **(Log_2_ Transformed fold change values)** | | | | **Function** |
|  |  |  | 0 dpa | 2 dpa | 5 dpa | 10 dpa |  |
| 1 | GbaAffx.196.1.A1_at | AY572462.1 | 6.11 | 8.38 | 3.74 | 5.80 | Unknown |
| 2 | GhiAffx.25545.1.A1_at | DW516886.1 | 9.46 | 14.10 | 9.97 | 10.59 | Unknown |
| 3 | Ghi.3251.1.S1_at | DT464395 | 8.22 | 10.05 | 4.96 | 3.15 | basic helix-loop-helix (bHLH) family protein |
| 4 | Ghi.9253.2.A1_a_at | Ghi.9253 | 15.16 | 18.26 | 14.06 | 5.95 | SKS5 (SKU5 Similar 5); copper ion binding / oxidoreductase |
| 5 | GraAffx.28388.1.S1_s_at | CO086694 | 5.69 | 6.35 | 4.82 | 3.31 | Unknown |
| 6 | GraAffx.11740.1.A1_s_at | CO074425 | 5.40 | 9.69 | 6.70 | 3.53 | Unknown 5 |
| 7 | Ghi.10462.1.S1_at | DN758022 | 20.77 | 3.65 | 7.14 | 3.32 | GASA1 (GAST1 PROTEIN HOMOLOG 1) |
| 8 | Ghi.8424.1.A1_at | CO490790 | 21.43 | 4.66 | 8.11 | 5.69 | Transcribed locus |
| 9 | GhiAffx.36503.1.S1_at | DW238688.1 | 23.33 | 4.21 | 8.37 | 5.78 | ATGOLS1 (ARABIDOPSIS THALIANA GALACTINOL SYNTHASE 1) |
| 10 | Ghi.7954.1.S1_s_at | DT468555 | 20.40 | 6.73 | 7.79 | 6.90 | ATMPK3 (MITOGEN-ACTIVATED PROTEIN KINASE 3) |
| 11 | GhiAffx.24550.1.S1_at | DN818231 | 15.28 | 4.62 | 4.70 | 5.92 | calcium-binding EF hand family protein |
| 12 | Ghi.3743.1.A1_at | DT462103 | 124.03 | 61.51 | 10.29 | 29.29 | Transcribed locus |
| 13 | Ghi.9201.1.A1_at | CA992774 | 27.28 | 10.10 | 4.07 | 8.05 | Transcribed locus |
| 14 | GhiAffx.28787.1.S1_at | DT468526 | 39.04 | 12.49 | 4.66 | 6.12 | Unknown |
| 15 | GhiAffx.44107.1.S1_at | DW498292.1 | 16.86 | 9.67 | 4.89 | 3.32 | basic helix-loop-helix (bHLH) |
| 16 | GhiAffx.43038.1.S1_at | DW497938.1 | 36.36 | 18.85 | 14.13 | 10.54 | JMT (JASMONIC ACID CARBOXYL METHYLTRANSFERASE) |
| 17 | GhiAffx.60504.1.A1_s_at | DW504671.1 | 43.63 | 17.09 | 17.78 | 11.86 | Unknown |
| 18 | Ghi.9268.1.S1_x_at | DT463525 | 25.52 | 7.69 | 10.93 | 3.46 | 2-oxoglutarate-dependent dioxygenase |
| 19 | Ghi.1649.1.S1_at | DN760171 | 6.73 | 6.47 | 27.91 | 12.04 | Transcribed locus |
| 20 | Ghi.1732.2.S1_a_at | DN759635 | 7.11 | 6.37 | 27.00 | 11.71 | Transcribed locus |
| 21 | Ghi.1732.3.S1_x_at | DN760161 | 6.32 | 6.21 | 28.43 | 13.57 | Transcribed locus |
| 22 | Ghi.10577.1.S1_at | DN760777 | 7.51 | 6.46 | 25.71 | 13.40 | Transcribed locus |
| 23 | Ghi.6799.1.S1_s_at | CA993132 | 5.63 | 4.21 | 18.61 | 10.40 | Transcribed locus |
| 24 | Ghi.10750.2.S1_s_at | DT553332 | 7.04 | 5.97 | 21.84 | 10.58 | heavy-metal-associated domain-containing protein |
| 25 | Ghi.1617.1.S1_at | DN759663 | 4.40 | 4.43 | 14.05 | 6.89 | Transcribed locus |
| 26 | GhiAffx.59913.1.A1_s_at | DW501774.1 | 3.93 | 3.41 | 10.81 | 6.05 | GHMP kinase family protein |
| 27 | GhiAffx.29447.1.S1_at | DN758263 | 5.05 | 7.05 | 20.02 | 11.24 | Unknown |
| 28 | Ghi.4550.1.A1_at | DT051102 | 7.93 | 3.08 | 21.12 | 15.82 | zinc finger (C3HC4-type RING finger) family protein |
| 29 | Ghi.5353.1.A1_s_at | DT048102 | 7.13 | 3.66 | 27.90 | 16.69 | Transcribed locus |
| 30 | GhiAffx.23106.1.A1_at | CO493702 | 8.31 | 3.38 | 26.74 | 15.33 | Transcribed locus |
| 31 | Ghi.1732.1.S1_at | DN758631 | 5.39 | 5.55 | 15.73 | 11.29 | Transcribed locus |
| 32 | GhiAffx.47990.1.S1_at | DW236910.1 | 3.18 | 3.14 | 9.42 | 6.05 | Transcribed locus |
| 33 | Ghi.10388.2.S1_at | DT469076 | 6.12 | 3.15 | 10.45 | 6.20 | CIPK6 (CBL-INTERACTING PROTEIN KINASE 6) |
| 34 | Ghi.3278.1.A1_s_at | DT467721 | 6.49 | 3.10 | 9.23 | 6.40 | weakly similar to NP_001064203.1 Os10g0159800 |
| 35 | GhiAffx.34421.1.A1_s_at | DW226485.1 | 6.24 | 3.22 | 9.04 | 5.98 | moderately similar to NP_001043963.1 Os01g0695800 |
| 36 | Ghi.10750.1.S1_at | DT462864 | 5.55 | 3.69 | 8.43 | 5.35 | heavy-metal-associated domain-containing protein |
| 37 | GhiAffx.62078.1.S1_at | DW517172.1 | 4.43 | 3.01 | 7.31 | 4.03 | Glutaredoxin family protein |
| 38 | GhiAffx.16921.1.A1_at | DW229809.1 | 5.18 | 3.22 | 10.57 | 5.24 | unknown protein |
| 39 | Ghi.10640.1.S1_s_at | DN780720 | 3.17 | 6.44 | 14.67 | 5.70 | strongly similar to NP_001055754.1 Os05g0460000 |
| 40 | Ghi.9308.1.S1_at | DT527234 | 5.41 | 7.22 | 15.12 | 4.47 | 17.6 kDa class I small heat shock protein |
| 41 | GhiAffx.11624.1.S1_s_at | DW505128.1 | 3.84 | 3.79 | 10.39 | 3.18 | predicted protein |
| 42 | Ghi.5451.1.S1_at | DQ122174.1 | 29.71 | 5.56 | 44.57 | 17.73 | 1-aminocyclopropane-1-carboxylate synthase (ACCS-1) |
| 43 | Ghi.5515.1.A1_s_at | DT463826 | 21.09 | 3.26 | 33.76 | 8.08 | ATFER4 (FERRITIN 4); ferric iron binding |
| 44 | Ghi.1479.1.S1_at | DN781218 | 12.48 | 3.19 | 48.64 | 14.45 | unknown protein |
| 45 | GhiAffx.61299.1.S1_at | DW508705.1 | 24.03 | 14.33 | 153.28 | 45.70 | trypsin and protease inhibitor family protein / Kunitz family protein |
| 46 | Ghi.6855.1.A1_s_at | CA992864 | 6.72 | 4.24 | 62.69 | 28.93 | ProT2 (PROLINE TRANSPORTER 2) |
| 47 | Ghi.1005.1.A1_at | DR452316 | 25.48 | 4.56 | 102.58 | 13.18 | Transcribed locus |
| 48 | Ghi.7933.1.S1_at | AF410458.1 | 33.95 | 3.78 | 198.20 | 8.16 | Polygalacturonase |
| 49 | Ghi.5561.1.A1_s_at | DT047327 | 5.48 | 5.23 | 52.94 | 6.54 | acid phosphatase |
| 50 | GhiAffx.63762.1.S1_s_at | DW240040.1 | 5.43 | 5.44 | 60.20 | 6.53 | acid phosphatase |
| 51 | Ghi.1681.1.S1_at | DN759998 | 3.89 | 16.24 | 48.89 | 10.73 | Nicotianamine synthase |
| 52 | Ghi.2847.1.S1_s_at | DT465458 | 25.30 | 8.09 | 4.94 | 15.90 | ATEP3 (Arabidopsis thaliana chitinase class IV); chitinase |
| 53 | Ghi.6616.1.A1_at | DR460259 | 13.76 | 3.07 | 3.03 | 8.25 | Transcribed locus |
| 54 | GhiAffx.11150.1.S1_at | DW498534.1 | 16.47 | 3.32 | 3.63 | 8.49 | GATL10 (Galacturonosyl transferase-like 10) |
| 55 | Ghi.9194.1.S1_a_at | DN826143 | 56.10 | 4.77 | 7.07 | 30.75 | unknown protein |
| 56 | Ghi.9194.3.S1_x_at | CD485682 | 55.41 | 5.27 | 6.51 | 24.42 | unknown protein |
| 57 | GbaAffx.196.1.A1_s_at | AY572462.1 | 58.16 | 10.37 | 21.92 | 45.61 | Unknown |
| 58 | Ghi.7874.1.S1_s_at | AY962572.1 | 56.68 | 9.65 | 20.61 | 50.60 | Ethylene responsive element binding protein 3 /// Ethylene-responsive element binding protein ERF6 |
| 59 | Ghi.9194.2.A1_x_at | DT468981 | 11.79 | 3.80 | 3.36 | 11.01 | unknown protein |
| 60 | Ghi.3763.1.A1_s_at | DT461952 | 8.69 | 3.30 | 4.96 | 7.63 | calcium-transporting ATPase, Ca(2+)-ATPase, putative (ACA12) |
| 61 | Ghi.9176.3.A1_at | DT464586 | 10.01 | 4.05 | 5.59 | 9.88 | short-chain dehydrogenase/reductase (SDR) family protein |
| 62 | Ghi.3427.1.A1_s_at | DT465993 | 21.97 | 8.45 | 7.89 | 13.58 | Unknown |
| 63 | Ghi.9192.1.S1_s_at | DT468825 | 11.55 | 4.96 | 4.87 | 8.88 | WRKY transcription factor 2 (WRKY) |
| 64 | GhiAffx.39417.1.A1_at | DW499030.1 | 6.45 | 3.24 | 3.29 | 4.72 | Unknown |
| 65 | Ghi.9178.3.S1_at | DT463164 | 12.67 | 3.30 | 6.59 | 20.58 | GCN5-related N-acetyltransferase (GNAT) family protein |
| 66 | GhiAffx.7596.1.S1_at | DW513449.1 | 14.97 | 3.69 | 4.88 | 20.49 | unknown protein |
| 67 | Ghi.10639.1.S1_s_at | DT051576 | 4.96 | 3.07 | 6.01 | 3.43 | COR47 (cold regulated 47) |
| 68 | GhiAffx.44610.1.S1_s_at | DW519438.1 | 10.15 | 5.13 | 11.35 | 6.19 | protein kinase family protein |
| 69 | GhiAffx.3083.4.S1_s_at | DT456106 | 8.10 | 3.06 | 7.29 | 4.01 | Unknown |
| 70 | Ghi.3286.1.S1_at | DT467648 | 12.89 | 9.46 | 11.44 | 7.37 | STZ (SALT TOLERANCE ZINC FINGER) |
| 71 | Ghi.6171.1.A1_at | DT048318 | 9.47 | 6.42 | 7.79 | 4.04 | Transcribed locus |
| 72 | Ghi.374.1.A1_at | DR460282 | 4.94 | 3.21 | 3.97 | 3.25 | MAP3KA (Mitogen-activated protein kinase kinase kinase 3) |
| 73 | GhiAffx.52180.1.S1_s_at | DW237149.1 | 21.90 | 13.73 | 16.97 | 16.72 | phosphoric monoester hydrolase |
| 74 | Ghi.8610.1.S1_s_at | CA992956 | 9.59 | 4.69 | 5.28 | 4.56 | CCR4-NOT transcription complex protein |
| 75 | GhiAffx.4691.1.A1_at | DW517948.1 | 26.99 | 14.10 | 18.17 | 14.10 | ATGA2OX1 (GIBBERELLIN 2-OXIDASE 1); gibberellin 2-beta-dioxygenase |
| 76 | GhiAffx.58305.1.S1_s_at | DW243603.1 | 11.50 | 6.23 | 7.15 | 5.27 | ATAF1 (Arabidopsis NAC domain containing protein 2) |
| 77 | Ghi.5128.1.A1_at | DT048961 | 23.53 | 3.39 | 23.45 | 12.13 | Transcribed locus |
| 78 | GraAffx.28264.1.A1_s_at | CO091105 | 34.86 | 4.09 | 18.68 | 7.57 | Unknown |
| 79 | GhiAffx.30199.1.S1_at | DW506814.1 | 30.64 | 3.93 | 22.82 | 43.52 | transcription factor WRKY22 |
| 80 | Ghi.7950.1.S1_at | AY366083.1 | 400.13 | 3.34 | 16.95 | 61.08 | POD9 precursor (pod9) |
| 81 | GhiAffx.8053.1.A1_at | DW518810.1 | 77.58 | 5.85 | 7.53 | 10.77 | Unknown |
| 82 | Ghi.4920.1.A1_at | DN758194 | 17.44 | 47.99 | 9.66 | 26.13 | Transcribed locus |
| 83 | Ghi.1865.3.A1_x_at | DV849838 | 5.33 | 10.92 | 3.16 | 5.05 | moderately similar to NP_001053524.1 Os04g0556300 |
| 84 | GhiAffx.12687.1.A1_at | DW489633.1 | 4.07 | 7.55 | 3.22 | 4.24 | Unknown |
| 85 | Ghi.10443.1.S1_at | DT049130 | 14.35 | 19.22 | 3.42 | 8.38 | AP2 domain-containing transcription factor |
| 86 | Ghi.6739.1.A1_at | CA993410 | 21.23 | 16.77 | 5.36 | 8.97 | Transcribed locus |
| 87 | GhiAffx.15325.1.S1_at | DT466359 | 12.70 | 11.78 | 4.74 | 4.07 | O-methyltransferase family 2 protein |
| 88 | Ghi.7921.1.S1_x_at | DQ303122.1 | 26.66 | 15.21 | 10.82 | 16.52 | 1-aminocyclopropane-1-carboxylate oxidase (ACO1) |
| 89 | Ghi.7935.2.A1_s_at | DT466133 | 10.14 | 5.58 | 3.51 | 5.68 | L-ascorbate oxidase |
| 90 | Ghi.4983.1.A1_at | DV849718 | 9.95 | 6.20 | 3.09 | 4.70 | STZ (SALT TOLERANCE ZINC FINGER) |
| 91 | Ghi.9169.1.S1_s_at | DT463346 | 12.96 | 7.94 | 3.50 | 6.30 | Transcribed locus |
| 92 | GhiAffx.6766.1.S1_at | DW504268.1 | 34.38 | 19.25 | 11.08 | 13.38 | weakly similar to NP_001044456.1 Os01g0783700 |
| 93 | Ghi.3801.1.A1_at | DT461612 | 49.54 | 44.23 | 10.55 | 30.56 | ANAC090 (Arabidopsis NAC domain containing protein 90) |
| 94 | GhiAffx.25253.1.S1_at | DW519559.1 | 24.48 | 16.84 | 4.78 | 14.35 | unknown protein |
| 95 | Ghi.6732.1.A1_at | CA993437 | 7.41 | 13.10 | 4.46 | 11.36 | unknown protein |
| 96 | Ghi.6873.1.A1_at | CA992796 | 5.03 | 11.70 | 3.75 | 11.21 | Transcribed locus |
| 97 | GhiAffx.21532.1.S1_at | CA993875 | 14.24 | 63.24 | 21.78 | 59.99 | pentatricopeptide (PPR) repeat-containing protein |
| 98 | Ghi.9584.4.S1_at | DT049252 | 7.77 | 27.64 | 12.62 | 21.21 | oxidoreductase, zinc-binding dehydrogenase family protein |
| 99 | Gra.38.1.S1_s_at | CO123081 | 5.32 | 15.23 | 5.91 | 11.39 | Transcribed locus |
| 100 | Ghi.10613.1.S1_at | DT466039 | 9.37 | 19.51 | 3.29 | 4.76 | FAD8 (FATTY ACID DESATURASE 8); omega-3 fatty acid desaturase |
| 101 | Ghi.6765.1.A1_at | CA993268 | 7.28 | 22.92 | 3.22 | 5.11 | BAP2 (BON ASSOCIATION PROTEIN 2) |
| 102 | GhiAffx.5925.1.S1_at | DW502097.1 | 7.21 | 22.62 | 4.89 | 6.98 | BAP2 (BON ASSOCIATION PROTEIN 2) |
| 103 | Ghi.9122.1.A1_at | DT463587 | 4.75 | 13.44 | 10.01 | 13.59 | Transcribed locus |
| 104 | GhiAffx.2041.1.A1_s_at | DT462875 | 3.19 | 10.63 | 14.63 | 10.03 | Unknown |
| 105 | GhiAffx.4536.2.S1_at | DW499238.1 | 3.35 | 11.30 | 11.59 | 10.13 | trypsin and protease inhibitor family protein / Kunitz family protein |
| 106 | Ghi.10388.3.S1_at | CA993635 | 4.69 | 3.67 | 7.72 | 5.72 | CIPK6 (CBL-INTERACTING PROTEIN KINASE 6) |
| 107 | Ghi.3389.1.A1_s_at | DT466538 | 7.57 | 6.32 | 11.25 | 9.52 | Transcribed locus |
| 108 | Ghi.965.2.S1_at | DT467116 | 3.46 | 3.25 | 5.48 | 4.48 | weakly similar to NP_001042083.1 Os01g0159300 |
| 109 | Ghi.906.1.S1_at | DR453362 | 3.12 | 4.47 | 6.69 | 6.11 | kinase interacting family protein |
| 110 | GhiAffx.24039.1.S1_at | DW509198.1 | 3.20 | 3.58 | 6.37 | 4.82 | Unknown |
| 111 | Ghi.6855.1.S1_s_at | CA992864 | 6.35 | 9.84 | 18.11 | 20.58 | ProT2 (PROLINE TRANSPORTER 2) |
| 112 | Ghi.8208.2.A1_s_at | DT462524 | 3.72 | 3.22 | 9.31 | 8.51 | GDH2 (GLUTAMATE DEHYDROGENASE 2); oxidoreductase |
| 113 | Ghi.5565.5.S1_s_at | DT465300 | 4.67 | 4.15 | 5.61 | 8.23 | ATPDR12/PDR12 (PLEIOTROPIC DRUG RESISTANCE 12) |
| 114 | Ghi.3084.1.S1_s_at | DT527319 | 3.45 | 3.12 | 5.56 | 7.98 | hypothetical protein GSPATT00026983001 [Paramecium tetraurelia strain d4-2] |
| 115 | GhiAffx.40480.2.S1_at | DW497946.1 | 4.03 | 4.02 | 6.62 | 8.35 | SOS3 (SALT OVERLY SENSITIVE 3) |
| 116 | Ghi.3337.1.A1_at | DT467161 | 12.27 | 10.09 | 20.18 | 20.89 | Terpene synthase 8 |
| 117 | GhiAffx.4349.1.A1_s_at | DW509077.1 | 4.37 | 3.72 | 6.81 | 6.93 | ATBZIP53 (BASIC REGION/LEUCINE ZIPPER MOTIF 53) |
| 118 | Ghi.7909.1.A1_at | DT463373 | 5.38 | 4.83 | 7.29 | 8.53 | weakly similar to NP_001046956.1 Os02g0515600 |
| 119 | Ghi.9389.2.A1_s_at | DT467229 | 5.89 | 4.59 | 8.01 | 8.79 | lipase class 3 family protein |
| 120 | GhiAffx.10976.1.S1_at | DT048640 | 6.13 | 4.09 | 6.69 | 8.59 | Unknown |
| 121 | Ghi.3342.1.S1_at | DT467115 | 4.87 | 6.42 | 6.46 | 5.57 | weakly similar to NP_001042083.1 Os01g0159300 |
| 122 | Ghi.5307.1.A1_at | DT048232 | 5.27 | 8.12 | 9.62 | 6.99 | senescence-associated protein |
| 123 | Ghi.1037.4.S1_s_at | CA993008 | 3.72 | 4.25 | 4.89 | 4.69 | unknown protein |
| 124 | Ghi.9405.1.A1_at | DT463935 | 3.01 | 3.23 | 3.44 | 4.02 | hydroxyproline-rich glycoprotein family protein |
| 125 | Ghi.3458.1.A1_at | DT465599 | 12.32 | 14.09 | 11.89 | 15.41 | pectinesterase family protein |
| 126 | Ghi.8336.1.S1_at | DT554678 | 3.64 | 5.10 | 4.37 | 4.81 | kinase-related |
| 127 | Ghi.8200.1.S1_at | DT467481 | 7.33 | 5.52 | 7.12 | 6.62 | calmodulin-binding protein |
| 128 | GhiAffx.6177.1.S1_at | DW505740.1 | 4.33 | 3.28 | 4.62 | 3.91 | Unknown |
| 129 | GraAffx.28447.1.A1_s_at | CO086505 | 4.37 | 3.64 | 5.50 | 5.12 | Unknown |
| 130 | Ghi.10620.1.S1_at | DT465545 | 10.67 | 3.75 | 8.20 | 12.84 | moderately similar to XP_001753678.1 predicted protein |
| 131 | Ghi.10493.1.S1_s_at | DT466412 | 7.98 | 4.52 | 7.42 | 12.36 | TCH4 (TOUCH 4) |
| 132 | Ghi.9198.2.S1_at | DT466143 | 7.72 | 4.98 | 6.60 | 10.40 | weakly similar to NP_174140.1 unknown protein |
| 133 | GhiAffx.6483.1.S1_at | DW503136.1 | 9.70 | 4.99 | 7.70 | 12.78 | Transcribed locus |
| 134 | Ghi.4796.1.S1_at | CD809347 | 7.07 | 3.32 | 6.77 | 12.68 | cytochrome P450 |
| 135 | Ghi.7988.1.A1_at | DT468627 | 7.01 | 4.12 | 8.35 | 13.72 | Cytidine/deoxycytidylate deaminase family protein |
| 136 | GhiAffx.39399.1.S1_at | DW499761.1 | 9.83 | 6.81 | 12.43 | 18.53 | moderately similar to XP_001756879.1 predicted protein |
| 137 | Ghi.2692.1.S1_at | DT465311 | 6.84 | 3.88 | 4.16 | 13.32 | calmodulin-binding protein |
| 138 | Ghi.7942.3.A1_a_at | DT461486 | 9.53 | 5.43 | 7.01 | 18.94 | Transcribed locus |
| 139 | GhiAffx.16447.1.A1_at | DW232295.1 | 10.84 | 7.60 | 6.99 | 21.76 | Unknown |
| 140 | Ghi.5565.2.S1_a_at | DT468184 | 9.04 | 3.46 | 6.17 | 14.40 | ATPDR12/PDR12 (PLEIOTROPIC DRUG RESISTANCE 12) |
| 141 | Ghi.5565.7.A1_x_at | CD486452 | 8.09 | 3.57 | 5.61 | 12.43 | ATPDR12/PDR12 (PLEIOTROPIC DRUG RESISTANCE 12) |
| 142 | GraAffx.26883.1.A1_s_at | CO090919 | 9.38 | 4.26 | 4.78 | 14.95 | Unknown |
| 143 | Ghi.9198.2.A1_s_at | DT463956 | 11.04 | 4.79 | 8.74 | 21.21 | unknown protein |
| 144 | Ghi.9198.3.S1_s_at | DT464707 | 8.10 | 3.63 | 5.99 | 16.65 | unknown protein |
| 145 | GhiAffx.48251.1.S1_at | DW226755.1 | 7.79 | 3.15 | 7.53 | 16.45 | Unknown |
| 146 | Ghi.10117.2.A1_s_at | DT462947 | 7.07 | 4.60 | 3.93 | 7.56 | nucellin protein |
| 147 | GhiAffx.24594.1.S1_at | DT466937 | 7.50 | 4.90 | 4.09 | 7.52 | protein kinase family protein |
| 148 | Ghi.9151.2.S1_at | DT049218 | 10.04 | 8.79 | 4.52 | 11.01 | RHD2 (ROOT HAIR DEFECTIVE 2) |
| 149 | Ghi.2766.2.S1_s_at | DT550860 | 5.23 | 3.46 | 4.27 | 6.01 | ATL6 (Arabidopsis Tóxicos en Levadura 6) Ubiquitin ligase |
| 150 | Ghi.8128.1.S1_x_at | AY827548.1 | 8.38 | 6.32 | 5.99 | 9.89 | EREB1 transcription factor |
| 151 | GhiAffx.8242.1.S1_s_at | DW234222.1 | 8.80 | 6.21 | 6.47 | 9.91 | unknown protein |
| 152 | Ghi.9232.1.S1_at | CA992854 | 5.03 | 3.44 | 3.61 | 6.76 | Hairpin-induced protein-related / HIN1-related / hairpin-responsive protein-related |
| 153 | Ghi.6693.1.S1_at | CA993655 | 4.12 | 3.72 | 4.10 | 5.81 | Transcribed locus |
| 154 | GhiAffx.5732.1.S1_at | DW235624.1 | 3.60 | 3.39 | 3.62 | 4.80 | Octicosapeptide/Phox/Bem1p (PB1) domain-containing protein |
| 155 | GhiAffx.31742.1.S1_at | DT457950 | 3.90 | 3.77 | 3.42 | 5.57 | Unknown |
| 156 | Ghi.10332.1.A1_x_at | DT465694 | 5.76 | 7.43 | 3.63 | 8.05 | WRKY22 (WRKY DNA-binding protein 22) |
| 157 | Ghi.1092.3.S1_s_at | DT467839 | 8.37 | 12.15 | 4.35 | 15.18 | unknown protein |
| 158 | Ghi.3229.1.A1_at | DT466114 | 9.80 | 13.50 | 5.26 | 17.88 | phosphoric monoester hydrolase |
| 159 | GhiAffx.25338.1.A1_at | DW515811.1 | 8.21 | 10.95 | 4.95 | 15.32 | Unknown |
| 160 | Ghi.10117.1.S1_at | CO492036 | 3.68 | 4.69 | 3.05 | 5.84 | Nucellin protein |
| 161 | GhiAffx.64116.1.S1_at | DW234100.1 | 3.95 | 4.25 | 3.20 | 6.64 | SRO5 (SIMILAR TO RCD ONE 5); NAD+ ADP-ribosyltransferase |
| 162 | Ghi.1074.1.S1_s_at | DN758679 | 5.57 | 6.39 | 3.88 | 14.51 | STZ (SALT TOLERANCE ZINC FINGER) |
| 163 | Ghi.1092.1.A1_x_at | DT462950 | 19.47 | 21.99 | 13.31 | 45.92 | unknown protein |
| 164 | Ghi.3273.1.A1_at | DT466050 | 6.81 | 12.28 | 3.84 | 21.20 | transcription factor WRKY19 |
| 165 | Ghi.9939.2.S1_at | DV848988 | 6.61 | 12.01 | 4.98 | 19.83 | unknown protein |
| 166 | GhiAffx.7649.1.S1_s_at | DW478800.1 | 7.57 | 15.70 | 6.72 | 29.26 | unknown protein |
| 167 | Ghi.1739.4.S1_x_at | DT464580 | 7.96 | 14.44 | 7.55 | 22.56 | LOX3 (Lipoxygenase 3) |
| 168 | Ghi.8752.1.A1_at | DT052934 | 4.08 | 7.75 | 4.45 | 11.49 | Transcribed locus |
| 169 | Ghi.1739.2.S1_s_at | DN759923 | 7.76 | 12.01 | 7.50 | 18.12 | LOX3 (Lipoxygenase 3) |
| 170 | GhiAffx.31049.1.S1_at | DW233289.1 | 3.08 | 4.69 | 3.03 | 7.12 | zinc finger (C3HC4-type RING finger) family protein |
| 171 | GhiAffx.32010.1.A1_at | DT462319 | 3.13 | 4.52 | 3.06 | 7.28 | Transcribed locus |
| 172 | GhiAffx.1589.46.S1_s_at | DW235449.1 | 8.44 | 12.49 | 7.56 | 20.94 | LOX3 (Lipoxygenase 3) |
| 173 | GhiAffx.3951.1.A1_at | DW477290.1 | 3.61 | 5.14 | 3.83 | 8.98 | Unknown |
| 174 | Ghi.8085.1.S1_at | AF329368.1 | 12.11 | 20.59 | 4.35 | 26.19 | Non-symbiotic hemoglobin class 1 (GLB1) |
| 175 | Ghi.9408.1.S1_at | DT463176 | 8.79 | 19.91 | 4.28 | 26.70 | CYP94C1 (cytochrome P450, family 94, subfamily C, polypeptide 1) |
| 176 | Ghi.2559.1.S1_s_at | DT554033 | 40.75 | 21.29 | 8.05 | 49.51 | ATEP3 (Arabidopsis thaliana chitinase class IV); chitinase |
| 177 | GhiAffx.4043.10.S1_at | DN827319 | 13.03 | 8.68 | 3.59 | 23.33 | Unknown |
| 178 | Ghi.5776.1.S1_at | DN760236 | 15.00 | 29.37 | 41.20 | 72.21 | EDA38 (embryo sac development arrest 38) |
| 179 | Ghi.23.3.S1_x_at | DT468433 | 8.65 | 14.89 | 17.79 | 27.02 | ACS1 (ACC SYNTHASE 1); 1-aminocyclopropane-1-carboxylate synthase |
| 180 | GhiAffx.15780.1.A1_s_at | DW224570.1 | 3.93 | 6.46 | 9.12 | 13.96 | RMA1 (Ring finger protein with Membrane Anchor 1) |
| 181 | GhiAffx.19500.1.S1_at | DW243523.1 | 9.18 | 33.73 | 35.67 | 54.63 | ATGOLS1 (ARABIDOPSIS THALIANA GALACTINOL SYNTHASE 1) |
| 182 | Ghi.3192.1.A1_at | DT467396 | 7.01 | 7.24 | 7.93 | 16.06 | Unknown |
| 183 | Ghi.8366.1.S1_s_at | DT462696 | 15.69 | 15.90 | 16.16 | 37.35 | F-box family protein |
| 184 | Ghi.3354.1.S1_at | DT461765 | 4.49 | 7.41 | 5.58 | 13.80 | NADK1 (NAD kinase 1) |
| 185 | Ghi.7940.1.S1_at | AI055179 | 3.61 | 5.93 | 5.67 | 13.19 | SKS5 (SKU5 Similar 5) |
| 186 | GhiAffx.10384.1.S1_at | DN816829 | 3.85 | 5.47 | 5.32 | 11.70 | Transcribed locus |
| 187 | Ghi.23.4.S1_x_at | DT468283 | 12.80 | 15.49 | 15.11 | 28.96 | ACS1 (ACC SYNTHASE 1); 1-aminocyclopropane-1-carboxylate synthase |
| 188 | GhiAffx.7633.1.S1_s_at | DW499904.1 | 4.66 | 6.13 | 5.14 | 9.95 | FATB (FATTY ACYL-ACP THIOESTERASES B); acyl carrier/ acyl-ACP thioesterase |
| 189 | Ghi.6676.1.S1_s_at | CA993443 | 6.62 | 9.19 | 8.83 | 15.14 | weakly similar to NP_189461.1 PMZ |
| 190 | Gra.2632.1.S1_at | CO083160 | 5.06 | 8.60 | 7.47 | 13.16 | Unknown |
| 191 | Ghi.6875.2.A1_at | DT466966 | 7.19 | 21.82 | 14.16 | 43.34 | weakly similar to XP_001769087.1 predicted protein |
| 192 | GraAffx.20159.1.A1_s_at | CO110779 | 4.69 | 18.42 | 13.57 | 29.60 | Unknown |
| 193 | Ghi.3270.2.S1_at | DT461945 | 8.01 | 9.16 | 3.74 | 25.20 | Transcribed locus |
| 194 | GhiAffx.12869.1.S1_at | DT464191 | 8.88 | 15.33 | 4.66 | 37.51 | Unknown |
| 195 | Ghi.3729.1.A1_at | DT462226 | 3.31 | 5.97 | 7.02 | 26.30 | Transcribed locus |
| 196 | Ghi.1987.1.S1_a_at | DR461896 | 5.67 | 12.30 | 9.65 | 37.97 | unknown protein |
| 197 | GhiAffx.41420.1.S1_at | DW502846.1 | 10.26 | 14.92 | 14.39 | 53.22 | unknown protein |
| 198 | Ghi.10366.2.S1_at | AW561926 | 9.78 | 17.12 | 9.51 | 72.65 | unknown protein |
| 199 | GhiAffx.41710.1.S1_at | DW504385.1 | 4.47 | 7.28 | 4.98 | 30.21 | protease inhibitor/seed storage/lipid transfer protein (LTP) family protein |
| 200 | GhiAffx.3411.1.A1_at | DW497356.1 | 5.07 | 12.55 | 6.23 | 44.29 | zinc finger (C2H2 type) family protein |
| 201 | GhiAffx.25508.1.S1_at | DW496030.1 | 28.72 | 49.80 | 15.07 | 183.63 | moderately similar to XP_001779530.1 predicted protein |
| 202 | GhiAffx.34543.1.A1_at | DW227458.1 | 3.98 | 29.09 | 7.78 | 84.01 | ATGOLS2 (ARABIDOPSIS THALIANA GALACTINOL SYNTHASE 2) |
| 203 | Ghi.10830.1.A1_at | CF932118 | 8.01 | 14.15 | 13.51 | 87.95 | protease inhibitor/seed storage/lipid transfer protein (LTP) family protein |
| 204 | GhiAffx.48901.1.S1_at | DW518199.1 | 3.52 | 4.25 | 4.58 | 29.69 | Unknown |
| 205 | GhiAffx.19697.1.A1_s_at | DW227913.1 | 19.33 | 25.30 | 29.71 | 216.74 | unknown protein |
| 206 | Ghi.9150.1.S1_at | DT463688 | 15.95 | 26.16 | 13.87 | 156.02 | Transcribed locus |
| 207 | GhiAffx.2752.1.S1_at | CA993163 | 8.58 | 12.83 | 9.66 | 91.46 | ATGSTU23 (Arabidopsis thaliana Glutathione S-transferase (class tau) 23) |
| 208 | Gra.47.3.S1_x_at | CO098885 | 3.81 | 10.29 | 8.42 | 67.51 | MSS1; carbohydrate transporter/ sugar porter |
| 209 | Ghi.23.4.A1_at | DT466530 | 262.57 | 12.66 | 341.21 | 486.53 | ACS1 (ACC SYNTHASE 1); 1-aminocyclopropane-1-carboxylate synthase |
| 210 | Ghi.563.2.A1_at | DT463965 | 31.98 | 3.22 | 49.39 | 68.40 | weakly similar to XP_001755863.1 predicted protein |
| 211 | GhiAffx.18577.1.A1_at | DT048511 | 27.06 | 6.22 | 41.45 | 62.24 | weakly similar to NP_001052173.1 Os04g0179100 [Oryza sativa |
| 212 | Ghi.10155.1.S1_at | CD486357 | 6.44 | 3.22 | 8.87 | 18.29 | UDP-glucoronosyl/UDP-glucosyl transferase family protein |
| 213 | Ghi.6722.1.S1_s_at | M88322.1 | 6.95 | 5.35 | 12.32 | 24.82 | Group 4 late embryogenesis-abundant protein (Lea14-A) |
| 214 | GhiAffx.33925.1.S1_at | CA993486 | 6.48 | 4.19 | 11.55 | 23.89 | moderately similar to XP_001779530.1 predicted protein |
| 215 | Ghi.4855.1.A1_at | CA993640 | 3.85 | 3.21 | 7.31 | 13.48 | ATCP1 (CA2+-BINDING PROTEIN 1) |
| 216 | Ghi.6822.1.A1_s_at | CA993006 | 13.41 | 12.92 | 24.69 | 45.85 | UGT72C1 (UDP-glucosyl transferase 72C1); UDP-glycosyl transferase |
| 217 | GhiAffx.16184.1.S1_at | DW506256.1 | 3.15 | 4.23 | 7.71 | 13.69 | EIN3a transcription factor |
| 218 | Ghi.10251.4.S1_s_at | DT543102 | 3.20 | 3.13 | 4.89 | 9.26 | aminotransferase class I and II family protein |
| 219 | Ghi.7941.1.S1_at | AI055173 | 5.13 | 4.79 | 7.49 | 13.61 | Transcribed locus |
| 220 | Ghi.738.1.A1_at | DT467171 | 4.22 | 3.01 | 5.92 | 10.23 | Transcribed locus |
| 221 | GraAffx.34007.1.A1_s_at | CO070199 | 9.02 | 6.36 | 12.37 | 21.79 | Unknown |
| 222 | Ghi.3632.1.A1_at | DT463493 | 4.18 | 3.30 | 9.48 | 13.80 | unknown protein |
| 223 | GhiAffx.19570.1.S1_at | DW489734.1 | 11.13 | 7.36 | 28.58 | 41.87 | PLP2 (PHOSPHOLIPASE A 2A) |
| 224 | GhiAffx.12228.1.S1_at | DW512183.1 | 3.48 | 3.78 | 12.66 | 18.34 | AAA-type ATPase family protein |
| 225 | Ghi.6013.1.S1_s_at | DR453148 | 3.43 | 3.19 | 10.42 | 13.08 | unknown protein |
| 226 | GhiAffx.28716.1.S1_at | DW512426.1 | 4.99 | 3.31 | 14.97 | 18.57 | auxin-responsive family protein |
| 227 | Ghi.6320.2.S1_at | DT462221 | 3.57 | 4.31 | 10.50 | 13.89 | ATTPS7 (Arabidopsis thaliana trehalose-phosphatase/synthase 7) |
| 228 | Ghi.1621.1.S1_x_at | DN760810 | 4.84 | 6.26 | 13.27 | 19.02 | unknown protein |
| 229 | Gra.1530.2.S1_s_at | CO125258 | 3.59 | 3.80 | 9.18 | 12.89 | WRKY3; transcription factor |
| 230 | GhiAffx.11775.1.S1_at | DW224036.1 | 4.49 | 6.10 | 14.03 | 20.91 | unknown protein |
| 231 | Ghi.7171.1.S1_at | AW187461 | 5.60 | 4.54 | 12.63 | 16.29 | Transcribed locus |
| 232 | Gra.2105.1.A1_s_at | CO123877 | 3.76 | 4.07 | 7.89 | 11.52 | ACBP1 (ACYL-COA BINDING PROTEIN) |
| 233 | GarAffx.5818.1.S1_s_at | BF278946 | 15.21 | 4.82 | 23.24 | 62.83 | Unknown |
| 234 | Ghi.3288.2.S1_at | DT467632 | 8.45 | 5.02 | 13.59 | 37.93 | CYP76C5 (cytochrome P450, family 76, subfamily C, polypeptide 5) |
| 235 | Ghi.3263.1.A1_at | DT467895 | 14.35 | 3.28 | 23.75 | 67.36 | ATCSLG2 (Cellulose synthase-like G2) |
| 236 | GhiAffx.24975.1.A1_at | DW513882.1 | 35.16 | 4.67 | 49.73 | 151.21 | Unknown |
| 237 | Ghi.6088.2.S1_at | DT467539 | 9.53 | 3.27 | 21.63 | 54.54 | ATMPK3 (MITOGEN-ACTIVATED PROTEIN KINASE 3) |
| 238 | Ghi.1889.1.S1_at | DV849586 | 3.45 | 4.21 | 11.92 | 22.96 | lipase class 3 family protein |
| 239 | Ghi.1202.1.S1_at | DT468707 | 4.19 | 3.99 | 12.40 | 26.42 | AAE7/ACN1 (ACYL-ACTIVATING ENZYME 7); AMP binding / acetate-CoA ligase |
| 240 | Ghi.6141.2.A1_s_at | DR458687 | 3.31 | 3.53 | 11.37 | 23.48 | SDP1 (SUGAR-DEPENDENT1); triacylglycerol lipase |
| 241 | Ghi.3451.2.S1_at | DT464684 | 10.09 | 4.44 | 20.64 | 38.61 | ATTPS11 (Arabidopsis thaliana trehalose phosphatase/synthase 11) |
| 242 | Ghi.2608.2.S1_at | DT463212 | 4.38 | 3.86 | 10.29 | 19.27 | unknown protein |
| 243 | Gra.275.1.S1_s_at | CO090219 | 5.40 | 4.28 | 13.30 | 25.46 | moderately similar to NP_564761.1 ATB2 |
| 244 | GhiAffx.24157.1.A1_at | DW509771.1 | 4.43 | 3.78 | 11.22 | 19.30 | unknown protein |
| 245 | Ghi.3049.2.S1_at | DT464459 | 5.63 | 3.35 | 28.42 | 47.82 | Haloacid dehalogenase-like hydrolase family protein |
| 246 | Ghi.3184.1.S1_s_at | DT468147 | 4.56 | 3.20 | 22.53 | 38.87 | AAE7/ACN1 (ACYL-ACTIVATING ENZYME 7) |
| 247 | Ghi.6429.1.S1_at | CF932111 | 33.78 | 3.59 | 123.01 | 216.32 | Transcribed locus |
| 248 | Ghi.1553.1.S1_at | DT462666 | 5.02 | 4.27 | 6.23 | 14.08 | LAC14 (laccase 14) |
| 249 | Ghi.576.3.A1_x_at | DT462667 | 4.97 | 3.76 | 5.40 | 12.31 | unknown protein |
| 250 | Ghi.5495.1.A1_s_at | DT047529 | 4.46 | 3.18 | 4.79 | 12.13 | hypothetical protein DDBDRAFT_0184442 |
| 251 | Ghi.3234.2.A1_at | DT468165 | 4.80 | 5.71 | 5.27 | 14.15 | NADK1 (NAD kinase 1) |
| 252 | Ghi.3729.1.A1_s_at | DT462226 | 5.18 | 6.45 | 7.22 | 17.45 | Transcribed locus |
| 253 | GhiAffx.4043.12.A1_s_at | DW233918.1 | 5.52 | 6.44 | 6.65 | 17.99 | moderately similar to NP_567701.1 hydrolase, acting on ester bonds |
| 254 | Ghi.3284.1.S1_s_at | DT466688 | 19.92 | 20.84 | 17.77 | 65.50 | unknown protein |
| 255 | Ghi.9266.1.A1_at | DT468530 | 4.82 | 5.36 | 5.68 | 17.25 | L-ascorbate oxidase |
| 256 | Gra.1358.1.A1_at | CO126276 | 5.36 | 5.01 | 6.21 | 17.54 | weakly similar to NP_001064453.1 Os10g0369600 |
| 257 | GhiAffx.40107.1.S1_s_at | DW496119.1 | 24.04 | 15.96 | 16.71 | 66.08 | U-box domain-containing protein |
| 258 | Ghi.5961.1.A1_at | DT468117 | 3.22 | 7.61 | 9.47 | 23.50 | weakly similar to NP_001047232.1 Os02g0580000 |
| 259 | Ghi.6875.1.S1_at | CA992786 | 3.69 | 9.54 | 9.73 | 26.45 | weakly similar to XP_001769087.1 predicted protein |
| 260 | Ghi.5031.1.A1_at | DT049156 | 8.26 | 16.49 | 22.64 | 64.43 | Transcribed locus |
| 261 | GhiAffx.5701.1.S1_at | DW515784.1 | 3.19 | 4.25 | 7.83 | 18.75 | ADOF2 (Arabidopsis dof zinc finger protein 2) |
| 262 | Ghi.9240.1.S1_s_at | DT468893 | 3.33 | 5.33 | 5.47 | 14.81 | transcription factor WRKY23 |
| 263 | GhiAffx.13045.1.S1_at | DW226240.1 | 3.63 | 5.23 | 7.17 | 16.02 | PUB17 (PLANT U-BOX17); ubiquitin-protein ligase |
| 264 | GraAffx.28942.1.S1_s_at | CO085044 | 3.58 | 4.07 | 5.71 | 13.22 | Unknown |
| 265 | Ghi.1492.1.S1_at | DN781087 | 8.39 | 5.34 | 12.19 | 45.76 | CYP76C5 (cytochrome P450, family 76, subfamily C, polypeptide 5) |
| 266 | Ghi.3732.1.S1_s_at | DT462200 | 6.40 | 4.61 | 9.86 | 35.21 | Unknown |
| 267 | Ghi.6898.1.S1_at | CA992714 | 3.44 | 3.17 | 6.01 | 20.92 | oxidoreductase, 2OG-Fe(II) oxygenase family protein |
| 268 | Ghi.9182.3.S1_at | DT461494 | 8.20 | 5.34 | 14.90 | 59.11 | transcription factor WRKY19 |
| 269 | GraAffx.15033.1.S1_at | CO125965 | 4.06 | 3.26 | 8.88 | 33.39 | Unknown |
| 270 | Ghi.4731.1.A1_at | DT051109 | 3.62 | 7.06 | 13.06 | 39.34 | Transcribed locus |
| 271 | GhiAffx.19354.1.S1_s_at | DW488498.1 | 3.95 | 5.46 | 10.60 | 35.22 | UGE5 (UDP-D-glucose/UDP-D-galactose 4-epimerase 5) |
| 272 | Ghi.9102.1.S1_at | DV849822 | 12.55 | 17.21 | 37.63 | 134.95 | weakly similar to NP_001057827.1 Os06g0548200 |
| 273 | Gra.718.1.S1_s_at | CO085887 | 6.91 | 10.53 | 18.72 | 70.65 | NIA1 (NITRATE REDUCTASE 1) |
| 274 | Ghi.6869.1.A1_at | CA992801 | 3.78 | 3.15 | 13.90 | 37.20 | matrixin family protein |
| 275 | Ghi.9236.1.S1_at | DT463077 | 5.56 | 5.31 | 17.47 | 54.98 | DOGT1 (DON-GLUCOSYLTRANSFERASE); UDP-glycosyl transferase |
| 276 | GhiAffx.8694.1.S1_a_at | DW499451.1 | 28.85 | 14.35 | 77.79 | 233.09 | Unknown |
| 277 | Ghi.1011.1.A1_s_at | DT463974 | 6.35 | 8.86 | 30.96 | 116.31 | YLS9 (YELLOW-LEAF-SPECIFIC GENE 9) |
| 278 | Ghi.10595.1.S1_at | DN761009 | 6.10 | 20.74 | 57.09 | 219.11 | unknown protein |
| 279 | Ghi.6656.1.A1_at | CA993777 | 7.94 | 19.56 | 128.20 | 574.76 | Transcribed locus |
| 280 | GhiAffx.4130.1.S1_at | DW498401.1 | 4.47 | 3.69 | 24.09 | 117.02 | zinc finger (C3HC4-type RING finger) family protein |
| 281 | Ghi.9280.3.A1_at | DT462248 | 19.07 | 4.74 | 42.01 | 176.63 | weakly similar to NP_001053341.1 Os04g0522500 |
| 282 | Ghi.9152.2.A1_at | DT463871 | 22.54 | 9.81 | 71.93 | 254.77 | transducin family protein / WD-40 repeat family protein |
| 283 | Ghi.9823.1.S1_at | DT464807 | 5.76 | 5.41 | 29.77 | 97.21 | unknown protein |
| 284 | Ghi.2608.2.A1_at | DT463212 | 10.68 | 4.22 | 67.62 | 226.33 | unknown protein |
| 285 | Ghi.7907.1.S1_s_at | AI055500 | 9.50 | 3.06 | 50.08 | 189.64 | RD26 (RESPONSIVE TO DESSICATION 26) |
| 286 | GhiAffx.7921.1.S1_at | DW517203.1 | 3.83 | 3.39 | 43.23 | 157.10 | Transcribed locus |
| 287 | Ghi.6632.1.A1_s_at | DT461730 | 4.09 | 4.01 | 28.17 | 71.22 | MAPKKK14 (Mitogen-activated protein kinase kinase kinase 14) |
| 288 | GbaAffx.198.1.S1_at | AY560547.1 | 7.50 | 5.77 | 3.95 | 38.40 | Unknown |
| 289 | GbaAffx.198.1.S1_x_at | AY560547.1 | 6.67 | 5.55 | 3.65 | 35.14 | Unknown |
| 290 | Ghi.1069.1.S1_x_at | DN759630 | 31.54 | 28.89 | 8.82 | 155.51 | ATBCB (ARABIDOPSIS BLUE-COPPER-BINDING PROTEIN) |
| 291 | Ghi.3731.2.A1_at | DT467881 | 14.25 | 4.52 | 5.57 | 54.14 | weakly similar to NP_001056779.1 Os06g0143900 |
| 292 | Ghi.1045.1.S1_at | DN826851 | 9.39 | 7.62 | 8.20 | 36.30 | moderately similar to NP_001060901.1 Os08g0127100 |
| 293 | Ghi.1132.2.A1_a_at | DT052025 | 11.37 | 6.33 | 7.83 | 36.15 | WRKY41 (WRKY DNA-binding protein 41) |
| 294 | Ghi.9233.1.A1_at | DT465825 | 9.59 | 6.17 | 8.26 | 43.61 | weakly similar to NP_172572.1 protein kinase family protein |
| 295 | Ghi.2939.2.A1_s_at | DT466107 | 33.53 | 3.65 | 19.82 | 94.72 | AOX1D (ALTERNATIVE OXIDASE 1D) |
| 296 | Ghi.8110.1.S1_at | AY311597.1 | 17.01 | 3.09 | 8.60 | 43.44 | Class III peroxidase (pod7) |
| 297 | Ghi.6443.2.A1_s_at | CF932178 | 11.18 | 3.86 | 5.12 | 29.86 | PR4 (PATHOGENESIS-RELATED 4) |
| 298 | Ghi.7941.1.A1_at | AI055173 | 18.45 | 5.63 | 18.65 | 72.93 | Transcribed locus |
| 299 | Ghi.8496.1.A1_at | DV849555 | 13.05 | 6.55 | 12.61 | 52.10 | wound-responsive protein-related |
| 300 | Ghi.1114.1.S1_s_at | DN817396 | 14.56 | 4.36 | 9.21 | 49.01 | calcium-binding EF hand family protein |
| 301 | GhiAffx.44360.1.S1_at | DW518204.1 | 14.14 | 3.55 | 11.28 | 46.72 | Transcribed locus |
| 302 | GhiAffx.2668.1.A1_at | DT463855 | 28.42 | 6.33 | 23.95 | 117.15 | Unknown |
| 303 | Ghi.10425.3.S1_s_at | DN827840 | 16.48 | 6.68 | 20.31 | 98.22 | NHL3 (NDR1/HIN1-like 3) |
| 304 | Ghi.9271.5.A1_at | DT463804 | 13.57 | 3.31 | 12.33 | 69.24 | Transcribed locus |
| 305 | Ghi.6664.1.S1_at | CA993761 | 25.28 | 12.07 | 23.93 | 149.61 | Transcribed locus |
| 306 | Ghi.4.1.A1_at | CK987701 | 23.04 | 6.34 | 12.18 | 136.43 | weakly similar to NP_001052173.1 Os04g0179100 |
| 307 | Ghi.1036.1.S1_at | DN827073 | 7.15 | 6.08 | 4.76 | 68.05 | Transcribed locus |
| 308 | Ghi.3652.1.S1_s_at | DT463174 | 7.92 | 4.61 | 8.26 | 81.67 | AAT1 (CATIONIC AMINO ACID TRANSPORTER 1) |
| 309 | Ghi.1008.1.S1_at | CK987666 | 11.71 | 17.07 | 10.36 | 233.03 | ATOSM34 (OSMOTIN 34) |
| 310 | Ghi.2169.1.A1_at | DT468931 | 11.99 | 10.05 | 19.80 | 130.07 | GRAM domain-containing protein / ABA-responsive protein-related |
| 311 | Ghi.3328.1.A1_at | DT463239 | 12.35 | 11.03 | 26.25 | 170.59 | cytochrome P450 |
| 312 | Ghi.3232.1.S1_at | DT462100 | 4.85 | 4.49 | 7.50 | 40.42 | MAPKKK14 (Mitogen-activated protein kinase kinase kinase 14) |
| 313 | Ghi.10327.1.S1_s_at | DT463653 | 4.87 | 5.13 | 14.28 | 65.20 | unknown protein |
| 314 | Ghi.6692.1.S1_s_at | CA993159 | 3.55 | 4.45 | 9.34 | 41.35 | Transcribed locus |
| 315 | Ghi.9721.2.S1_at | DV850243 | 3.92 | 3.09 | 12.59 | 79.85 | unknown protein |
| 316 | Ghi.7891.1.S1_s_at | DT462224 | 34.58 | 9.50 | 54.08 | 322.82 | extracellular dermal glycoprotein / EDGP |
| 317 | Ghi.9152.1.S1_at | DT462541 | 6.37 | 6.75 | 18.27 | 148.17 | transducin family protein / WD-40 repeat family protein |
| 318 | GhiAffx.12729.1.A1_at | DT466204 | 3.10 | 7.09 | 13.97 | 122.06 | protein kinase family protein |
| 319 | Ghi.8476.1.S1_x_at | CO495095 | 13.16 | 6.41 | 197.00 | 1360.12 | Transcribed locus |
| 320 | Gra.1633.1.A1_s_at | CO117653 | 9.61 | 3.39 | 157.90 | 907.39 | unknown protein |
| 321 | Ghi.1586.1.S1_at | DN760004 | 12.79 | 3.71 | 32.82 | 44.18 | heavy-metal-associated domain-containing protein |
| 322 | Ghi.1202.1.A1_s_at | DT046611 | 9.25 | 3.14 | 26.82 | 32.98 | AAE7/ACN1 (ACYL-ACTIVATING ENZYME 7); AMP binding / acetate-CoA ligase |
| 323 | Ghi.8294.2.S1_at | AI731982 | 7.08 | 3.03 | 20.32 | 25.34 | ATCK1 (CHOLINE KINASE) |
| 324 | GhiAffx.15571.1.S1_a_at | DW505599.1 | 23.91 | 3.03 | 75.90 | 103.96 | Unknown |
| 325 | Ghi.6849.1.A1_at | CA992877 | 4.35 | 4.19 | 31.63 | 42.13 | ATFTSH6/FTSH6 (FtsH protease 6); ATP-dependent peptidase |
| 326 | Ghi.7876.1.S1_s_at | DN803199 | 7.61 | 3.02 | 27.96 | 40.70 | sulfotransferase family protein |
| 327 | Ghi.807.1.S1_s_at | DT465871 | 11.66 | 8.01 | 56.04 | 79.29 | STZ (SALT TOLERANCE ZINC FINGER) |
| 328 | Ghi.3235.1.A1_at | DT463078 | 9.50 | 5.68 | 40.11 | 41.43 | UGT72C1 (UDP-glucosyl transferase 72C1) |
| 329 | Gra.2045.1.A1_at | CO124159 | 3.44 | 3.18 | 12.39 | 12.01 | TRFL9 (TRF-LIKE 9) |
