# Supplementary material for: Genome‐wide transcriptomic and proteomic analyses of bollworm‐infested developing cotton bolls revealed the genes and pathways involved in the insect pest defence mechanism
Source: Plant Biotechnol J. 2016 Jan 22;14(6):1438–55. doi: 10.1111/pbi.12508 (PMC5066800; doi:10.1111/pbi.12508)
Supplement: Supplementary file 6 — Table S5 Consistently down‐regulated genes in different developmental stages under biotic stress. [file PBI-14-1438-s002.doc]

|  | **Supporting table S5.** Consistently down-regulated genes in different boll developmental stages under biotic stress | | | | | | |
| --- | --- | --- | --- | --- | --- | --- | --- |
| S.No | Probeset ID | Accession No | Developmental stages  (Log_2_ transformed fold change values) | | | | Function |
|  |  |  | 0 dpa | 2 dpa | 5 dpa | 10 dpa |  |
| 1 | Ghi.10609.1.S1_s_at | DN761916 | -3.37 | -3.66 | -3.03 | -59.16 | Polygalacturonase-inhibiting protein (PGIPL) |
| 2 | Ghi.8520.1.S1_at | DR044160 | -5.54 | -6.16 | -6.42 | -102.24 | delta-8 sphingolipid desaturase |
| 3 | Ghi.6188.1.A1_at | CO493635 | -3.49 | -4.23 | -3.13 | -76.94 | ATEXPA4 (ARABIDOPSIS THALIANA EXPANSIN A4) |
| 4 | Gra.2938.2.A1_at | CO085472 | -10.99 | -6.80 | -5.34 | -114.99 | THI1 (THIAZOLE REQUIRING) |
| 5 | Ghi.4573.1.S1_at | DT052179 | 5.83 | -4.02 | 3.70 | -4.49 | GAMMA-TIP (Tonoplast intrinsic protein (TIP) |
| 6 | GarAffx.24390.1.S1_s_at | BF273948 | -10.69 | -4.49 | -3.05 | -8.71 | Unknown |
| 7 | Ghi.2616.1.S1_s_at | DT545336 | -9.63 | -5.11 | -4.05 | -9.47 | xyloglucan: xyloglucosyl transferase/ xyloglucan endotransglycosylase |
| 8 | Gra.994.2.S1_s_at | CO090035 | -8.07 | -3.69 | -5.30 | -8.93 | RNA binding / nucleic acid binding |
| 9 | Ghi.789.1.S1_at | AF116537.2 | -29.89 | -5.99 | -4.89 | -5.89 | Fiber protein GLP1 (Glp1) |
| 10 | Ghi.7226.1.S1_s_at | AW186996 | -3.99 | -28.10 | -4.01 | -72.24 | Transcribed locus |
| 11 | Ghi.2198.1.S1_s_at | DT550268 | -4.13 | -15.92 | -3.85 | -46.39 | Fasciclin-like arabinogalactan protein 11 (FLA11) |
| 12 | GhiAffx.53444.1.A1_s_at | DW229324.1 | -3.35 | -17.51 | -3.71 | -47.35 | Subtilase family protein |
| 13 | GhiAffx.50889.1.S1_x_at | DT048030 | -3.13 | -13.56 | -4.22 | -34.39 | weakly similar to NP_001067715.1 Os11g0294400 |
| 14 | Ghi.4648.2.A1_s_at | AY464057.1 | -3.17 | -4.78 | -3.57 | -11.98 | BURP domain-containing protein |
| 15 | GraAffx.20352.2.S1_s_at | CO117676 | -3.40 | -5.14 | -3.89 | -11.93 | Unknown |
| 16 | GhiAffx.58869.1.S1_at | DW497572.1 | -5.19 | -7.39 | -4.39 | -18.56 | copper-binding family protein |
| 17 | Gra.2038.1.A1_s_at | CO124831 | -3.18 | -5.12 | -3.59 | -13.63 | weakly similar to NP_001052280.1 Os04g0225700 |
| 18 | GraAffx.27319.1.S1_s_at | CO089724 | -7.63 | -9.77 | -3.69 | -20.46 | Unknown |
| 19 | GhiAffx.11783.1.S1_s_at | DW228202.1 | -3.89 | -7.59 | -3.50 | -17.22 | zinc finger (GATA type) family protein |
| 20 | GhiAffx.45244.1.S1_x_at | DT051989 | -5.74 | -15.90 | -5.59 | -29.46 | Unknown |
| 21 | GhiAffx.63198.1.S1_at | DW519856.1 | -3.62 | -8.57 | -3.37 | -16.93 | DVL13/RTFL2 (ROTUNDIFOLIA LIKE 2) |
| 22 | GraAffx.28945.1.S1_s_at | CO085033 | -3.01 | -8.30 | -3.52 | -16.16 | Unknown |
| 23 | GraAffx.23337.2.S1_a_at | CO086497 | -3.24 | -7.46 | -3.51 | -14.57 | Unknown |
| 24 | Ghi.4663.1.A1_x_at | DT051323 | -3.15 | -3.36 | -4.63 | -13.72 | Alpha-tubulin (TUA7) |
| 25 | Ghi.7343.1.S1_at | DR454175 | -3.19 | -3.69 | -4.22 | -13.40 | weakly similar to NP_001049232.1 Os03g0191200 |
| 26 | GhiAffx.23083.1.A1_s_at | DW228819.1 | -3.07 | -5.26 | -5.31 | -16.57 | CYP77A4 (cytochrome P450, family 77, subfamily A, polypeptide 4) |
| 27 | GhiAffx.23436.1.S1_x_at | DR455118 | -3.06 | -6.19 | -7.91 | -18.79 | RALFL34 (RALF-LIKE 34) |
| 28 | GraAffx.15820.1.S1_s_at | CO123702 | -3.68 | -5.99 | -7.07 | -15.72 | Unknown |
| 29 | GhiAffx.30557.1.A1_at | DW485948.1 | -5.04 | -3.43 | -5.10 | -13.68 | unknown protein |
| 30 | Ghi.9734.1.S1_at | AW186914 | -4.10 | -4.88 | -11.25 | -20.50 | long-chain-alcohol O-fatty-acyltransferase family protein / wax synthase family protein |
| 31 | GhiAffx.62337.1.S1_at | DW497105.1 | -3.69 | -3.03 | -7.92 | -15.45 | ATOFP6/OFP6 (Arabidopsis thaliana ovate family protein 6) |
| 32 | Ghi.7283.1.S1_s_at | AI731943 | -4.51 | -3.92 | -6.82 | -9.32 | transferase family protein |
| 33 | Ghi.976.1.S1_s_at | DR452560 | -4.67 | -7.58 | -6.55 | -12.04 | GDSL-motif lipase/hydrolase family protein |
| 34 | GhiAffx.51953.1.S1_s_at | DW507650.1 | -3.22 | -4.56 | -3.55 | -8.92 | Transcribed locus |
| 35 | GraAffx.2019.1.S1_s_at | CO122895 | -3.69 | -5.98 | -6.07 | -12.09 | Unknown |
| 36 | Ghi.7418.1.S1_s_at | DR454725 | -4.34 | -6.46 | -6.21 | -36.24 | unknown protein |
| 37 | Ghi.5009.1.S1_s_at | AI728500 | -3.00 | -5.72 | -3.44 | -17.72 | FAH1 (FATTY ACID HYDROXYLASE 1) |
| 38 | Ghi.5889.2.S1_s_at | AI727736 | -4.95 | -9.90 | -7.21 | -32.25 | transferase family protein |
| 39 | GhiAffx.22625.1.A1_s_at | DW501687.1 | -3.34 | -6.61 | -3.13 | -22.91 | moderately similar to NP_001062915.1 Os09g0338500 |
| 40 | Ghi.5081.1.S1_s_at | DT049160 | -4.42 | -12.51 | -24.31 | -157.08 | glyoxal oxidase-related |
| 41 | Ghi.5267.1.A1_at | DT046626 | -7.718 | -23.01 | -5.66 | -144.03 | GBF6 (G-box binding factor 6) |
| 42 | Ghi.5796.1.S1_s_at | AI730807 | -3.20 | -4.55 | -5.34 | -54.13 | plastocyanin-like domain-containing protein |
| 43 | Ghi.10316.1.S1_s_at | DT051688 | -3.57 | -8.79 | -6.26 | -83.47 | phosphate-responsive 1 family protein |
| 44 | GhiAffx.6042.1.S1_s_at | DW498160.1 | -3.41 | -5.99 | -3.31 | -51.48 | gibberellin-regulated family protein |
| 45 | Ghi.8826.1.S1_s_at | DT566192 | -3.69 | -66.19 | -4.12 | -269.13 | ATLP-1 (Arabidopsis thaumatin-like protein 1) |
| 46 | Gra.1243.1.S1_s_at | CO123292 | -9.46 | -11.26 | -3.47 | -66.14 | unknown protein |
| 47 | Ghi.364.1.S1_s_at | AI731396 | -6.42 | -7.49 | -4.23 | -27.47 | GDSL-motif lipase/hydrolase family protein |
| 48 | Ghi.8492.1.S1_s_at | CD486671 | -6.74 | -4.77 | -3.73 | -20.23 | unknown protein |
| 49 | Ghi.8236.1.S1_s_at | DT567483 | -19.54 | -6.44 | -6.75 | -28.84 | phosphate-responsive 1 family protein |
| 50 | Ghi.10022.2.S1_s_at | DT564348 | -6.83 | -14.19 | -3.02 | -14.51 | 24-sterol C-methyltransferase (SMT2-2) |
| 51 | Ghi.6951.1.S1_x_at | AJ513153 | -5.21 | -12.48 | -3.52 | -13.47 | AN3 (ANGUSITFOLIA3) |
| 52 | Ghi.2725.2.S1_at | DT550027 | -4.59 | -10.87 | -3.11 | -7.76 | weakly similar to NP_001058883.1 Os07g0145400 |
| 53 | GhiAffx.25167.1.A1_at | DW514940.1 | -6.53 | -13.79 | -3.12 | -10.67 | Unknown |
| 54 | GraAffx.34313.1.S1_s_at | CA994247 | -3.86 | -10.17 | -3.96 | -8.89 | Unknown |
| 55 | Ghi.5385.1.S1_s_at | DT047980 | -7.20 | -26.11 | -3.48 | -24.57 | Leucine-rich repeat family protein |
| 56 | GhiAffx.22909.1.S1_at | DW503190.1 | -13.01 | -23.50 | -3.81 | -14.62 | Unknown |
| 57 | Ghi.2137.1.S1_s_at | DT051002 | -3.14 | -4.24 | -3.58 | -4.58 | SCR (SCARECROW) |
| 58 | Ghi.9055.2.A1_at | DT459604 | -3.01 | -4.04 | -3.41 | -3.72 | MEE3 (maternal effect embryo arrest 3) |
| 59 | Ghi.9055.1.A1_at | DT527486 | -3.29 | -5.24 | -3.63 | -4.26 | MEE3 (maternal effect embryo arrest 3) |
| 60 | Ghi.7236.1.S1_s_at | DR459712 | -3.27 | -5.75 | -3.15 | -5.06 | unknown protein |
| 61 | GhiAffx.879.1.S1_at | DN816995 | -3.93 | -5.89 | -3.53 | -5.43 | HDA14 (histone deacetylase 14) |
| 62 | Ghi.9979.1.A1_s_at | DR458170 | -3.33 | -6.05 | -3.95 | -6.01 | weakly similar to NP_001064966.1 Os10g0498200 |
| 63 | Gra.1032.1.S1_s_at | CO123218 | -3.52 | -6.69 | -3.79 | -7.45 | peptidase/ subtilase |
| 64 | Ghi.3912.1.S1_x_at | DT455892 | -3.86 | -6.64 | -3.16 | -11.84 | legume lectin family protein |
| 65 | Ghi.7019.1.A1_s_at | DT048277 | -4.25 | -10.84 | -4.10 | -16.17 | phosphate-responsive 1 family protein |
| 66 | Ghi.8869.1.S1_s_at | DT457129 | -3.85 | -13.9 | -6.78 | -18.69 | leucine-rich repeat transmembrane protein kinase |
| 67 | GhiAffx.42473.1.S1_at | DW508297.1 | -3.82 | -12.73 | -7.35 | -17.58 | IQD11 (IQ-domain 11); calmodulin binding |
| 68 | GraAffx.17633.1.S1_s_at | CO121151 | -3.75 | -8.17 | -3.57 | -9.86 | Unknown |
| 69 | Ghi.1484.1.S1_at | DR460542 | -6.16 | -30.89 | -3.85 | -40.07 | weakly similar to NP_001058178.1 Os06g0643500 |
| 70 | Ghi.6224.2.S1_s_at | CO491443 | -3.43 | -18.27 | -3.77 | -36.54 | ATLP-1 (Arabidopsis thaumatin-like protein 1) |
| 71 | Ghi.8636.2.S1_s_at | DT054289 | -8.96 | -39.09 | -4.78 | -68.17 | weakly similar to NP_001058178.1 Os06g0643500 |
| 72 | GhiAffx.60102.1.S1_at | DW502706.1 | -3.12 | -18.31 | -3.27 | -29.78 | thymidine kinase |
| 73 | Ghi.6824.1.A1_s_at | CA993003 | -4.76 | -18.70 | -3.89 | -3.29 | Transcribed locus |
| 74 | GhiAffx.53387.1.S1_s_at | DW229010.1 | -5.93 | -27.70 | -4.41 | -8.28 | weakly similar to NP_001048288.1 Os02g0776900 |
| 75 | Ghi.9662.1.S1_s_at | DR459454 | -5.01 | -16.84 | -4.35 | -7.04 | TSO2 (TSO2); ribonucleoside-diphosphate reductase |
| 76 | GhiAffx.7769.1.A1_s_at | DW515515.1 | -5.01 | -17.69 | -3.09 | -7.20 | hydroxyproline-rich glycoprotein family protein |
| 77 | Ghi.8742.1.A1_at | DT052852 | -3.41 | -31.86 | -3.93 | -27.76 | basic helix-loop-helix (bHLH) family protein |
| 78 | Ghi.4436.1.A1_at | DT053396 | -3.73 | -21.70 | -4.02 | -11.04 | weakly similar to NP_001048288.1 Os02g0776900 |
|  |  |  |  |  |  |  |  |
| 79 | Ghi.4351.1.S1_s_at | AY464058.1 | -3.53 | -24.10 | -3.37 | -14.56 | (CYCLIN D3;1); cyclin-dependent protein kinase regulator |
| 80 | GhiAffx.6753.1.S1_x_at | DT049517 | -4.13 | -42.80 | -5.36 | -27.59 | universal stress protein (USP) family protein |
| 81 | GraAffx.1940.1.S1_s_at | CO123277 | -3.31 | -45.67 | -3.41 | -18.58 | Unknown |
| 82 | GraAffx.15749.1.A1_s_at | CO110143 | -4.07 | -13.84 | -15.67 | -4.03 | Unknown |
| 83 | Gra.1312.2.S1_s_at | CO085934 | -5.86 | -5.44 | -5.30 | -3.04 | SCR (SCARECROW); transcription factor |
