# Supplementary material for: Genome‐wide transcriptomic and proteomic analyses of bollworm‐infested developing cotton bolls revealed the genes and pathways involved in the insect pest defence mechanism
Source: Plant Biotechnol J. 2016 Jan 22;14(6):1438–55. doi: 10.1111/pbi.12508 (PMC5066800; doi:10.1111/pbi.12508)
Supplement: Supplementary file 7 — Table S6 List of qRT‐PCR genes and primers used in this study. [file PBI-14-1438-s003.doc]

| **Supporting table S6** List of qRT-PCR genes and primers used in this study | | | | | | |
| --- | --- | --- | --- | --- | --- | --- |
| **S.**  **No** | **Probeset ID** | **Accession No.** | **Annotation** | **Forward primer (5’-3’)** | **Reverse primer (5’-3’)** | **Amplicon**  **Size**  **(bp)** |
| **Up-regulated genes** | | | | | | |
| 1 | Ghi.3743.1.A1_at | DT462103 | Transcribed locus | AATATACCGGCGGACAAAGAG | CCAATTCTTCTGCAATGTCGTATT | 103 |
| 2 | GhiAffx.61299.1.S1_at | DW508705 | Kunitz family protein | TAACAACCGGTGGAGTTGAAG | GGCAGTAAACAAGCCTGTAGT | 99 |
| 3 | Ghi.7933.1.S1_at | AF410458 | Polygalacturonase | TTTCACCACCTTGCTCATCTC | CGATGCGTTTGGCAACAAATA | 123 |
| 4 | Ghi.7950.1.S1_at | AY366083 | POD9 precursor (pod9) | CATGGACCGACTCTACAACTTC | GCCTTTAGGACATCGTTGTTTG | 100 |
| 5 | GhiAffx.25508.1.S1_at | DW496030 | Predicted protein | GATGAGAAAGGAAACGGCAAAC | CAGAGGAAGAGTTTAGAGAGCTAATG | 120 |
| 6 | GhiAffx.19697.1.A1_s_at | DW227913 | Unknown protein | CCATCTCATCATCCTTCTTCTTCT | GTTGGGTGAGCGGCTATT | 104 |
| 7 | Ghi.9150.1.S1_at | DT463688 | Transcribed locus | AGGAGTTTCCATTTATGTGCTTTC | GAGGAGGAAATGGTAGGATCTTG | 141 |
| 8 | Ghi.23.4.A1_at | DT466530 | ACS1 (ACC synthase 1) | CATGAGGCTCTCGTCGTATAAC | TCATGAAGCAGACAAGTCTCAA | 100 |
| 9 | GhiAffx.24975.1.A1_at | DW513882 | Unknown | CGAGAAGTACCGATCATGGAAG | CCCAAAGTTGTTAATGGAGAAGTG | 126 |
| 10 | Ghi.6429.1.S1_at | CF932111 | Transcribed locus | CTACTTGTCCCTACAAGGTCAC | GAAACTCTACATAACACGCTTGC | 101 |
| 11 | Ghi.9102.1.S1_at | DV849822 | Unknown | GCTTCTTCATCGTTGGCAATAC | CTAGAACCGTCCTTGTCTCATTT | 110 |
| 12 | GhiAffx.8694.1.S1_a_at | DW499451 | DW499451.1 | AGGGCTGGATTGTGAGTAATG | GCTTCTCGATTACCAGGAAGAA | 113 |
| 13 | Ghi.1011.1.A1_s_at | DT463974 | YLS9 (Yellow-leaf-specific gene 9) | CCCACCAAGGATAACCAGATG | GATCGAGGCTAGGGCTTACTA | 133 |
| 14 | Ghi.10595.1.S1_at | DN761009 | Unknown protein | GAACAATGAAGAGATTGACCATGAA | CCTAGCAAGCACAGCATTAGA | 100 |
| 15 | Ghi.6656.1.A1_at | CA993777 | Transcribed locus | ACACTGAACCTACCGAAATCAA | TCATCTTCAAGCTTTCCCATACT | 114 |
| 16 | GhiAffx.4130.1.S1_at | DW498401 | Zinc finger (C3HC4-type RING finger) family protein | TGTGTTTCATTCGGGTTGTATTG | TACCCTCACCACCTCCTATTT | 110 |
| 17 | Ghi.9280.3.A1_at | DT462248 | Unknown | GCCTTCCCAGGTTTCTACTT | CGTTTAGCTCTTGGCTGTTATG | 104 |
| 18 | Ghi.2608.2.A1_at | DT463212 | Unknown protein | GAGTTGCTTTGGCATTCTCATT | ACTCCCAAACATCAACACTACA | 117 |
| 19 | Ghi.7907.1.S1_s_at | AI055500 | RD26 (Responsive to dessication 26) | AAGTCTGGGTATCGGGAATTTG | CCCTGACTCTGAGTTTGAGTTT | 106 |
| 20 | GhiAffx.7921.1.S1_at | DW517203 | Transcribed locus | CTTCGGAACTTAGGATAGGGATTT | AGCAAGATCGGTGAGTGTTT | 102 |
| 21 | Ghi.1069.1.S1_x_at | DN759630 | ATBCB (Arabidopsis blue-copper-binding protein) | CCATTGCCTTAGCTTTGTTCTG | ACGAATGAAGACTGTGTCTGG | 116 |
| 22 | GhiAffx.2668.1.A1_at | DT463855 | Unknown | GAAGGTAAAGACCCTCGTAAGC | AGCAGACGGTTACACAATCAA | 101 |
| 23 | Ghi.6664.1.S1_at | CA993761 | Transcribed locus | AGCAAATACCAATCACCAATCTATG | TGAAAGTGTCGAGCTGAGAAG | 107 |
| 24 | Ghi.4.1.A1_at | CK987701 | Unknown | GAGTTAACTGCATTTCGCCTAAC | CAACACTGCTGAAACCGAAAG | 110 |
| 25 | Ghi.1008.1.S1_at | CK987666 | ATOSM34 (Osmotin 34) | CCCAAAGGATGACCCAACAA | GAAAGGACTTAAACCATCCCAAATAC | 102 |
| 26 | Ghi.2169.1.A1_at | DT468931 | GRAM domain-containing protein / ABA-responsive protein-related | GCGATCACTACAGAAAGCTACA | CGCAACGTATTCAAACGAGAAG | 121 |
| 27 | Ghi.3328.1.A1_at | DT463239 | Cytochrome P450 | CCACCATTCGTTGAGCTAGA | AGCCTGAGAGATTTGATGGTAATA | 114 |
| 28 | Ghi.7891.1.S1_s_at | DT462224 | Extracellular dermal glycoprotein / EDGP | ACTTCAAGGCTGGGATTCAG | CGAGGGCTACGACGTTAAAT | 101 |
| 29 | Ghi.9152.1.S1_at | DT462541 | Transducin family protein / WD-40 repeat family protein | GTCATACCGGTCCAGTCAAAT | TTATCGAGGCTACCACTGTAAAC | 100 |
| 30 | GhiAffx.12729.1.A1_at | DT466204 | Protein kinase family protein | GACCGTAACCGGGATGTTT | GGGTTGGCAAAGCTTCTTATG | 108 |
| 31 | Ghi.8476.1.S1_x_at | CO495095 | Transcribed locus | CAACGCAATCGTGACTCATAAC | CATAGTGCATGCATGTGTTTCT | 124 |
| 32 | GhiAffx.15571.1.S1_a_at | DW505599 | Unknown | GACAGCCTGAGATGATGAAGAG | ACAAACCTAGTGACCAACAGAG | 113 |
| **Down-regulated genes** | | | | | | |
| 1 | Ghi.8826.1.S1_s_at | DT566192 | ATLP-1 (Arabidopsis thaumatin-like protein 1) | TCTTCGTATACTAGATGTCGCTTTC | CGCCACCCACACAGATATT | 139 |
| 2 | Ghi.5081.1.S1_s_at | DT049160 | Glyoxal oxidase-related | TCACCTGTAAGTCACCACTTG | GGAGGCATTCTATCCGGTTATT | 131 |
| 3 | Ghi.5267.1.A1_at | DT046626 | GBF6 (G-box binding factor 6) | ACAGAGTTCTCAGCTTCAATGT | GGCTCAACTCACACTCCTAAG | 99 |
| 4 | Gra.2938.2.A1_at | CO085472 | THI1 (Thiazole requiring) | ACGCTATCGATCATCCCAATAC | CAATCTTGCATGGACCCAAAC | 122 |
| 5 | Ghi.8520.1.S1_at | DR044160 | Delta-8 sphingolipid desaturase | GTGTTCGTCTTGGATGGATTTG | TTGTCCGTAAATGGCACCT | 96 |
| 6 | Ghi.10316.1.S1_s_at | DT051688 | Phosphate-responsive 1 family protein | CCGCCTGAACCGTACAAA | GCTAGCTGAATCGTCGAGTAAT | 111 |
| 7 | Ghi.6188.1.A1_at | CO493635 | ATEXPA4 (Arabidopsis thaliana expansin A4) | TCGTGAAGGCTAGTGTGAAAG | ACTGCCTGTGACCCTAAATG | 125 |
| 8 | Ghi.8636.2.S1_s_at | DT054289 | Unknown | CCGGTTGTTCCTCCAATAGTT | TTTCCCACTTGGTGGCTTAG | 114 |
| 9 | Ghi.10609.1.S1_s_at | DN761916 | Polygalacturonase-inhibiting protein (PGIPL) | ACTCCCTTGAAGGCAACATAC | CGAACTTCGCCGATGATAGAG | 120 |
| 10 | GhiAffx.6042.1.S1_s_at | DW498160 | Gibberellin-regulated family protein | GTTACAGGGACCTGAAGAACTC | ATGGGTCCTGATGTTTAGGAAA | 104 |
