# Supplementary material for: Genome‐wide transcriptomic and proteomic analyses of bollworm‐infested developing cotton bolls revealed the genes and pathways involved in the insect pest defence mechanism
Source: Plant Biotechnol J. 2016 Jan 22;14(6):1438–55. doi: 10.1111/pbi.12508 (PMC5066800; doi:10.1111/pbi.12508)
Supplement: Supplementary file 8 — Table S7 Expression pattern of transcripts related to cell wall, cell division and cell growth. [file PBI-14-1438-s004.doc]

| **Supporting table S7** Expression pattern of transcripts related to cell wall, cell division and cell growth | | | | | | | |
| --- | --- | --- | --- | --- | --- | --- | --- |
| **S. No** | **Probeset ID** | **Accession No** | **Gene name** | **Boll developmental stages (dpa)** | | | |
|  |  |  |  | **0** | **2** | **5** | **10** |
| 1 | Ghi.4532.1.A1_at | DR457588 | XTR4 (Xyloglucan endotransglycosylase 4); Hydrolase, Acting on glycosyl bonds | **+** |  | **+** | **+** |
| 2 | GhiAffx.63628.1.S1_at | DW499687.1 | EXGT-A1 (Endo-xyloglucan transferase); Hydrolase, Acting on glycosyl bonds | **+** | **-** |  | **+/-** |
| 3 | Ghi.175.1.S1_at | DR462047 | Xyloglucan:xyloglucosyl transferase, putative / Xyloglucan endotransglycosylase/ Endo-xyloglucan transferase | **+/-** | **-** | **+/-** | **+/-** |
| 4 | Ghi.5068.1.S1_at | AW587517 | XTR2 (Xyloglucan endotransglycosylase related 2); Hydrolase, Acting on glycosyl bonds |  |  | **+** | **+** |
| 5 | Ghi.5744.2.S1_s_at | DN760209 | XTH9 (Xyloglucan endotransglucosylase/Hydrolase 9); Hydrolase, Acting on glycosyl bonds |  | **-** | **-** | **-** |
| 6 | Ghi.6236.1.S1_s_at | AY189971.1 | Endo-xyloglucan transferase, clone CF101 | **-** | **-** |  | **-** |
| 7 | Ghi.687.1.S1_at | DQ060250.1 | Expansin (Exp) |  | **-** | **-** | **-** |
| 8 | Ghi.2039.1.S1_x_at | AY189969.1 | Alpha-expansin 1 |  |  | **-** | **-** |
| 9 | Ghi.249.1.A1_at | DQ204496.1 | Alpha-expansin 2 |  |  | **+** | **-** |
| 10 | Ghi.6188.1.A1_at | CO493635 | ATEXPA4 (*Arabidopsis thaliana* expansin A4) | **+/-** | **+/-** | **-** | **-** |
| 11 | Ghi.9840.2.S1_s_at | DT566253 | ATEXPA6 (*Arabidopsis thaliana* expansin A6) |  |  |  | **-** |
| 12 | GhiAffx.14333.1.S1_s_at | CD486517 | ATEXPA8 (*Arabidopsis thaliana* expansin A8) | **+** | **+** | **+** | **-** |
| 13 | GhiAffx.6239.1.S1_at | DW498116.1 | ATEXPA10 (*Arabidopsis thaliana* expansin A10) | **-** | **-** |  | **-** |
| 14 | Ghi.9930.1.S1_s_at | AI728493 | ATEXPA13 (*Arabidopsis thaliana* expansin A13) |  |  | **-** | **-** |
| 15 | Gra.3004.2.S1_s_at | CO086920 | ATEXPA15 (*Arabidopsis thaliana* expansin A15) | **-** | **-** |  | **-** |
| 16 | Ghi.9.2.A1_at | DR463856 | ATEXLA3 (*Arabidopsis thaliana* expansin-like A3) |  |  | **+** | **+** |
| 17 | Ghi.6465.2.S1_at | CD485906 | ATEXLB1 (*Arabidopsis thaliana* expansin-like B1) | **+** | **+** | **+** | **+** |
| 18 | GhiAffx.29477.1.S1_at | DV850143 | Alpha-galactosidase/Melibiase/Alpha-D-galactoside galactohydrolase |  |  |  | **+** |
| 19 | Gra.2314.1.S1_at | CO126415 | Beta-galactosidase | **+** |  | **+** | **+** |
| 20 | Ghi.5366.1.S1_x_at | DT048057 | Beta-galactosidase / Lactase |  |  | **+** | **+/-** |
| 21 | Ghi.7994.3.S1_s_at | DT466759 | BGAL1 (Beta galactosidase 1); Beta-galactosidase | **+** |  | **+** | **+** |
| 22 | Ghi.669.2.A1_s_at | DT463066 | BGAL3 (Beta-galactosidase 3) | **-** |  |  | **-** |
| 23 | Ghi.3168.2.A1_s_at | DR463922 | BGAL10 (Beta-galactosidase 10) |  |  |  | **-** |
| 24 | Gra.2459.3.S1_s_at | CO128079 | BGAL12; Beta-galactosidase | **+** |  |  | **+** |
| 25 | Ghi.8147.1.S1_at | DQ073046.1 | Pectate lyase | **+** |  | **-** | **-** |
| 26 | Ghi.10256.1.S1_at | DT567984 | Pectate lyase family protein |  | **-** |  | **-** |
| 27 | Ghi.3458.1.A1_at | DT465599 | Pectinesterase family protein | **+/-** | **+/-** | **+** | **+/-** |
| 28 | Ghi.9782.1.S1_s_at | AI729755 | Pectinacetylesterase |  |  | **+** | **+/-** |
| 29 | Ghi.2406.1.A1_s_at | DT052156 | ATPME3 (*Arabidopsis thaliana* pectin methylesterase 3) |  | **-** |  | **-** |
| 30 | GhiAffx.53058.1.S1_s_at | DW518780.1 | Invertase/Pectin methylesterase inhibitor family protein | **+/-** | **-** | **+** | **-** |
| 31 | Gra.2842.4.A1_a_at | CO083478 | Enzyme inhibitor/ Pectinesterase/ Pectinesterase inhibitor | **+** | **+** |  | **-** |
| 32 | Ghi.1552.1.S1_s_at | DN779868 | C/VIF1 (Cell wall / Vacuolar inhibitor of fructosidase 1); Pectinesterase inhibitor | **+** |  |  |  |
| 33 | GhiAffx.52877.1.A1_at | DW224606.1 | Glycoside hydrolase family 28 protein / Polygalacturonase (Pectinase) family protein | **-** | **-** | **-** | **-** |
| 34 | GhiAffx.30838.1.S1_at | DW504875.1 | Polygalacturonase/ Pectinase | **-** | **-** |  | **-** |
| 35 | Ghi.3263.1.A1_at | DT467895 | ATCSLG2 (Cellulose synthase-like G2); Transferase/ Transferase, Transferring glycosyl groups | **+** | **+** | **+** | **+** |
| 36 | Ghi.1449.1.S1_s_at | DN780764 | ATCSLE1 (Cellulose synthase-like E1); Cellulose synthase/ Transferase, Transferring glycosyl groups |  |  | **+** | **+** |
| 37 | Ghi.4648.3.A1_x_at | CO493453 | CESA8 (Cellulase synthase 8); Cellulose synthase/ Transferase, Transferring glycosyl groups |  |  |  | **-** |
| 38 | Ghi.7294.1.S1_s_at | AI731843 | ATCSLA02 (Cellulose synthase-like A2); Transferase, Transferring glycosyl groups |  |  |  | **-** |
| 39 | Ghi.8518.1.A1_at | DT048349 | IRX3 (Irregular xylem 3, Murus 10); Cellulose synthase |  |  |  | **-** |
| 40 | Ghi.1151.1.A1_at | U58283.1 | Cellulose synthase |  |  |  | **-** |
| 41 | GhiAffx.21602.1.A1_at | DW496092.1 | ATCSLC05 (Cellulose synthase-like C5); Transferase, Transferring glycosyl groups |  |  |  | **-** |
| 42 | Ghi.6061.1.S1_at | DT047912 | Cellulose synthase (celA2) |  |  |  |  |
| 43 | Ghi.5750.1.S1_s_at | AY218846.1 | Arabinogalactan protein 2 | **-** |  | **-** | **-** |
| 44 | GhiAffx.8371.1.S1_at | DW225918.1 | Arabinogalactan protein 3 |  |  | **+** | **-** |
| 45 | GhiAffx.62441.1.S1_at | DW501939.1 | Phytocyanin-like arabinogalactan-protein (PLA1) |  | **-** |  | **-** |
| 46 | Ghi.8186.1.S1_s_at | AI726859 | Fasciclin-like arabinogalactan protein 2 (FLA2) |  |  | **-** | **-** |
| 47 | GhiAffx.13672.1.S1_s_at | DT050706 | Fasciclin-like arabinogalactan protein 3 (FLA3) |  | **-** | **-** | **-** |
| 48 | Ghi.8612.2.A1_a_at | DT047433 | Fasciclin-like arabinogalactan protein 5 (FLA5) /// Arabinogalactan protein 4 |  |  |  | **-** |
| 49 | Ghi.4418.1.S1_s_at | DR176775 | Fasciclin-like arabinogalactan protein 6 (FLA6) |  |  |  | **-** |
| 50 | Ghi.2198.1.S1_s_at | DT550268 | Fasciclin-like arabinogalactan protein 11 (FLA11) | **-** | **-** | **-** | **-** |
| 51 | Ghi.4140.1.S1_s_at | DT462762 | Fasciclin-like arabinogalactan protein 12 (FLA12) |  |  |  | **-** |
| 52 | Ghi.9666.1.S1_at | DN759995 | Fasciclin-like arabinogalactan protein 13 (FLA13) |  | **-** | **-** | **-** |
| 53 | GhiAffx.16299.1.S1_s_at | DW229097.1 | Fasciclin-like arabinogalactan protein 15 (FLA15) |  |  |  | **-** |
| 54 | Ghi.3595.1.S1_s_at | DT465477 | Fasciclin-like arabinogalactan protein 16 (FLA16) |  |  |  | **-** |
| 55 | GhiAffx.6110.1.A1_x_at | DW226506.1 | Fasciclin-like arabinogalactan protein 19 (FLA19) |  |  | **-** | **-** |
| 56 | Ghi.5694.1.A1_x_at | DT046743 | Glycine/proline-rich protein |  | **+** |  | **+** |
| 57 | Ghi.9405.1.A1_at | DT463935 | Hydroxyproline-rich glycoprotein family protein | **+/-** | **+/-** | **+/-** | **+/-** |
| 58 | Gra.390.1.S1_s_at | CO072031 | ATPRP1 (Proline-rich protein 1); Structural constituent of cell wall |  | **+** |  | **+** |
| 59 | Ghi.783.1.S1_s_at | L17308.1 | Proline-rich cell wall protein |  |  | **-** | **-** |
| 60 | Ghi.8461.1.A1_at | CO499660 | Proline-rich protein (PRP3) |  |  |  | **-** |
| 61 | Ghi.5930.1.S1_s_at | AI730672 | PRP4 (Proline-rich protein 4) |  |  |  | **-** |
| 62 | GhiAffx.10687.1.S1_at | DW496168.1 | Proline-rich family protein |  |  | **-** | **-** |
| 63 | Ghi.1314.3.A1_x_at | CO491190 | Alpha tubulin 1 |  |  | **-** | **-** |
| 64 | Ghi.5746.1.S1_s_at | AY345604.1 | Alpha-tubulin (TubA2) |  | **-** | **-** | **-** |
| 65 | Ghi.6667.1.A1_s_at | DR463669 | Xu-142 alpha-tubulin 3 (ATub3) |  | **-** |  | **-** |
| 66 | Ghi.7832.1.S1_at | DT573045 | Alpha-tubulin (TUA5) |  |  |  | **-** |
| 67 | Ghi.6298.1.A1_s_at | CD486518 | Alpha-tubulin (TUA6) |  | **-** |  | **-** |
| 68 | Ghi.4663.1.A1_x_at | DT051323 | Alpha-tubulin (TUA7) | **-** | **-** | **-** | **-** |
| 69 | Ghi.1314.2.S1_s_at | AY345603.1 | Alpha-tubulin (TUA7) /// Alpha-tubulin 1 |  |  | **-** | **-** |
| 70 | Ghi.1314.1.S1_x_at | AY345605.1 | Alpha-tubulin (TUA10) |  | **-** |  | **-** |
| 71 | Ghi.4617.1.A1_at | DT048631 | Alpha-tubulin (TUA12) |  |  |  |  |
| 72 | Ghi.8448.1.S1_x_at | AF521240.1 | Xu-142 beta-tubulin 1 (BTub1) | **-** |  | **-** | **-** |
| 73 | Gra.2522.2.A1_x_at | CO127919 | Beta tubulin 2 | **-** |  |  | **-** |
| 74 | GhiAffx.42226.1.A1_s_at | DW507015.1 | Beta-tubulin 4 | **-** | **-** |  | **-** |
| 75 | Ghi.4629.1.S1_x_at | AY345606.1 | TUB6 (Beta-6 tubulin) | **-** |  | **-** | **-** |
| 76 | Ghi.1893.1.S1_at | DV849529 | Beta-tubulin 8 | **-** | **-** |  | **-** |
| 77 | GhiAffx.20995.1.A1_s_at | DW234589.1 | TUB8 (tubulin beta-8) |  |  |  | **-** |
| 78 | Ghi.9969.2.S1_s_at | DT557030 | Beta-tubulin 10 |  |  |  | **-** |
| 79 | Ghi.8096.1.S1_x_at | AY345608.1 | Beta-tubulin 11 |  |  |  | **-** |
| 80 | Ghi.9707.1.S1_x_at | DT051791 | Beta-tubulin 15 |  |  |  | **-** |
| 81 | Ghi.9446.1.S1_at | DT047992 | Beta-tubulin 17 |  |  | **-** | **-** |
| 82 | Ghi.10095.1.S1_x_at | DT465449 | Beta-tubulin 18 |  | **-** | **-** | **-** |
| 83 | GhiAffx.24936.1.S1_at | DW503093.1 | TUBG1 (Gamma-tubulin); Structural molecule |  | **-** |  |  |
| 84 | Ghi.7355.3.S1_s_at | DT554367 | Tubulin folding cofactor B |  |  |  | **-** |
| 85 | Ghi.7983.1.S1_s_at | AI729399 | ACT3 (Actin 3); Structural constituent of cytoskeleton |  | **-** | **-** | **-** |
| 86 | Ghi.4376.1.S1_x_at | AY305726.1 | Actin (ACT4) |  |  |  | **-** |
| 87 | Ghi.5849.1.S1_s_at | DR454104 | ACT7 (actin 7) |  |  |  | **-** |
| 88 | Ghi.1927.1.S1_at | DV849072 | ACT11 (Actin-11); Structural constituent of cytoskeleton |  | **-** | **-** | **-** |
| 89 | Ghi.468.1.A1_s_at | AY189970.1 | Profilin |  |  |  | **-** |
| 90 | Ghi.5050.1.S1_s_at | DT049289 | ADF1 (Actin depolymerizing factor 1) |  |  | **-** | **-** |
| 91 | Ghi.6590.1.S1_s_at | AI728226 | F-actin capping protein alpha subunit family protein |  | **-** | **-** | **-** |
| 92 | Ghi.8872.1.S1_s_at | DT456420 | Actin 87E CG18290-PA, isoform A |  |  |  | **+** |
| 93 | Ghi.301.1.S1_at | DR461064 | HAF01 (Histone acetyltransferase TAFII250 family); DNA binding |  |  |  | **+** |
| 94 | GhiAffx.16588.1.A1_s_at | DW227332.1 | Histone H2A |  | **-** | **-** | **-** |
| 95 | GhiAffx.1589.47.A1_s_at | DW224080.1 | Histone H2B |  | **-** | **-** | **-** |
| 96 | GhiAffx.20386.1.S1_at | DW514241.1 | Histone H2A.F/Z |  | **-** | **-** | **-** |
| 97 | Ghi.5452.1.A1_s_at | DT047693 | Histone family member (his-35) |  | **-** |  | **-** |
| 98 | GhiAffx.26202.1.S1_at | DW520588.1 | Histone H4 replacement CG3379-PC, isoform C |  | **-** |  | **-** |
| 99 | GhiAffx.13316.3.S1_at | DT527189 | NRP1 (NAP1-related protein 1); DNA binding / Chromatin binding / Histone binding |  | **-** | **-** | **-** |
| 100 | GhiAffx.879.1.S1_at | DN816995 | HDA14 (Histone deacetylase 14); Histone deacetylase | **-** | **-** | **-** | **-** |
| 101 | Ghi.6423.1.A1_at | CO490711 | ATAUR1 (Ataurora1); Histone serine kinase(H3-S10 specific) |  | **-** |  | **-** |
| 102 | GhiAffx.1397.1.S1_s_at | DW229025.1 | Histone H1/H5 family protein |  |  |  | **-** |
| 103 | GraAffx.18009.2.S1_s_at | CO124410 | Histone deacetylase 2 isoform b |  | **-** |  |  |
| 104 | GhiAffx.29424.1.A1_s_at | DW237302.1 | Minichromosome maintenance family protein / MCM family protein |  | **-** | **-** | **-** |
| 105 | GhiAffx.113.1.S1_s_at | DW237468.1 | PRL (Prolifera); ATP binding / DNA binding / DNA-dependent ATPase |  | **-** | **-** | **-** |
| 106 | Ghi.3.1.A1_x_at | DT048978 | PCNA2 (Proliferating cell nuclear 2); DNA binding / DNA polymerase processivity factor |  | **-** |  | **-** |
| 107 | GhiAffx.20319.1.S1_s_at | DW493277.1 | CYCP3;1 (cyclin p3;1); Cyclin-dependent protein kinase |  | **-** | **-** | **-** |
| 108 | Gra.2137.1.S1_x_at | CO127702 | CYCD3;2; Cyclin-dependent protein kinase |  | **-** |  | **-** |
| 109 | Ghi.4351.1.S1_s_at | AY464058.1 | CYCD3;1 (Cyclin D3;1); Cyclin-dependent protein kinase regulator | **-** | **-** | **-** | **-** |
| 110 | Ghi.10092.1.S1_at | DT053988 | CYC1BAT (Cyclin B 1;2); Cyclin-dependent protein kinase regulator |  | **-** |  | **-** |
| 111 | GhiAffx.25902.1.A1_at | DW518947.1 | CYCB1;4 (Cyclin 3); Cyclin-dependent protein kinase regulator |  | **-** |  | **-** |
| 112 | GhiAffx.40752.1.S1_s_at | DW499331.1 | ATCDT1A/CDT1/CDT1A (*Arabidopsis* homolog of yeast CDT1 A); Cyclin-dependent protein kinase/ Protein binding |  | **-** | **-** | **-** |
| 113 | Ghi.8534.1.A1_s_at | DT051720 | CYCP3;2 (Cyclin p3;2); Cyclin-dependent protein kinase |  | - |  | - |
| 114 | Ghi.927.2.S1_at | AI728683 | CYCD4;1 (Cyclin D4;1); Cyclin-dependent protein kinase regulator |  |  |  | **-** |
| 115 | Ghi.2844.1.S1_s_at | DT546186 | CYCH;1 (Cyclin H;1); Cyclin-dependent protein kinase/ Protein binding / Protein kinas |  |  |  | **-** |
| 116 | GhiAffx.15205.2.S1_at | DW493769.1 | CDC2/CDC2A/CDC2AAT/CDK2/CDKA;1 (Cell division control 2); Cyclin-dependent protein kinase/ Kinase/ Protein binding |  |  |  | **-** |

(**+**) indicates up-regulated transcripts

(**-**) indicates down-regulated transcripts

(+/-) indicates differentially regulated transcripts
