# Supplementary material for: Genome‐wide transcriptomic and proteomic analyses of bollworm‐infested developing cotton bolls revealed the genes and pathways involved in the insect pest defence mechanism
Source: Plant Biotechnol J. 2016 Jan 22;14(6):1438–55. doi: 10.1111/pbi.12508 (PMC5066800; doi:10.1111/pbi.12508)
Supplement: Supplementary file 9 — Table S8 Expression pattern of transcripts related to photosynthesis. [file PBI-14-1438-s005.doc]

| **Supporting table S8** Expression pattern of transcripts related to photosynthesis | | | | | | | |
| --- | --- | --- | --- | --- | --- | --- | --- |
| **S.No** | **Probeset ID** | **Accession No** | **Gene Name** | **Boll developmental stages (dpa)** | | | |
|  |  |  |  | **0** | **2** | **5** | **10** |
| 1 | Ghi.4037.3.S1_s_at | DT567171 | ATP synthase protein I -related | - |  |  |  |
| 2 | Ghi.8709.1.A1_at | DR461016 | ATP synthase beta chain 1 |  | - |  | - |
| 3 | GhiAffx.6895.1.S1_s_at | DW505850.1 | ATP synthase gamma chain 2 |  | - |  | - |
| 4 | Ghi.1161.1.S1_s_at | DT047487 | ATP synthase delta chain, chloroplast / H(+)-transporting two-sector ATPase, delta (OSCP) subunit |  |  |  | - |
| 5 | GhiAffx.17920.1.S1_s_at | DW481817.1 | Mitochondrial ATP synthase g subunit family protein |  |  |  | - |
| 6 | Ghi.7554.1.S1_at | AI729453 | (Vacuolar ATP synthase subunit D); Hydrogen ion transporting ATP synthase, rotational mechanism / Hydrogen ion transporting ATPase, rotational mechanism |  |  |  | - |
| 7 | GhiAffx.7848.1.S1_at | DW493614.1 | ATP synthase CF1 beta subunit |  |  | **+** |  |
| 8 | Ghi.7632.1.S1_s_at | AI725673 | (Vacuolar ATP synthase subunit B2); Hydrogen ion transporting ATP synthase, rotational mechanism |  |  |  | **+** |
| 9 | Ghi.1128.1.S1_at | DN779673 | Photosystem I reaction center subunit VI, chloroplast/ PSI-H, putative (PSAH2) |  | - |  | - |
| 10 | Ghi.6581.1.S1_x_at | DN780965 | LHCB4.3 (Light harvesting complex PSII); Chlorophyll binding | - | - | - | - |
| 11 | Ghi.88.1.S1_s_at | DV848673 | LHCB5 (Light harvesting complex of photosystem II 5); Chlorophyll binding |  | - |  | - |
| 12 | Ghi.1146.1.S1_a_at | DN780642 | LHCB2.1 (Photosystem II light harvesting complex gene 2.1); Chlorophyll binding |  | - |  | - |
| 13 | Ghi.1400.1.S1_s_at | CD486214 | LHCA4 (Photosystem I light harvesting complex gene 4); Chlorophyll binding |  | - |  | - |
| 14 | GhiAffx.17581.1.S1_at | DW518594.1 | LHCA5 (Photosystem I light harvesting complex gene 5) |  |  |  | - |
| 15 | Ghi.6780.1.S1_s_at | CA993199 | CAB1 (Chlorophyll A/B binding protein 1); Chlorophyll binding | **+** | - | **+** | **+/**- |
| 16 | Gra.2939.2.S1_s_at | CO088487 | LHCB4.3; Chlorophyll binding |  | - |  |  |
| 17 | Ghi.9473.2.S1_s_at | AI727241 | CUE1 (CAB underexpressed 1); Antiporter/ Triose-phosphate transporter |  |  |  | - |
| 18 | GhiAffx.59768.1.S1_at | DW235537.1 | SUB1 (Short under blue light 1) |  |  |  |  |
| 19 | Ghi.1949.1.S1_s_at | DV848790 | ATFD1 (Ferredoxin 1); Electron carrier/ Iron ion binding |  | - |  | - |
| 20 | Ghi.7727.1.S1_s_at | AI727496 | ATFD3 (Ferredoxin 3); Electron carrier |  |  |  | - |
| 21 | GhiAffx.29182.1.S1_at | DW511527.1 | Apoferredoxin |  | - |  | - |
| 22 | Ghi.6599.1.A1_at | DT462996 | SIR (Sulfite reductase); Sulfite reductase (Ferredoxin) |  |  |  | - |
| 23 | GhiAffx.20889.1.S1_at | DW233795.1 | FED A (Ferredoxin 2); Electron carrier/ Iron ion binding | **+** |  |  |  |
| 24 | Ghi.3170.2.A1_at | DT468829 | NIR1 (Nitrite reductase); Ferredoxin-nitrate reductase |  |  | **+** |  |
| 25 | Ghi.1574.1.S1_at | DV849347 | FTRA2 (Ferredoxin/Thioredoxin reductase subunit A (variable subunit) 2); Ferredoxin:thioredoxin reductase |  | **+** |  |  |
| 26 | GhiAffx.22444.1.S1_at | DW500755.1 | Plastocyanin-like domain-containing protein | - | - | - | - |
| 27 | Gra.314.1.S1_s_at | CO088579 | Plastoquinol-plastocyanin reductase |  | - |  | - |
| 28 | Ghi.9252.1.A1_at | DT467015 | Chloroplast thylakoid processing peptidase | **+** |  |  |  |
| 29 | Ghi.6041.1.S1_s_at | DV849896 | Chloroplast nucleoid DNA-binding protein |  |  | **+** |  |
| 30 | Ghi.8767.1.S1_s_at | DT543374 | Chloroplast nucleotide DNA-binding protein related | - | - |  | - |
| 31 | Ghi.1049.1.S1_at | DN802307 | Chloroplast ADP, ATP carrier protein 1 / ADP, ATP translocase 1 / Adenine nucleotide translocase 1 (AATP1) |  | - | **+** | - |
| 32 | GhiAffx.25090.1.S1_at | DW484578.1 | Chloroplast lumen common family protein |  | - |  | - |
| 33 | GhiAffx.36723.1.S1_at | DW515956.1 | Chloroplast thylakoid lumen protein |  | - |  | - |
| 34 | Ghi.6128.1.S1_s_at | DR453882 | Chloroplast biotin carboxylase (BC1) mRNA; Nuclear gene for chloroplast product |  |  |  | - |
| 35 | Ghi.1378.1.S1_at | CA992997 | 29 kDa ribonucleoprotein, chloroplast/ RNA-binding protein cp29 |  |  | - | - |
| 36 | GhiAffx.911.1.S1_s_at | DW517325.1 | 50S ribosomal protein L21, chloroplast / CL21 (RPL21) |  |  |  | - |
| 37 | GhiAffx.17656.1.S1_a_at | AJ513179 | 50S ribosomal protein L28, chloroplast (CL28) |  |  |  | - |
| 38 | Ghi.10678.1.S1_s_at | DR458501 | Chloroplast chaperonin 10 |  |  |  | - |
| 39 | Ghi.9564.1.S1_s_at | DR458096 | CPN60A (Chloroplast / 60 kDa chaperonin alpha subunit); ATP binding / Protein binding / Unfolded protein binding |  |  |  | - |
| 40 | Ghi.10340.1.S1_at | DV849275 | 2-cys peroxiredoxin, Chloroplast |  |  |  | - |
| 41 | Ghi.8153.1.S1_s_at | DQ120514.1 | Chloroplast Cu/Zn superoxide dismutase mRNA; nuclear gene for chloroplast product |  |  |  | - |
| 42 | GhiAffx.19167.1.S1_s_at | DW487864.1 | MIND (Accumulation and replication of chloroplast 11) |  |  |  | - |

(**+**) indicates up-regulated transcripts

(**-**) indicates down-regulated transcripts

(+/-) indicates differentially regulated transcripts
