# Supplementary material for: Genome‐wide transcriptomic and proteomic analyses of bollworm‐infested developing cotton bolls revealed the genes and pathways involved in the insect pest defence mechanism
Source: Plant Biotechnol J. 2016 Jan 22;14(6):1438–55. doi: 10.1111/pbi.12508 (PMC5066800; doi:10.1111/pbi.12508)
Supplement: Supplementary file 10 — Table S9 Expression pattern of transcripts related to carbohydrate metabolism. [file PBI-14-1438-s006.doc]

| **Supporting table S9** Expression pattern of transcripts related to carbohydrate metabolism | | | | | | | |
| --- | --- | --- | --- | --- | --- | --- | --- |
| S.No | Probeset ID | Accession No | Gene name | Boll developmental stages (dpa) | | | |
|  |  |  |  | 0 | 2 | 5 | 10 |
| 1 | Ghi.8034.1.S1_s_at | AY628139.1 | Trehalose 6-phosphate synthase | **+** |  | **+** | **+** |
| 2 | Ghi.6320.2.S1_at | DT462221 | ATTPS7 (*Arabidopsis thaliana* trehalose-phosphatase /synthase 7) | **+** | **+** | **+** | **+** |
| 3 | Ghi.3451.2.A1_at | DT465672 | ATTPS11 (*Arabidopsis thaliana* trehalose phosphatase/synthase 11) | **+** | **+** | **+** | **+** |
| 4 | Ghi.3389.2.A1_s_at | DT464172 | ATTPPA (*Arabidopsis thaliana* trehalose-6-phosphate phosphatase) |  |  | **+** | **+** |
| 5 | GhiAffx.36503.1.S1_at | DW238688.1 | ATGOLS1 (*Arabidopsis thaliana* galactinol synthase 1) | **+** | **+** | **+** | **+** |
| 6 | GhiAffx.34543.1.A1_at | DW227458.1 | ATGOLS2 (*Arabidopsis thaliana* galactinol synthase 2) | **+** | **+** | **+** | **+** |
| 7 | GhiAffx.16092.1.A1_s_at | DW501210.1 | SIP1 (Seed imbibition 1-like); galactinol-sucrose galactosyltransferase/ hydrolase | **+** | **+** |  | **+** |
| 8 | Ghi.9279.2.A1_s_at | DT462929 | UGE1 (UDP-D-glucose/UDP-D-galactose 4-epimerase 1) | **+** |  | **+** | **+** |
| 9 | GhiAffx.19354.1.S1_s_at | DW488498.1 | UGE5 (UDP-D-glucose/UDP-D-galactose 4-epimerase 5) | **+** | **+** | **+** | **+** |
| 10 | Gra.2050.1.S1_s_at | CO126131 | UDP-glucose 4-epimerase |  |  | **+** | **+** |
| 11 | Ghi.6751.1.A1_s_at | CA993334 | Fructose-1,6-bisphosphatase / D-fructose-1,6-bisphosphate 1-phosphohydrolase / FBPase | **+** |  |  |  |
| 12 | GhiAffx.6764.1.S1_s_at | DW504364.1 | GAL1 (Galactose kinase 1) |  |  |  | **+** |
| 13 | GhiAffx.31355.1.S1_s_at | DT462536 | UGT73B2; UDP-glycosyltransferase |  |  | **+** | **+/-** |
| 14 | Ghi.3263.1.S1_at | DT467895 | ATCSLG2 (Cellulose synthase-like G2) | **+** | **+** | **+** | **+** |
| 15 | Ghi.1449.1.S1_s_at | DN780764 | ATCSLE1 (Cellulose synthase-like E1) |  |  | **+** | **+** |
| 16 | Ghi.8123.1.S1_at | X52305.1 | Malate synthase (EC 4.1.3.2) | **+** |  | **+** | **+** |
| 17 | Ghi.8244.1.S1_s_at | DT543347 | LTA2 (Plastid E2 subunit of pyruvate decarboxylase) |  |  |  | **-** |
| 18 | Gra.1083.1.A1_at | CO121246 | APL4; glucose-1-phosphate adenylyltransferase | **-** |  |  | **-** |
| 19 | GhiAffx.42174.1.A1_s_at | DW506784.1 | glucose-6-phosphate isomerase, cytosolic (PGIC) | **-** |  |  | **-** |
| 20 | Ghi.4828.1.S1_s_at | DT050294 | UTP--glucose-1-phosphate uridylyltransferase / UDP-glucose pyrophosphorylase / UGPase |  | **-** | **-** | **-** |
| 21 | Ghi.7861.1.S1_s_at | DV849289 | UDP-glucose 6-dehydrogenase |  |  |  | **-** |
| 22 | GhiAffx.24615.1.S1_at | DW512010.1 | RHM1/ROL1 (Rhamnose biosynthesis1); UDP-glucose 4,6-dehydratase/ catalytic |  |  |  | **-** |
| 23 | GhiAffx.34102.1.S1_s_at | DW517542.1 | PGM (Phosphoglucomutase) |  |  |  | **-** |
| 24 | GhiAffx.6228.2.A1_s_at | DW498003.1 | G6PD2 (Glucose-6-phosphate dehydrogenase 2) |  |  |  | **-** |
| 25 | Ghi.1456.1.S1_x_at | CA993106 | Fructose-bisphosphate aldolase |  | **-** |  | **-** |
| 26 | GhiAffx.52253.1.S1_s_at | DR463559 | Pyrophosphate--fructose-6-phosphate 1-phosphotransferase beta subunit / pyrophosphate-dependent 6-phosphofructose-1-kinase |  |  |  | **-** |
| 27 | Ghi.2039.2.S1_s_at | DT463218 | SUS1 (Sucrose synthase 1) |  | **-** |  | **-** |
| 28 | Ghi.1151.1.A1_at | U58283.1 | Cellulose synthase |  |  |  | **-** |
| 29 | Ghi.4648.3.A1_x_at | CO493453 | CESA8 (Cellulase synthase 8) |  |  |  | **-** |
| 30 | Ghi.7294.1.S1_s_at | AI731843 | ATCSLA02 (Cellulose synthase-like A2) |  |  |  | **-** |
| 31 | GhiAffx.21602.1.A1_at | DW496092.1 | ATCSLC05 (Cellulose synthase-like C5) |  |  |  | **-** |
| 32 | Ghi.8147.1.S1_at | DQ073046.1 | Pectate lyase | **+** |  | **-** | **-** |
| 33 | Ghi.10256.1.S1_at | DT567984 | Pectate lyase family protein |  | **-** |  | **-** |
| 34 | Ghi.8137.1.A1_at | DT050714 | Cottonseed isocitrate lyase (ICL) (EC 4.1.3.1) | **-** |  |  | **-** |
| 35 | Ghi.4943.1.A1_x_at | DT049835 | ACLA-3 (ATP-citrate lyase A-3) |  |  |  | **-** |
| 36 | Ghi.1876.1.S1_s_at | DV849686 | Aconitate hydratase / citrate hydro-lyase/aconitase |  |  |  | **-** |
| 37 | Ghi.8277.1.S1_s_at | AI731105 | HTH (HOTHEAD); aldehyde-lyase |  | **-** | **-** | **-** |
| 38 | GhiAffx.25489.1.A1_s_at | DW232960.1 | MDH (malate dehydrogenase); malate dehydrogenase | **-** | **-** |  | **-** |
| 39 | Ghi.7604.1.S1_s_at | DT573609 | 3-isopropylmalate dehydrogenase |  | **-** |  | **-** |
| 40 | Ghi.1646.1.S1_s_at | DN760180 | DCT/DIT2.1 (Dicarboxylate transport); oxoglutarate:malate antiporter |  | - |  | - |
| 41 | GhiAffx.5436.1.S1_at | DW489709.1 | PMDH2 (Peroxisomal NAD-malate dehydrogenase 2) |  |  |  | - |
| 42 | Ghi.10436.1.S1_s_at | DN757648 | ATNADP-ME1 (NADP-MALIC ENZYME 1) |  |  |  | - |
| 43 | Ghi.1663.1.S1_s_at | DN760118 | Phosphoenolpyruvate carboxylase (PEPC2) | - | - |  |  |
| 44 | Ghi.10173.1.S1_s_at | DT466176 | Phosphoenolpyruvate carboxykinase (ATP)/ PEP carboxykinase/ PEPCK |  | - | + |  |

(**+**) indicates up-regulated transcripts

(**-**) indicates down-regulated transcripts

(+/-) indicates differentially regulated transcripts
