# Supplementary material for: Genome‐wide transcriptomic and proteomic analyses of bollworm‐infested developing cotton bolls revealed the genes and pathways involved in the insect pest defence mechanism
Source: Plant Biotechnol J. 2016 Jan 22;14(6):1438–55. doi: 10.1111/pbi.12508 (PMC5066800; doi:10.1111/pbi.12508)
Supplement: Supplementary file 11 — Table S10 Expression pattern of transcripts related to fatty acid metabolism. [file PBI-14-1438-s007.doc]

| **Supporting table S10**  Expression pattern of transcripts related to fatty acid metabolism | | | | | | | |
| --- | --- | --- | --- | --- | --- | --- | --- |
| **S. No** | **Probeset ID** | **Accession No** | **Gene name** | **Boll developmental stages (dpa)** | | | |
|  |  |  |  | **0** | **2** | **5** | **10** |
| 1 | Gra.2105.1.A1_s_at | CO123877 | ACBP1 (Acyl-CoA binding protein) | **+** | **+** | **+** | **+** |
| 2 | Ghi.8009.2.A1_s_at | DR460320 | Triacylglycerol lipase |  |  |  | - |
| 3 | Ghi.6141.1.S1_at | DR456515 | SDP1 (SUGAR-DEPENDENT1); Triacylglycerol lipase | **+** | **+** | **+** | **+** |
| 4 | Ghi.10670.1.S1_x_at | DN779635 | PAD4 (PHYTOALEXIN DEFICIENT 4); Triacylglycerol lipase | **+** |  | **+** | **+** |
| 5 | GhiAffx.31068.1.S1_at | DT466441 | TAG1 (Triacylglycerol biosynthesis defect 1); Diacylglycerol O-acyltransferase | **+** |  | **+** | **+** |
| 6 | GhiAffx.63008.1.S1_s_at | DW504223.1 | Acyl CoA reductase | **+** | **+** | **+/**- | - |
| 7 | GhiAffx.55382.1.S1_at | DW483071.1 | ACX2 (Acyl-CoA oxidase 2); Acyl-CoA oxidase | **+** |  | **+** | **+** |
| 8 | Ghi.8572.1.S1_at | AI055642 | ACX3 (Acyl-CoA oxidase 3); Acyl-CoA oxidase | **+** |  | **+** | **+** |
| 9 | Ghi.7466.1.S1_at | AI730393 | ACX4 (Acyl-CoA oxidase 4); Oxidoreductase | **+** |  | **+** | **+** |
| 10 | GhiAffx.7633.1.S1_s_at | DW499904.1 | FATB (Fatty acyl-ACP thioesterases B); Acyl carrier/ Acyl-ACP thioesterase | **+** | **+** | **+** | **+** |
| 11 | Ghi.3184.1.S1_s_at | DT468147 | AAE7/ACN1 (Acyl-activating enzyme 7); AMP binding / Acetate-CoA ligase | **+** | **+** | **+** | **+** |
| 12 | GhiAffx.844.1.A1_x_at | CO492611 | Beta-ketoacyl-CoA synthase |  | **+** |  | **+** |
| 13 | Ghi.5184.1.A1_s_at | DT048714 | Beta-ketoacyl-CoA synthase (KCS2) |  |  | - | - |
| 14 | GhiAffx.29745.1.S1_s_at | DW482448.1 | Beta-ketoacyl-CoA synthase family protein |  | - | **+/**- | - |
| 15 | Ghi.6416.1.A1_at | DT468316 | ATGPAT7/GPAT7 (Glycerol-3-phosphate acyltransferase 7); 1-acylglycerol-3-phosphate O-acyltransferase/ acyltransferase |  | **+** |  | **+** |
| 16 | GhiAffx.25722.1.A1_s_at | DT463796 | Acetyl-CoA C-acyltransferase / 3-ketoacyl-CoA thiolase |  |  | **+** | **+** |
| 17 | Ghi.858.1.S1_at | DR453910 | LACS7 (Long-chain acyl-CoA synthetase 7) |  |  | **+** | **+** |
| 18 | Ghi.9161.1.S1_at | DT464214 | Acyl-activating enzyme 12 (AAE12) |  | **+** |  |  |
| 19 | Ghi.10704.1.S1_s_at | AY038061.1 | 3-ketoacyl-CoA thiolase |  |  |  | **+** |
| 20 | GhiAffx.25722.1.A1_s_at | DT463796 | Acetyl-CoA C-acyltransferase / 3-ketoacyl-CoA thiolase |  |  | **+** | **+** |
| 21 | Ghi.7523.1.S1_s_at | AI729651 | Lecithin:cholesterol acyltransferase family protein / LACT family protein |  |  | **+** | **+** |
| 22 | GhiAffx.20824.1.S1_x_at | DW233308.1 | Sterol carrier protein 2 (SCP-2) family protein |  |  |  | **+** |
| 23 | GhiAffx.19570.1.S1_at | DW489734.1 | PLP2 (PHOSPHOLIPASE A 2A); Nutrient reservoir | **+** | **+** | **+** | **+** |
| 24 | GhiAffx.21096.1.S1_at | DW225202.1 | Lipase |  |  |  | **-** |
| 25 | Ghi.1889.1.S1_at | DV849586 | Lipase class 3 family protein | **+** | **+** | **+** | **+** |
| 26 | Ghi.3711.1.A1_at | DT461581 | Phospholipase D gamma 2 / PLD gamma 2 (PLDGAMMA2) | **+** |  | **+** | **+** |
| 27 | Ghi.140.1.A1_s_at | DT046624 | GDSL-motif lipase/hydrolase family protein | +/- | +/- | +/- | +/- |
| 28 | Gra.1494.1.S1_s_at | CO127128 | Phospholipase C |  |  | **+** | **+/**- |
| 29 | GhiAffx.10582.1.S1_x_at | DW485100.1 | Carboxylic ester hydrolase |  |  | **+** | **+/**- |
| 30 | GhiAffx.52546.3.S1_at | AI054484 | GLIP1 (GDSL LIPASE1); Carboxylic ester hydrolase |  |  | **+** | **+** |
| 31 | GraAffx.28884.1.A1_s_at | CO125859 | Acyltransferase/ Carboxylic ester hydrolase/ Lipase |  |  |  | - |
| 32 | Ghi.8094.1.S1_s_at | AY138251.1 | Phospholipase D delta isoform 1b |  |  |  | **+** |
| 33 | Ghi.1766.1.S1_s_at | AY138252.1 | Phospholipase D delta isoform 1a | - | - |  | - |
| 34 | Gra.1673.1.S1_at | CO129315 | ATPLDDELTA; phospholipase D |  |  |  | **+** |
| 35 | Ghi.10696.1.S1_s_at | DT048320 | Phosphoinositide phospholipase C |  |  |  | **+** |
| 36 | Ghi.9734.1.S1_at | AW186914 | Long-chain-alcohol O-fatty-acyltransferase family protein / Wax synthase family protein | - | - | - | - |
| 37 | Ghi.10482.1.S1_s_at | DN758513 | Acylaminoacyl-peptidase-related | - |  |  | - |
| 38 | Ghi.9531.1.S1_s_at | DQ122189.1 | 3-ketoacyl-CoA synthase | - |  | - | - |
| 39 | Ghi.5363.1.S1_x_at | DT048064 | 3-ketoacyl-CoA synthase 6 | - |  | - | - |
| 40 | GhiAffx.29745.1.S1_s_at | DW482448.1 | Beta-ketoacyl-CoA synthase family protein |  | - | **+** | - |
| 41 | Ghi.561.1.S1_at | AI729491 | B-keto acyl reductase, putative (GLOSSY8) |  | - | - | - |
| 42 | GhiAffx.1245.1.A1_s_at | DW499446.1 | KAS I (3-ketoacyl-acyl carrier protein synthase I); fatty-acid synthase |  | - | - | - |
| 43 | Ghi.10022.2.S1_s_at | DT564348 | 24-sterol C-methyltransferase (SMT2-2) | - | - | - | - |
| 44 | Ghi.9045.1.A1_s_at | DT048514 | SMO2-2 (sterol 4-alpha-methyl-oxidase 2); C-4 methylsterol oxidase |  | - |  | - |
| 45 | GhiAffx.62156.1.S1_x_at | DW513352.1 | Sterol delta-7 reductase DWF5 |  |  |  | - |
| 46 | Ghi.10736.1.S1_at | DT456470 | SMO1-1 (Sterol-4alpha-methyl oxidase 1-1); catalytic |  |  |  | - |

(**+**) indicates up-regulated transcripts

(**-**) indicates down-regulated transcripts

(+/-) indicates differentially regulated transcripts
