# Supplementary material for: Genome‐wide transcriptomic and proteomic analyses of bollworm‐infested developing cotton bolls revealed the genes and pathways involved in the insect pest defence mechanism
Source: Plant Biotechnol J. 2016 Jan 22;14(6):1438–55. doi: 10.1111/pbi.12508 (PMC5066800; doi:10.1111/pbi.12508)
Supplement: Supplementary file 12 — Table S11 Expression pattern of transcripts related to protein metabolism. [file PBI-14-1438-s008.doc]

| **Supporting table S11** Expression pattern of transcripts related to protein metabolism | | | | | | | |
| --- | --- | --- | --- | --- | --- | --- | --- |
| **S.No** | **Probeset ID** | **Accession No** | **Gene name** | **Boll developmental stages (dpa)** | | | |
|  |  |  |  | **0** | **2** | **5** | **10** |
| 1 | GhiAffx.62022.1.S1_at | DW512578.1 | NS2 (Asparaginyl-tRNA synthetase 2) |  |  |  | **+** |
| 2 | Gra.2101.1.A1_s_at | CO111981 | Protein binding / Ubiquitin-protein ligase/ Zinc ion binding |  | **+** | **+** | **+** |
| 3 | GhiAffx.15780.1.A1_s_at | DW224570.1 | RMA1 (Ring finger protein with Membrane Anchor 1); Protein binding / Ubiquitin-protein ligase/ Zinc ion binding | **+** | **+** | **+** | **+** |
| 4 | GhiAffx.13045.1.S1_at | DW226240.1 | PUB17 (PLANT U-BOX17); Ubiquitin-protein ligase | **+** | **+** | **+** | **+** |
| 5 | GhiAffx.61004.1.S1_at | DW510847.1 | UBC28; Ubiquitin-protein ligase | **+** |  | **+** |  |
| 6 | Ghi.5035.1.A1_at | DT049333 | PHO2/UBC24 (Phosphate 2); Ubiquitin-protein ligase |  |  | **+** | **+** |
| 7 | Ghi.6742.1.A1_at | CA992819 | SUM1 (Small ubiquitin-like modifier 1) |  |  |  | **+** |
| 8 | Ghi.1586.1.S1_at | DN760004 | Heavy-metal-associated domain-containing protein | **+** | **+** | **+/**- | **+** |
| 9 | GhiAffx.43092.1.S1_at | DW511433.1 | Heavy-metal-associated domain-containing protein / Copper chaperone (CCH)-related |  |  |  |  |
| 10 | Ghi.8718.1.S1_s_at | AI732009 | Inter-alpha-trypsin inhibitor heavy chain-related |  |  |  | **+** |
| 11 | Ghi.8797.1.S1_s_at | DT047797 | 60S ribosomal protein L5 (RPL5B) | **+** | **+** |  | **-** |
| 12 | Ghi.5665.1.S1_s_at | DT468291 | 60S ribosomal protein L23A (RPL23aB) |  |  |  | **+** |
| 13 | Ghi.1818.1.S1_at | DV850314 | Ribosomal protein S23 CG8415-PA |  | **+** | **+** |  |
| 14 | GhiAffx.32155.1.A1_at | CO498096 | BETA-VPE (Vacuolar processing enzyme beta); Cysteine-type endopeptidase |  | **+** | **+** | **+** |
| 15 | GhiAffx.21532.1.S1_at | CA993875 | Pentatricopeptide (PPR) repeat-containing protein (GUN1) | **+** | **+/**- | **+** | **+** |
| 16 | Ghi.5694.1.A1_x_at | DT046743 | Glycine/proline-rich protein | **+** |  | - | - |
| 17 | Ghi.3542.2.S1_s_at | DT464538 | ATACP5 (Acid phosphatase 5); Acid phosphatase/ Protein serine/Threonine phosphatase | **+** |  | **+** |  |
| 18 | Ghi.9001.1.S1_s_at | CO496755 | PAP8 (Purple acid phosphatase precursor); Acid phosphatase/ Protein serine/Threonine phosphatase |  |  |  | **+** |
| 19 | Ghi.3138.1.S1_s_at | AI054443 | AtSerat2;1 (Serine acetyltransferase 1) | **+** |  | **+/**- |  |
| 20 | GhiAffx.12850.2.S1_at | DW230229.1 | ATSR1 (Serine/Threonine protein kinase 1); Kinase | **+** |  | **+** | **+** |
| 21 | Ghi.4641.1.A1_s_at | DT051740 | PAP10; Protein serine/Threonine phosphatase | **+** | - | **+** |  |
| 22 | GhiAffx.6217.1.S1_at | DW231422.1 | Serine-rich protein-related |  |  | **+** |  |
| 23 | Ghi.1709.1.S1_x_at | DN759807 | Transformer serine/Arginine-rich ribonucleoprotein |  |  | **+** | **+** |
| 24 | Ghi.5445.2.A1_s_at | DT047720 | Beta-Ig-H3 domain-containing protein / Fasciclin domain-containing protein |  |  |  | - |
| 25 | Gra.2835.2.S1_s_at | CO123945 | Asparaginase | - | - |  | - |
| 26 | Ghi.10091.1.A1_s_at | DT543243 | L-asparaginase, putative / L-asparagine amidohydrolase | - | - | - | - |
| 27 | GhiAffx.5071.1.S1_s_at | DW516454.1 | UBC20 (Ubiquitin-conjugating enzyme 20); Ubiquitin-protein ligase |  | - |  | - |
| 28 | Ghi.3366.2.A1_s_at | DT466823 | UBP15 (Ubiquitin-specific protease 15); Ubiquitin-specific protease |  |  | - | - |
| 29 | GhiAffx.5969.1.S1_at | DW236783.1 | UBQ5 (Ubiquitin 5); Protein binding |  |  |  | - |
| 40 | Ghi.4162.1.S1_x_at | DR458738 | UBQ6 (Ubiquitin 6); Protein binding |  |  |  | - |
| 41 | Gra.2999.1.A1_at | CO074707 | Ubiquitin B |  |  |  | - |
| 42 | Ghi.1824.1.S1_at | DV850268 | UBC36; Ubiquitin-protein ligase |  |  |  | - |
| 43 | Gra.1683.2.S1_s_at | CO126705 | Ubiquitin-protein ligase/ Zinc ion binding |  |  |  | - |
| 44 | Ghi.5759.1.S1_s_at | AI731036 | Myosin heavy chain-related | - | - |  | - |
| 45 | Ghi.64.1.S1_x_at | DT567004 | 40S ribosomal protein S2 (RPS2D) |  | - | - | - |
| 46 | Ghi.7776.1.S1_at | DR458635 | 40S ribosomal protein S10 (RPS10B) |  | - |  | - |
| 47 | Ghi.5275.1.S1_s_at | DT048370 | 40S ribosomal protein S17 (RPS17D) |  | - |  | - |
| 48 | Ghi.796.1.S1_s_at | DR454726 | 40S ribosomal protein S16 (RPS16A) |  |  | - | - |
| 49 | Ghi.5741.1.S1_s_at | AI729660 | 40S ribosomal protein S9 (RPS9C) |  |  |  | - |
| 50 | Ghi.366.1.S1_s_at | DT527088 | 40S ribosomal protein S4 (RPS4D) |  |  |  | - |
| 51 | GhiAffx.48425.1.S1_at | DT569994 | 60S ribosomal protein L6 (RPL6A) |  | - |  | - |
| 52 | Ghi.9989.1.S1_at | DT574467 | 60S ribosomal protein L4/L1 (RPL4A) |  | - | - | - |
| 53 | Ghi.8720.1.S1_s_at | AJ513722 | 60S ribosomal protein L12 (RPL12C) |  | - | **+** | - |
| 54 | GhiAffx.6056.1.S1_s_at | DW495927.1 | 60S ribosomal protein L26 (RPL26A) |  | - | - |  |
| 55 | GhiAffx.2291.1.A1_s_at | DT053400 | RPL18 (Ribosomal protein L18); Structural constituent of ribosome |  | - | **+** | - |
| 56 | Ghi.7688.1.S1_s_at | AI727941 | Ribosomal protein L44 isoform b (RL44) |  | - | - | - |
| 57 | Ghi.6960.1.A1_s_at | AJ513645 | 60S ribosomal protein L18A (RPL18aB) |  |  | - |  |
| 58 | Ghi.7414.1.S1_at | DT047629 | 60S ribosomal protein L9 (RPL90B) |  |  |  | - |
| 59 | Ghi.10798.1.S1_s_at | DN826266 | 60S ribosomal protein L30 (RPL30A) |  |  |  | - |
| 60 | Ghi.10791.1.S1_s_at | DN826179 | 60S ribosomal protein L35a (RPL35aC) |  |  |  | - |
| 61 | Ghi.3341.1.S1_at | DT467137 | 60S ribosomal protein L19B |  | - |  | - |
| 62 | Ghi.1102.1.S1_x_at | DN801249 | 60S ribosomal protein L7 (RPL7D) |  |  |  | - |
| 63 | Ghi.987.1.A1_at | DT048605 | 60S acidic ribosomal protein P0 (RPP0A) |  | - |  | - |
| 64 | GhiAffx.25604.1.A1_s_at | DR453975 | 60S acidic ribosomal protein P1 (RPP1A) |  |  |  | - |
| 65 | GhiAffx.911.1.S1_s_at | DW517325.1 | 50S ribosomal protein L21, Chloroplast / CL21 (RPL21) |  |  |  | - |
| 66 | GhiAffx.17656.1.S1_a_at | AJ513179 | 50S ribosomal protein L28, Chloroplast (CL28) |  |  |  | - |
| 67 | GhiAffx.23083.1.A1_s_at | DW228819.1 | CYP77A4 (Cytochrome P450, family 77, subfamily A, polypeptide 4); Oxygen binding | - | - | - | - |
| 68 | Ghi.7315.1.S1_at | AI731625 | Permease |  |  |  | - |
| 69 | Ghi.4233.1.A1_s_at | DT047742 | Xanthine/Uracil permease family protein |  | - |  | - |
| 70 | Ghi.1295.1.S1_s_at | DT550574 | AAP3 (Amino acid permease 3); Amino acid permease |  | - |  |  |
| 71 | Gra.2438.1.S1_at | CO118864 | AAP6 (Amino acid permease 6); Amino acid permease | - | - |  | **+** |
| 72 | Ghi.10072.3.A1_at | AI728603 | Glycine-rich protein | - | **+** |  | **+** |
| 73 | GhiAffx.30723.1.A1_x_at | DW228431.1 | Glycine-rich protein (GRP3) | - | **+/**- |  | - |
| 74 | GhiAffx.24506.1.S1_at | DW496064.1 | Glycine-rich protein / Oleosin | **+** |  | - | - |
| 75 | GhiAffx.12755.2.S1_at | DT465699 | ATGRP2 (Glycine-rich RNA-binding protein 2) |  | - |  | - |
| 76 | Ghi.4411.1.A1_s_at | DT054043 | High-glycine tyrosine keratin-like protein (xl3 gene) |  | - | - | - |
| 77 | Ghi.1875.1.S1_s_at | DV849688 | Glycine cleavage system H protein |  |  |  | - |
| 78 | Ghi.9995.1.S1_s_at | AI726298 | SHM4 (Serine hydroxymethyltransferase 4); Glycine hydroxymethyltransferase |  |  |  | - |
| 79 | Gra.2732.2.S1_s_at | CO089356 | Glycine dehydrogenase (Decarboxylating) |  |  |  | - |
| 80 | Ghi.6423.1.A1_at | CO490711 | ATAUR1 (Ataurora1); Histone serine kinase(H3-S10 specific) / Kinase/ Protein serine/Threonine kinase |  | - | - | - |
| 81 | Ghi.5722.1.A1_at | DT046552 | SLP3 (Subtilisin-like serine protease 3); Subtilase |  | - |  | - |
| 82 | Gra.3066.1.S1_s_at | CO091240 | Phosphoserine transaminase/ Transaminase |  |  | - | - |
| 83 | GhiAffx.5456.1.S1_s_at | DW227724.1 | Serine/Threonine protein phosphatase 2A (PP2A) regulatory subunit B |  |  | - | - |
| 84 | GhiAffx.24591.1.S1_at | DW502202.1 | SCPL40 (Serine carboxypeptidase-like 40); Serine carboxypeptidase |  |  | - |  |

(**+**) indicates up-regulated transcripts

(**-**) indicates down-regulated transcripts

(+/-) indicates differentially regulated transcripts
