# Supplementary material for: Genome‐wide transcriptomic and proteomic analyses of bollworm‐infested developing cotton bolls revealed the genes and pathways involved in the insect pest defence mechanism
Source: Plant Biotechnol J. 2016 Jan 22;14(6):1438–55. doi: 10.1111/pbi.12508 (PMC5066800; doi:10.1111/pbi.12508)
Supplement: Supplementary file 13 — Table S12 Expression pattern of transcripts related to transport mechanism. [file PBI-14-1438-s009.doc]

**Supporting table S12:** Expression pattern of transcripts related to transport mechanism

| **S. No** | **Probeset ID** | **Accession No.** | **Gene name** | **Boll developmental stages (dpa)** | | | |
| --- | --- | --- | --- | --- | --- | --- | --- |
|  |  |  |  | **0** | **2** | **5** | **10** |
| 1 | Ghi.3135.1.S1_at | DT469074 | Proton-dependent oligopeptide transport (POT) family protein | **+/-** | **+/-** | **+/-** | **+/-** |
| 2 | Ghi.3763.1.A1_s_at | DT461952 | Calcium-transporting ATPase, plasma membrane-type/ Ca(2+)-ATPase (ACA12) | **+** | **+** | **+** | **+** |
| 3 | Ghi.3652.1.S1_s_at | DT463174 | AAT1 (Cationic amino acid transporter 1); Cationic amino acid transporter | **+** | **+** | **+** | **+** |
| 4 | Ghi.1847.1.S1_at | DV850045 | ATPTR3/PTR3 (Peptide transporter protein 3); Transporter | **+** |  | **+** | **+** |
| 5 | Ghi.6855.1.A1_s_at | CA992864 | ProT2 (Proline transporter 2) | **+** | **+** | **+** | **+** |
| 6 | Ghi.3605.1.A1_at | DN759972 | AKT1 (*Arabidopsis* K transporter 1) | **+** |  |  | **+** |
| 7 | Ghi.2352.1.A1_at | DT462757 | AMT1;1 (Ammonium transport 1); Ammonium transporter | **+** |  |  |  |
| 8 | Gra.47.3.S1_x_at | CO098885 | MSS1; Carbohydrate transporter/ Sugar porter | **+** | **+** | **+** | **+** |
| 9 | Ghi.9885.1.A1_at | DT464230 | STP1 (Sugar transporter 1); Carbohydrate transporter/ Sugar porter | **+** |  | **+** | **+** |
| 10 | GhiAffx.29457.1.S1_at | DW238000.1 | Transporter-related | **+** |  | **+** | **+/-** |
| 11 | Ghi.10454.1.S1_at | DN801047 | Amino acid transporter family protein | **+** | **+/-** | **+** | **+** |
| 12 | GhiAffx.6053.1.S1_s_at | DW515652.1 | GPT2 (Glucose-6-phosphate/Phosphate translocator 2); Antiporter/ Glucose-6-phosphate transporter | **-** |  | **-** | **-** |
| 13 | GhiAffx.15524.1.S1_s_at | DW238643.1 | ABC transporter family protein | **-** | **-** | **-** | **-** |
| 14 | Ghi.4468.1.A1_s_at | DR463750 | Protein transport protein SEC61 gamma subunit |  | **+** |  | **-** |
| 15 | Ghi.4013.1.A1_at | CO492517 | IRT1 (Iron-regulated transporter 1); Cadmium ion transporter/ Iron ion transporter/ Manganese ion transporter/ Zinc ion transporter |  | **-** | **+** |  |
| 16 | Gra.2819.1.A1_a_at | CO073817 | DRT112; Copper ion binding / Electron transporter |  | **-** |  |  |
| 17 | GhiAffx.15003.1.S1_at | DT046735 | Mitochondrial phosphate transporter |  | **-** |  | **-** |
| 18 | GhiAffx.30159.1.S1_s_at | DW481957.1 | DIT1 (Dicarboxylate transporter 1); Oxoglutarate:malate antiporter |  | **-** |  |  |
| 19 | Gra.2701.4.S1_s_at | CO088099 | Tetracycline transporter |  | **-** |  | **-** |
| 20 | Ghi.6075.1.S1_s_at | DT048502 | COPT1 (Copper transporter 1); Copper ion transporter |  | **-** | **-** |  |
| 21 | GhiAffx.40989.1.S1_s_at | DW483608.1 | APE2 (Acclimation of photosynthesis to environment); Antiporter/ Triose-phosphate transporter |  | **-** |  | **-** |
| 22 | Gra.2694.1.S1_s_at | CO087595 | RNA binding / Nucleic acid binding / Protein transporter |  | **-** | **-** | **-** |
| 23 | Ghi.4565.1.S1_s_at | DT052272 | Choline transporter-related |  | **-** |  | **-** |
| 24 | Gra.47.3.S1_x_at | CO098885 | MSS1; Carbohydrate transporter/ Sugar porter |  |  |  |  |
| 25 | Ghi.9146.2.S1_s_at | DT463838 | ZIFL1 (Zinc induced facilitator-like 1); Tetracycline: hydrogen antiporter/ Transporter |  |  | **+** | **+** |
| 26 | GhiAffx.20514.1.S1_s_at | DW502367.1 | NIP5;1/NLM6/NLM8 (NOD26-like intrinsic protein 5;1); Boron transporter/ Water channel |  |  | **+** |  |
| 27 | Ghi.6515.1.S1_at | CA993259 | SUC2 (Sucrose-proton symporter 2); Carbohydrate transporter/ Sucrose:hydrogen symporter/ Sugar porter |  |  | **+** | **-** |
| 28 | GraAffx.28667.1.A1_s_at | CO085819 | Antiporter/ Drug transporter |  |  | **+** | **+** |
| 29 | GhiAffx.61502.1.S1_at | DW509711.1 | Potassium transporter |  |  | **+** | **+** |
| 30 | GhiAffx.63947.1.S1_s_at | DT465139 | Phosphate transporter |  |  | **+** | **+** |
| 31 | GhiAffx.12651.1.S1_at | DW513188.1 | SULTR3;4 (SULTR3;4); Sulfate transporter |  |  | **+** | **+/-** |
| 32 | GhiAffx.60576.1.S1_at | DW505061.1 | Nitrate transporter (NTP3) |  |  | **+** | **+** |
| 33 | Ghi.634.1.S1_s_at | AY255521.1 | ABC transporter (WBC1) |  |  | **-** | **-** |
| 34 | Ghi.1646.1.S1_s_at | DN760180 | DCT/DIT2.1 (Dicarboxylate transport); Oxoglutarate:malate antiporter |  |  |  | **-** |
| 35 | Ghi.3240.1.A1_at | DT468114 | Hexose transporter |  |  |  | **+** |
| 36 | GhiAffx.11094.1.A1_at | DW516951.1 | LHT1 (Lysine histidine transporter 1); Amino acid transporter |  |  |  | **+** |
| 37 | GhiAffx.30627.1.S1_s_at | DW498369.1 | ZIP4 (Zinc transporter 4 precursor); Cation transporter |  |  |  | + |
| 38 | Ghi.5629.1.A1_s_at | DT463085 | CAT3 (Cationic amino acid transporter 3); Cationic amino acid transporter |  |  |  | + |
| 39 | GhiAffx.14.1.S1_at | DT049646 | ZIP1 (Zinc transporter 1 precursor); Zinc ion transporter |  |  |  | - |
| 40 | Ghi.9364.1.S1_at | DT456499 | KT2 (Potassium transporter 2) |  |  |  | - |
| 41 | GhiAffx.30987.1.A1_at | DW481679.1 | NPSN11 (Novel plant snare 11); Protein transporter |  |  |  | - |
| 42 | Ghi.8859.1.A1_a_at | DR452329 | Monosaccharide transporter |  |  |  | - |
| 43 | Ghi.7632.1.S1_s_at | AI725673 | (Vacuolar ATP synthase subunit B2); Hydrogen ion transporting ATP synthase, rotational mechanism |  |  |  | +/- |

(+) indicates up-regulated transcripts

(-) indicates down-regulated transcripts

(+/-) indicates differentially regulated transcripts
