# Supplementary material for: Genome‐wide transcriptomic and proteomic analyses of bollworm‐infested developing cotton bolls revealed the genes and pathways involved in the insect pest defence mechanism
Source: Plant Biotechnol J. 2016 Jan 22;14(6):1438–55. doi: 10.1111/pbi.12508 (PMC5066800; doi:10.1111/pbi.12508)
Supplement: Supplementary file 14 — Table S13 Expression pattern of transcripts related to oxidative stress. [file PBI-14-1438-s010.doc]

**Supporting table S13** Expression pattern of transcripts related to oxidative stress

| **S. No** | **Probeset ID** | **Accession No.** | **Gene name** | **Boll developmental stages (dpa)** | | | |
| --- | --- | --- | --- | --- | --- | --- | --- |
|  |  |  |  | **0** | **2** | **5** | **10** |
| 1 | GhiAffx.2752.1.S1_at | CA993163 | ATGSTU23 (*Arabidopsis thaliana* glutathione S-transferase (Class tau) 23); Glutathione transferase | **+** | **+** | **+** | **+** |
| 2 | GhiAffx.21849.1.S1_at | DW496541.1 | ATGSTU7 (Glutathione S-transferase 25); Glutathione transferase | **+** |  | **+** | **+** |
| 3 | GhiAffx.61657.1.S1_at | DW510592.1 | ATGSTF12 (Glutathione S-transferase 26); Glutathione transferase |  |  | **+** | **+** |
| 4 | Ghi.779.1.S1_at | DN800052 | ATGSTU18 (Glutathione S-transferase 29); Glutathione transferase |  |  | **+** | **+/**- |
| 5 | Ghi.8550.1.S1_at | CO495268 | ATGSTU19 (Glutathione transferase 8); Glutathione transferase |  | - | - | - |
| 6 | GhiAffx.61579.1.S1_at | DW510158.1 | Glutathione transferase17 |  |  | **+** | **+** |
| 7 | GhiAffx.44356.1.S1_at | DW509246.1 | Glutathione peroxidase |  |  |  | - |
| 8 | GhiAffx.46685.1.S1_s_at | DW496848.1 | ATGPX4 (Glutathione peroxidase 4); Glutathione peroxidase |  |  | **+** | **+** |
| 9 | Ghi.1773.1.S1_s_at | DR457529 | GGT3 (Gamma-glutamyl transpeptidase 3); Gamma-glutamyltransferase/ Glutathione gamma-glutamylcysteinyltransferase |  | **+** |  |  |
| 10 | Gra.508.1.S1_x_at | CO084885 | Lactoylglutathione lyase |  |  |  | - |
| 11 | Ghi.4466.1.S1_a_at | DR454255 | Lactoylglutathione lyase family protein / Glyoxalase I family protein |  | **+** | **+** | **+**/- |
| 12 | GhiAffx.1589.31.S1_at | DW239769.1 | ATGLX1 (Glyoxalase I homolog); Lactoylglutathione lyase |  | - |  | - |
| 13 | Ghi.7301.1.S1_s_at | DR457687 | Allyl alcohol dehydrogenase |  |  |  | - |
| 14 | Ghi.9532.1.A1_s_at | DT046877 | Alcohol dehydrogenase | - | - |  | - |
| 15 | Ghi.8046.1.S1_at | U53702.1 | Alcohol dehydrogenase 1 |  | - | **+** | - |
| 16 | Ghi.2767.1.S1_s_at | AI729704 | ADH2 (Alcohol dehydrogenase 2) | - |  |  |  |
| 17 | Ghi.8044.1.S1_x_at | U49061.1 | Alcohol dehydrogenase 2a |  |  |  | - |
| 18 | Ghi.5815.1.S1_at | U53704.1 | Alcohol dehydrogenase 2b |  |  | **+** |  |
| 19 | Ghi.8047.1.S1_at | DN759650 | Alcohol dehydrogenase 2c |  |  |  | - |
| 20 | Ghi.8056.1.S1_s_at | AF159229.1 | Tissue-type fiber glutathione S-transferase (GST) |  | - | - | - |
| 21 | Ghi.8122.1.S1_s_at | AF515632.2 | Na/H antiporter (NHX1) | **+** |  | **+** |  |
| 22 | Ghi.8156.1.S1_s_at | DQ088821.1 | FeSOD |  | - |  |  |
| 23 | Ghi.1245.2.S1_s_at | DV849563 | SLT1 (Sodium- and lithium-tolerant 1) |  |  | **+** | **+** |
| 24 | Ghi.8153.1.S1_s_at | DQ120514.1 | Chloroplast Cu/Zn superoxide dismutase mRNA; Nuclear gene for chloroplast product |  |  |  | - |
| 25 | Gra.1666.3.A1_at | CO127994 | CAT2 (Catalase 2); Catalase |  |  |  | **+** |
|  | Gra.1457.1.S1_at | CO124678 | AOS (Allene oxide synthase); Hydro-lyase/ Oxygen binding | **+** | **+** | **+** | **+** |
| 26 | GhiAffx.30499.1.S1_at | DW514660.1 | ATSEH (*Arabidopsis thaliana* soluble epoxide hydrolase); Epoxide hydrolase | **+** | **+** | **+** | **+** |
| 27 | Gra.2738.1.A1_x_at | CO106787 | ATSEH; Catalytic/ Epoxide hydrolase/ Hydrolase |  |  |  | **+** |
| 28 | Ghi.5619.1.A1_at | DT047141 | Epoxide hydrolase |  |  |  | - |
| 29 | Ghi.2639.1.S1_s_at | DT526911 | HPL1 (Hydroperoxide lyase 1); Heme binding / Iron ion binding / Monooxygenase | - |  |  |  |

(**+**) indicates up-regulated transcripts

(**-**) indicates down-regulated transcripts

(+/-) indicates differentially regulated transcripts
